# Natural Product Discovery and Biological Prospecting of Asteraceae Species in Mexico over the Last 30 Years

**DOI:** 10.3390/plants15213487

**Published:** 2026-11-08

**Authors:** Xitlalick García-Nava, Miguel Fuentes-Figueroa, Rosario Redonda-Martínez, Laura Sepúlveda-Cuellar, Joyce Trujillo, Elihú Bautista

**Affiliations:** 1 SECIHTI-División de Biología Molecular, Instituto Potosino de Investigación Científica y Tecnológica A. C., San Luis Potosi 78216, San Luis Potosi, Mexico; xgnava@hotmail.com (X.G.-N.); miguel.fuentes@ipicyt.edu.mx (M.F.-F.); 2 Instituto de Ecología A. C., Red de Diversidad Biológica del Occidente Mexicano, Patzcuaro 61600, Michoacan, Mexico; 3 SECIHTI-División de Materiales Avanzados, Instituto Potosino de Investigación Científica y Tecnológica A. C., San Luis Potosi 78216, San Luis Potosi, Mexico; ana.sepulveda@ipicyt.edu.mx (L.S.-C.); daniela.trujillo@ipicyt.edu.mx (J.T.)

**Keywords:** Mexican flora, edible and medicinal plants, phytochemicals, pharmacological properties, agrochemicals

## Abstract

In Mexico, Asteraceae is the most diverse plant family in terms of number of species, with an occurrence of 3127 species, of which 3063 are considered native and 1996 are endemic. For this reason, the country is considered as a center of distribution and endemism. Mexican Asteraceae plants are characterized by their edible and medicinal usages for the treatment of infectious and chronic degenerative diseases, as well as for their agrochemical potential. Phytochemicals from these species have been of interest for decades and are still being considered as current sources of natural products with high structural diversity and bioactivity. The chemical constituents derived from these plants include alkaloids, flavonoids, phenolics, and terpenoids. The reported biological activities were selected according to predefined eligibility criteria. Of these, the terpenoids represent the most relevance due to the number of chemical structures described and their bioactivity as anti-inflammatory, antiprotozoal, antitumor, cytotoxic and phytotoxic agents. In this scoping review, we present the results of a bibliographic revision, and its subsequent analysis to illustrate the state of the art in natural product discovery and pharmacological prospecting with Asteraceae species native to Mexico over the last 30 years. This review highlights the chemical structural diversity of the Asteraceae family members and provides a selective overview of the agrochemical and pharmacological potential of their specialized metabolites, identifying gaps in the subject matter and trends for future research.

## 1. Introduction

The Asteraceae family consists of 10% of the angiosperms distributed around the globe, with an estimated range of 25,000–30,000 species, grouped in 50 tribes and 1900 genera [1,2]. In Mexico, this plant family is also the most diverse of the angiosperms in terms of the number of species, and the country is considered a center of distribution and endemism. Asteraceae plants distributed in Mexico are grouped into 26 tribes and 428 genera. From a total of 3127 species, 3063 are considered native, 1996 are endemic, and 64 have been introduced. The Astereae, Coreopsideae, Eupatorieae, Heliantheae, Millerieae, Senecioneae and Tageteae tribes group the majority of species. Of these, Eupatorieae and Heliantheae are constituted by 623 and 644 species, respectively, followed by Astereae, Senecioneae, Coreopsideae, Millerieae and Tageteae with 382, 227, 202, 175, and 173 species, respectively [3]. The main phenotypic feature for identifying Asteraceae plants is their inflorescences, which resemble a floret and are named “heads”. This is due to the large number of tiny flowers that develop on the receptacle [3,4].

The members of the Asteraceae family have served as sources of specialized bioactive metabolites for the development of pharmacological agents with clinical applications, as well as compounds with agrochemical and alimentary uses. This has been possible due to the application of several approaches, which have consisted of the empirical observation of the properties of some plants (e.g., allelopathy), their traditional medicinal uses, the research programs to identify bioactive constituents derived from plants for the treatment of a specific disease, the approaches for identification of targeting compounds against a specific molecular target, or a conjunction of these. Thus, the systematic assaying of sesquiterpene lactones, the main kind of specialized metabolites derived from Asteraceae species, against cancer cells lines, and in vivo antitumor assaying, have allowed the classification of these kinds of natural products as Michael acceptors due to the presence of an α-methylene-γ-lactone moiety in their structures, and have allowed some of them to reach clinical trials for cancer treatment [5]. Other structural moieties involved in the cytotoxic and antitumor properties of sesquiterpene lactones are an α,β-unsaturated cyclopentenone, an α-epoxycyclopentenone, and/or ester groups in distinct positions of the carbonated frameworks (e.g., eudesmanolides, germacranolides, guaianolides and psuedoguaianolides). Helenalin (**1**) and parthenolide (**2**) are two sesquiterpene lactones used in clinical trials to treat several kinds of human carcinomas and have inspired the semi-synthesis of several analogs for the same purposes (Figure 1), to improve their pharmacokinetics and to reduce side effects (e.g., their allergenic capability) [5]. Helenalin (**1**) is obtained from species of the genera *Artemisia*, *Arnica* and *Inula*, while parthenolide (**2**) occurs in species of the *Tanacetum* genus [6,7]. Another feature of sesquiterpene lactones is that they act in cellular environments via multiple targets, including the inhibition of key proteins involved in biological processes such as cellular proliferation, inflammation, metabolism and oxidative stress. Some molecular targets that helenalin (**1**) and parthenolide (**2**) inhibit are NF-kB, human telomerase, and STAT-3, among others. Similarly, sesquiterpene lactones can activate molecular targets such as ERK and PKC [8,9].

*Stevia rebaudiana* is a native perennial arbustive plant from South America with culinary and medicinal uses. Its aerial parts have been used as a sweetener in “mate”, an infusion prepared with the leaves of *Illex paraguariensis*, commonly consumed in Argentina and Paraguay. Ethnomedical uses described for the plant include treatment of cancer, diabetes, and hypertension, as well as antimicrobial, anti-inflammatory and contraceptive effects [10,11]. Stevioside (**3**) and rebaudioside A (**4**) are the major *ent*-kaurane glycoside diterpenoids reported for *S. rebaudiana* (Figure 1), and both compounds are responsible for the sweetness properties of the plant. Nowadays, demand for cultivation of this plant is increasing to obtain extracts and preparations used for foods and beverages with reduced caloric content [12].

The development of the pyrethroids began with studies of the insecticidal properties of *Tanacetum cinerariifolium* (Syn. *Chrysanthemum cinerariifolium*), an ornamental plant with white flowers known as pyrethrum. Pyrethrum powder has been used for insecticidal applications since the 17th century. In turn, the first oil-based pyrethrum formulations began to be sold in the early 20th century, prompting the emergence of household insecticides. Pyrethrins I (**5**) and II (**6**) are the main constituents (ca. 0.40 and 0.05% wt, respectively) in the mature flower heads of *Tanacetum* species. Other natural analogs with insecticidal activity include cinerins I (**7**) and II (**8**), and jasmolins I (**9**) and II (**10**). Bioallethrin (**11**) was the first synthetic pyrethroid and is still used in several insecticide formulations worldwide, for both agricultural and household purposes (Figure 1) [13,14]. In this review, we present the results of a bibliographic revision and its subsequent analysis to illustrate the state of the art in natural product discovery and pharmacological prospecting with Asteraceae species native to Mexico over the last 30 years.

## 2. Methodology

A search on scientific literature databases ScienceDirect and Scopus using the following keywords: “Asteraceae in Mexico”, “phytochemistry”, “natural products”, “agrochemical properties” and “pharmacological properties” was carried out. The records obtained were complemented by consulting Google Scholar, PubMed and SciELO, using a specific common or scientific plant name, to obtain registers of research articles published in Latin American journals. This search comprises the period between the years 1996 to the first half of 2026. Additional ethnomedicinal information of the plants listed was acquired from texts such as *Las Plantas Medicinales de Mexico* [15], *Atlas de las Plantas de la Medicina Tradicional Mexicana* [16], and from the web platforms “Enciclovida” [17] and “Malezas de México” [18]. The common names and traditional uses of the plants were obtained from some regional floras (*Flora Fanerogámica del Valle de México* [19], *Flora Ilustrada de la Reserva de la Biosfera Mariposa Monarca* [20], *Flora Mesoamericana* [21] and *Flora of North America* [22]), as well as from labels of herbaria specimens physically consulted in the Herbario Nacional de México, Instituto de Biología, UNAM (MEXU) and Herbario de la Escuela Nacional de Ciencias Biológicas, IPN (ENCB); we also reviewed the Herbario de Plantas Ornamentales, Facultad de Arquitectura, UNAM (HEFA) and MEXU, acronyms cited according to Index Herbariorum [23], with the last two also being available in the repository Portal de Datos Abiertos, UNAM [24]. The criteria for selection of Natural Product Discovery were based on a full structural characterization or an identification that involved the use of contemporary analytical techniques such as GC-MS, LC-MS, 1D/2D NMR, HRMS and SCXRD, and were only considered native and/or endemic species to Mexico. Native plants are those with a defined geographic origin—such as a country or continent—for example, the genus *Dahlia*, which is native to Mexico and Central America [25,26], while endemic species are those with a distribution restricted to a specific country, state, geographic region, mountain range, soil type, etc., and are not found anywhere else in the world, for example, the genus *Mixtecalia*, which is found solely in the Mixteca region of Oaxaca, Mexico [25,27]. The biological properties of plants, extracts and pure compounds were chosen or considered “eligible” accordingly: (1) Experimental models that can be considered reproducible. This means that studies were only selected if they clearly evidenced an experimental design with an appropriate number of replicates and statistical treatment of the obtained data, and they were compared against a drug or reference compound used as positive control. (2) Studies that contain data on significant bioactivity or positive bioactivity effects. Studies that described the antioxidant activity of phenolics were discarded as they were considered “predictable”. Likewise, studies that described biological activity in the range of 1–2 orders of magnitude compared with the positive control were selected as “significant”. For example, for cytotoxic activity, an IC_50_ value less than 10 µM was considered cytotoxic, while an IC_50_ value in the range of 11 to 25 µM was moderately cytotoxic, and an IC_50_ value in the range of 26 to 50 µM was considered as showing weak cytotoxicity. (3) An appropriate identification of natural products, which could consist of one of the following identification levels: “unequivocal”, considering their molecular structure and their absolute stereochemistry; “fully”, only considering their molecular structure determination, where both identification levels are supported by 1D/2D NMR, HRMS and SCXRD analyses structural elucidation; “comparative”, which consisted of the comparison of the physical, spectroscopic and spectrometric properties by at least one analytical technique for the same compound in at least two references; and “putative”, which covered an identification of specialized metabolites in extracts through hyphenated methodologies such as GC-MS and LC-MS.

## 3. Results and Discussion

The literature search generated 263 scientific reports from databases, two books, two websites, four regional floras and the specimens deposited in three herbaria. Of these, only those that fall within the filters described in the Methodology Section were considered. Therefore, in the period covered, 94 Asteraceae plant species with relevance for their edible and medicinal uses were identified, a total of 494 specialized metabolites derived from them, and thirteen types of pharmacological properties and four kinds of bioactivity associated with agrochemical models have been reported. For greater understanding, the results obtained were divided into four sections: edible and ethnomedicinal uses, phytochemistry, pharmacological properties and agrochemical applications. Each section is discussed in detail below.

### 3.1. Edible and Ethnomedicinal Uses

In the last 30 years, 94 species have been reported with edible and traditional medicinal uses in 86 literature references that also accomplished structural elucidation and quantification approaches of their specialized metabolites (Table 1). Of these, 53 are native and 38 are endemic. Treating stomachache is the main medicinal use for these plants (21 species), where the *Ageratina* (11 species) and *Stevia* (six species) genera are those with the largest number of species used for this purpose. The second use of the Asteraceae species was on gastrointestinal disorders (GIs) not related to pain, such as diarrhea, digestive problems, purgatives, intestinal helminths, stomach ulcers, gastritis, vomiting, and dysentery. The genera with the largest number of species to treat GI disorders are *Tagetes*, followed by *Calea* and *Brickellia*. In addition, the use of 20 species was described to treat respiratory issues like cough, chill, flu, asthma, cold, bronchitis, and pertussis, highlighting again species belonging to the *Tagetes* genus. The *Calea* species are employed for skin problems, as well as to treat urinary problems. To treat diabetes mellitus (DM), two species of *Calea* and three of *Psacalium* have been used. The species used to treat liver problems are *Artemisia absinthium*, *Laennecia filaginoides* (reported as *Conyza filaginoides*), *Taraxacum officinale*, *Iostephane heterophylla*, and *Calea ternifolia*. The Asteraceae species with effects on the central nervous system are *Achillea millefolium*, *Artemisia absinthium*, *Baccharis conferta*, *Hofmeisteria schaffneri*, and *Calea ternifolia*. Wound healing is another ethnomedical use of the plants from the Asteraceae family, with 16 species reported for this purpose, mainly of the genera *Pittocaulon* and *Tagetes*. Only *Calea ternifolia*, *C. urticifolia*, and *Artemisia ludoviciana* subsp. *mexicana* have been used to treat malaria. In summary, the genus most commonly used in ethnomedicine in Mexico, as reported in the literature, is Tagetes. The principal preparation methods of Asteraceae plants to treat the illness were infusion, decoction, and tincture. The parts of the plants used are leaves, whole plant, flowers, roots, branches, stems, aerial parts, young leaves, capitulum, bark, and, in a few plants, buds, wood, sap, and heads. There are some specific methods for use of plants like *Acourtia humboldtii* (reported as *Perezia adnata*) in small pieces mixed with “pulque”, a traditional Mexican alcoholic beverage, or chewing *Heliopsis longipes* to produce a sedative effect in the mouth [28,29,30].

*Heliopsis longipes* has been traditionally used as a condiment in sauces, stews, and traditional alcoholic beverages in the states of Guanajuato and San Luis Potosí [30]. Likewise, there are records of the use of *T. lucida* dating back to pre-Hispanic times; in addition to its medicinal properties, this species is used as a condiment and in the preparation of infusions used to relieve stomach pain [31]. *Tagetes erecta*, commonly known as “cempasúchil” or Mexican marigold, stands out not only for its ornamental value and cultural significance in Mexico, particularly during the traditional celebration of the Day of the Dead, but also for its growing scientific biotechnological applications. Its inflorescences represent one of the richest natural sources of xanthophylls, particularly lutein, a pigment widely used in the food industry to color products such as eggs, chicken, baked goods, cereals, beverages, dairy products, and sauces. Similarly, the flowers of *Dahlia coccinea* and *Dahlia pinnata* are used in salads, creams, cheeses, and sauces, while their tubers, rich in inulin and fructose, are used to prepare various beverages [32]. Furthermore, in Mexico, numerous herbaceous species known as “quelites” have been part of the pre-Hispanic diet and traditional Mexican diet; among the Asteraceae, *Porophyllum linaria* (registered as *Porophyllum tagetoides*), *Porophyllum ruderale* var. macrocephalum (reported as *Porophyllum macrocephalum*), and *Cotula mexicana* stand out and are traditionally used as edible and aromatic plants [33].

Furthermore, the oil extracted from *Helianthus annuus* seeds is widely used to produce cooking oil, as a snack, and in hair care products for its richness in phenolic compounds, including caffeic acid, chlorogenic acid (**113**), caffeoylquinic acids, methyl chlorogenate (**114**), and eriodictyol glucoside [34].

**Table 1:** List of common names of Asteraceae plants and their edible and traditional medicinal uses.

	Tribe	Valid Name	Registered Name	Common Names	Edible and Traditional Medicinal Uses	Part Used	Reference
1	Anthemideae	*Achillea millefolium* L.*	*Achillea millefolium*	alcanfor, alhucema, cientoenrama, cola de ardilla, cola de pavo, encaje, gordolobo, lumajilla (Tzeltal), lumajiya (Tzeltal), mil en rama, milenrama, plumajillo, ne kotz’ (Tzotzil), plumajilla, plumajillo, plumajo, plumilla, real del oro, simonillo, tlalistagia	stomachache, menstrual pain, tooth ache, earache, bile problems, diarrhea, pimples, bleeding, cough	branches and leaves	[19,21,35,36], MEXU, HEFA
2	Anthemideae	*Artemisia absinthium* L.**	*Artemisia absinthium*	ajenjo, estafiate, hierba maestra, hierba maistra, maestra, maistra, prodigiosa.	bile problems, colic, stomach pain, gastrointestinal disorders, liver ailments, and increase appetite	aerial parts, leaves, whole plant	[21,37], MEXU
3	Anthemideae	*Artemisia ludoviciana* Nutt. subsp. *mexicana* (Willd. ex Spreng.) D.D. Keck *	*Artemisia ludoviciana* subsp. *mexicana*	ajenjo, ajenjo del país, estafiate, incienso de monte, iztauhyatl, macho, telkox	antihelmintic, bile problems, malaria	ND	[19,21,38], MEXA, HEFA
4	Anthemideae	*Matricaria chamomilla* L. **	*Matricaria chamomilla*	guiÊe-mànzànÒ (Zapoteco), manzanilla, manzanilla cimarrona, manzanilla común, manzanilla criolla, mantzaniya (Tzotzil), mantzaniyo (Tzotzil)	topical antiseptic, colic, menstrual cramps, stomach pain, chills, fever, digestive disorders	leaves, young leaves, aerial parts, whole plant	[19,21,39], MEXU
5	Astereae	*Baccharis conferta* Kunth *	*Baccharis conferta*	cerro chiquito, escoba, escobilla, escobilla del carbonero, hierba del carbonero, tepopote, xcacháte (mazateco), xcache (mazateco), xcacna (mazateco), xcodjo (mazateco), yu cu tabi (mixteco)	ceremonial uses, chest ailments, flu, diabetes, diarrhea, headache, epilepsy	young leaves, branches	[19,20,40], MEXU
6	Astereae	*Gymnosperma glutinosum* (Spreng.) Less. *	*Gymnosperma glutinosum*	chamizo, escobilla, hoja pegajosa, jarilla, la diiti (zapoteco), pegajosa, popote; popotillo, tatalencho], tnu-yuji, yucundede, zazal	heal fractures and sprains, remove melasma, cough, ritual uses, treat evil wind in children	young leaves, flowers, branches	[19,35,41,42], MEXU
7	Astereae	*Heterotheca inuloides* Cass. ***	*Heterotheca inuloides*	acáhuatl, árnica, árnica corriente	relief symptoms associated to gastric ulcers, intense pain, bruises, pimples, wounds, inflammation, rheumatism	leaves, branches	[19,43], MEXU
8	Astereae	*Laennecia schiedeana* (Less.) G.L. Nesom *	*Laennecia schiedeana*	simonillo	treat bile problems, stomachache	ND	[44], MEXU
9	Astereae	*Laennecia filaginoides* DC. *	*Laennecia filaginoides (as Conyza filaginoides)*	amargosa, cenizo, gordolobo, ndazuu, simonilla, simonillo	bile problems, stomachache, hepatic illness	roots, whole plant	[19,45], MEXU
10	Astereae	*Machaeranthera tanacetifolia* (Kunth) Ness *	*Machaeranthera tanacetifolia*	árnica, árnica morada, árnica para lavar	ulcers, anti-inflammatory, wash wounds	ND	[46], MEXU
11	Astereae	*Xanthocephalum eradiatum* (M.A. Lane) G.L. Nesom ***	*Xanthocephalum gymnospermoides var. eradiatum*	uriso (rarámuri)		steams	[47], MEXU
12	Cichorieae	*Taraxacum officinale* F.H. Wigg. **	*Taraxacum officinale*	amargón, árnica, ch’ix kul wamal (tzeltal), diente de león, lechuguilla, kaxlan yok’ tz’i’ (tzotzil), yuku nga’a, un’u nga’a	edible as quelite or lettuce, fodder, armpit pain, biliary disorders, dyspepsia, spasms, liver ailments, urinary infections, cough, laxative, tonic	leaves, roots	[19,21,36,48,49,50], MEXU
13	Coreopsideae	*Bidens odorata* Cav. *	*Bidens odorata*	acahual, acahual blanco, aceitilla, aceitillo, chamisa, cisiquelite, (guìzh-)guiÊe-tÓ-nquóts (zapoteco), hierba del pollo, iztziquitl, largo blanco florece, majtas (tzeltal), malva, mozote, rosa de muerto blanca, rosetilla, té de milpa blanco, tzitzitquílitl, yuku sata	edible, fever, inflammation, ulcers, tonic	leaves	[19,48,51,52,53], MEXU
14	Coreopsideae	*Cosmos pringlei* B.L. Rob. & Fernald ***	*Cosmos pringlei*	babisa, barbisa	stomachache	root	[54], MEXU
15	Eupatorieae	*Ageratina cylindrica* (McVaugh) R.M. King & H. Rob. ***	*Ageratina cylindrica*	ND		ND	[55,56,57]
16	Eupatorieae	*Ageratina deltoidea* (Jacq.) R.M. King & H. Rob. ***	*Ageratina deltoidea*	escobilla, xolochíchitl, yolochíchitl		ND	[19,58], MEXU
17	Eupatorieae	*Ageratina glabrata* (Kunth) R.M. King & H. Rob. ***	*Ageratina glabrata*	chamiza, chamizo, hierba del golpe, hierba de la paloma, hilo, jarilla, mixtlácotl, moradillo, uirapin amargosillo, vara blanca	analgesic, bile problems, women’s disease	leaves, branches and stems	[17,20], MEXU [59,60,61], MEXU
18	Eupatorieae	*Ageratina grandifolia* (Regel) R.M. King & H. Rob. ***	*Ageratina grandifolia*	axihutil, copal, quemada	treat skin conditions, stomachache	ND	[62], MEXU
19	Eupatorieae	*Ageratina jocotepecana* B.L. Turner ***	*Ageratina jocotepecana*	ND		ND	[63]
20	Eupatorieae	*Ageratina ligustrina* (DC.) King & H. Rob. *	*Ageratina ligustrina*	tzotz, wamal (Tzetzal), ch’a te’(Tzotzil), ch’il wamal (Tzetzal), bik’tal ch’a te’(Tzotzil), sikil wamal (Tzeltal), muk’tik ch’a te’	constipation, weight loss, chills, heartache, bloody diarrhea	leaves, whole plant	ENCB
21	Eupatorieae	*Ageratina pichinchensis* (Kunth) R.M. King & H. Rob. *	*Ageratina pichinchensis*	capoch, ch’aal wamal (Tzeltal), guìzh-nquòts (Zapoteco), guìzh-nziÛ-dán (Zapoteco), sabal wamal (Tzeltal), sakil wamal (Tzeltal), tzotz wamal (Tzeltal), xuch’ wamal (Tzeltal), yax wamal (Tzeltal)	medicinal uses, eye conditions, heal wounds, diarrhea with mucus, heartache, stomachache, chills	leaves, whole plant	[64,65,66], MEXU
22	Eupatorieae	*Ageratina pringlei* (Rob & Greenm.) King & H. Rob *	*Ageratina pringlei*	pom ch’a te’(Tzotzil)	earache	leaves	ENCB
23	Eupatorieae	*Ageratina vernalis* (Vatke & Kurtz) R.M. King & H. Rob *	*Ageratina subinclusa*	lija te’(Tzotzil)	cramp	flowers	ENCB
24	Eupatorieae	*Ageratina tomentella* (Schrad.) R.M King and H. Rob *	*Ageratina tomentella*	sakil nich wamal (Tzeltal)	illness attributed to exposure to harmful winds or cold air	leaves	ENCB
25	Eupatorieae	*Ageratum houstonianum* Mill. *	*Ageratum houstonianum*	docaj, flor morada, flor noble, lokab, Ma cuen (Chinanteco), mejorana, mejorana chaparra, oxyoket jomol (Tzotzil), palo amarillo, pastorita, pelona del campo, potzlom jomol (Tzotzil), pincel, pinceles, sakilnich yaal wamal, San Lucas, taam ujto, x-naxu-tza-nga’ (Mazateco), xucgo (mazateco), yashal nich wamal	eye pain, stomachache, stomach cramps, remove warts, fractures, wash sores, pimples, non-healing wounds	leaves, young leaves, whole plant	[21,67], HEFA
26	Eupatorieae	*Brickellia cavanillesii* (Cass.) A. Gray ***	*Brickellia cavanillesii*	ND		ND	[68]
27	Eupatorieae	*Brickellia paniculata* (Mill.) B.L. Rob. *	*Brickellia paniculata*	amargoso, amor de barre horno, chirivisca, orégano de monte, uehi ay (Popoluca)	calm crying child	leaves	[21,69], MEXU
28	Eupatorieae	*Brickellia veronicifolia* (Kunth) A. Gray *	*Brickellia veronicifolia*	estrellita, gobernadora, guìzh-yóob-láy (Zapoteco), hierba de la estrellita, kandiakúte (Chocho), oreja de ratón, peshtó	heart palpitations, stomachache, toothache	branches	[70,71], MEXU
29	Eupatorieae	*Chromolaena pulchella* (Kunth) R.M. King & H. Rob. ***	*Chromolaena pulchella*	flor de encaje		ND	[72], MEXU
30	Eupatorieae	*Critoniadelphus nubigenus* (Benth.) King & H. Rob.*	*Critoniadelphus nubigenus*	ch’a te’mut (Tzotzil)	skin condition	leaves	ENCB
31	Eupatorieae	*Decachaeta incompta* (DC.) R.M. King & H. Rob. *	*Decachaeta incompta*	cimarrón zYYwi (Popoluca), tabaco cimarrón	tobacco substitute	leaves	[73,74], MEXU
32	Eupatorieae	*Ageratina petiolaris* (Moc. & Sessé ex DC.) R.M. King & H. Rob. ***	*Eupatorium petiolare*	amargocilla, amargosillo, hierba amargosa, hierba del ángeL, hierba del perro, peistó grande, pesto, tudwl, yolochíchil, yoyochíl	bile reflux	flowers and leaves	[17], MEXU
33	Eupatorieae	*Fleischmannia pycnocephala* (Less.) R.M. King & H. Rob. *	*Eupatorium pycnocephalum*	hierba de cruz, medicina de cruz, ponza Ñ’ethi (otomí)	illness attributed to exposure to harmful winds or cold air, postpartum herbal bath	NA	[75], ENCB
34	Eupatorieae	*Fleischmanniopsis leucocephala* (Benth.) King & H. Rob. *	*Fleischmanniopsis leucocephala*	chilka yantzilet skemal, tzil te’	pain to urinate, wound treatment, headache	leaves, whole plant	ENCB
35	Eupatorieae	*Hofmeisteria schaffneri* (A. Gray) R.M. King & H. Rob.***	*Hofmeisteria schaffneri*	ámbar, manzanilla australiana, té de ámbar	neurological and heart conditions	ND	[76], MEXU
36	Eupatorieae	*Mikania cordifolia* (L. f.) Willd. *	*Mikania cordifolia*	agua boca bejuco hierba, asumiate, catiquini, ch’a ak’ (Tzeltal), chichicastre, cuamecate, hoja de carga, lechosa, tzämy tzoy tzämy ay (Zoque), sac ac, wako (Huasteco)	headache, stomachache, ritual uses, pediatric ailments	aerial parts, flowers, leaves and stems	[77,78]
37	Eupatorieae	*Mikania micrantha* Kunth *	*Mikania micrantha*	amarillo agua camote bejuco, bejuco de criatura, blanco bejuco, blanco florece hierba, guaco, kasi che, luba’, maja k’ur ikir, monte de rastrojo, nama	fever relief, white discharge, snake bite	leaves	[78]
38	Eupatorieae	*Peteravenia phoenicolepsis* (B.L. Rob.) R.M. King & H. Rob. *	*Peteravenia phoenicolepsis*	tz’albil wamal (Tzetzal)	bone pain	leaves	ENCB
39	Eupatorieae	*Stevia eupatoria* (Spreng.) Willd. ***	*Stevia eupatoria*	hierba dulce		ND	[79], MEXU
40	Eupatorieae	*Stevia monardifolia* Kunth ***	*Stevia monardifolia*	ND	ND	ND	[80], MEXU
41	Eupatorieae	*Stevia pilosa* Lag. ***	*Stevia pilosa*	panalito de miel		branches	[81], MEXU
42	Eupatorieae	*Stevia subpubescens* Lag. ***	*Stevia subpubescens*	guìzh-nziÛ or guiÊe-nziÛ (Zapoteco), guìzh-guiÊe-nziÛ (Zapoteco), hoja de tabla, sasal, tlamacas, vara blanca, yerba del golpe (MEXU)	ceremonial uses in marriage	ND	[82], MEXU
43	Eupatorieae	*Stevia tomentosa* Kunth ***	*Stevia tomentosa*	cola de borrego	religious offerings	ND	[83], MEXU
44	Eupatorieae	*Stevia viscida* Kunth *	*Stevia viscida*	jarilla, mosquitos, rosalinda, vara de cohete		ND	[24,79], MEXU
45	Gnaphalieae	*Pseudognaphalium liebmannii* (Sch. Bip. ex Klatt) Anderb. *	*Gnaphalium liebmannii*	gordolobo	respiratory diseases, asthma, bronchitis, bronchial infections and cough	ND	[84], MEXU
46	Heliantheae	*Ambrosia confertiflora* DC. *	*Ambrosia confertiflora*	chi’ichivo (Yaqui), estafiate, guazarape (Guarijio), hierba amargosa, istafiate	intestinal parasites, stomachache and hemorrhoids	ND	[85], MEXU
47	Heliantheae	*Ambrosia cumanensis* Kunth *	*Ambrosia psilostachya*	altamisa, amargosa; ichil ok wamal, sakil wamal, tan wamal, tan yax wamal, tzobtomba		ND	[19,21,86]
48	Heliantheae	*Encelia farinosa* A. Gray ex Torr. *	*Encelia farinosa*	hierba ceniza, incienso, kopal kin (Yaqui)	stomachache	bark	[87], MEXU
49	Heliantheae	*Flourensia cernua* DC. *	*Flourensia cernua*	hojasén, hoja sen	stomachache, veterinary uses	leaves	[88], MEXU
50	Heliantheae	*Gonzalezia hypargyrea* (Greenm.) E.E. Schill. & Panero ***	*Viguiera hypargyrea*	plateado	gastrointestinal disorders	roots	[89], MEXU
51	Heliantheae	*Gonzalezia decurrens* (A. Gray) E.E. Schill. & Paner ***	*Viguiera decurrens*	escorcionera, nakaruri	boils, gastric ulcers infections and wounds	roots	[90], MEXU
52	Heliantheae	*Heliopsis longipes* (A. Gray) S.F. Blake ***	*Heliopsis longipes*	Chilcahue, chicuague, chilcuán, chilmecatl, aztec root and golden root	gingival disease, muscular pain, as spice, to treat parasites and toothaches	roots	[26,91]
53	Heliantheae	*Iostephane heterophylla* (Cav.) Benth. ***	*Iostephane heterophylla*	escorcionera, gentiana del país, girasol, hierba del indio, hierba del manso, manso, raíz de manso	analgesic, wound healing, arthritic pain, insect bites, diabetes, pulmonary, hepatic and gastrointestinal conditions, sores, ulcers	roots	[19,20,92], MEXU
54	Heliantheae	*Montanoa tomentosa* Cerv. *	*Montanoa tomentosa*	chocho, ndllarutxringa tchie, tezontle, toquehua, totopoca blanca, tronadora negra, vara de cama, yag tsín, yagaseti blanco, zoapatle	ceremonial uses, abortifacient, diuretic, stomachache, induce childbirth	leaves, stem, whole plant	[19,93], MEXU
55	Heliantheae	*Parthenium argentatum* A. Gray *	*Parthenium argentatum*	guayule		ND	[22,94], FNA, MEXU
56	Heliantheae	*Tithonia diversifolia* (Hemsl.) A. Gray *	*Tithonia diversifolia*	acahual, acaucli, andán silvestre, árnica, árnica del cerro, árnica extrajera, bár-dóo-nlè (Zapoteco), c’anc’an top robir (Lacandón), cagual amarillo, campana, ch’ajkil (Tzeltal), chaczuum, chajkil (Tzeltal), chajuil (Tzeltal), giganton; girasol, girasol de casa, hog má gé (Chinanteco), ik’al (Tzotzil), k’ayil (Tzotzil), man wich (Huasteco), sum kak; sunkak, tacote, tacotillo, tam chich (Popoluca), tamchicht	anti-inflammatory, antiseptic, wound healing, bile problems, cramps, uterine cancer, dandruff, heartache, stomachache, fever, bruises, pimples, wash wounds, intestinal wash, scabies, medicine bathe infants so they don’t cry or get angry, medicinal use asthma, bronchitis, headache, rheumatism	flowers, leaves, branches, whole plant	[19,77,95], HEFA, MEXU
57	Heliantheae	*Verbesina persicifolia* DC. *	*Verbesina persicifolia*	árnica, flor amarilla, huichín, yucuandayu “yerba rasposa” (Mixteco); wichín	antibiotic, anti-inflammatory, diabetes, reduce liver inflammation, kidney stone dissolution, appendicitis, stomach pain, urinary infections, stomach ulcers	ND	[96], MEXU
58	Heliantheae	*Zinnia grandiflora* Nutt. *	*Zinnia grandiflora*	Plains or Rocky Mountain zinnia		ND	[97], FNA
59	Heliantheae	*Zinnia peruviana* (L.) L. *	*Zinnia peruviana*	arador, chaparrita, gacasal, flor de gallito, gallitos, gallón, guizh-guiÊe-bgÛs (Zapoteco), guiÊe-bgÛs (Zapoteco), hierba de la víbora, mal de ojo, margaritas, margarita de monte; Miguelito, molinillo de monte, ojo de pollo, Peruvian zinnia; San Miguel; Virginia	adornment; decoration; fertilizer; forage; medicinal, wound healing, treat digestive problems	flowers	[19,98], FNA
60	Millerieae	*Desmanthodium fruticosum* Greenm. ***	*Desmanthodium fructicosum*	hoja lisa		ND	MEXU
61	Millerieae	*Jaegeria hirta* (Lag.) Less. *	*Jaegeria hirta*	Sikil uskun (Tzotzil)	diarrhea	flowers	
62	Millerieae	*Melampodium divaricatum* (Rich.) DC. *	*Melampodium divaricatum*	acahual, amarillo florece hierba, andani (Purépecha), botón de oro, cahual, ehkaw (Náhuatl), flor amarilla, flor de estrella, florecilla, guiee-guts, guiee-bniil-las (Zapoteco), hierba amarilla, hoja de azar, ita nu tnu’u kwechi, k’anal nich (Tzeltal), k’anal nich wamal (Tzeltal), k’analnich wamal (Tzeltal), k’antumbu macho, kantun bo, kantun bom, kawal chico, madre maíz, mi:lah ehkaw (Náhuatl), monte amarillo, mozote, mozote amarillo, mozote de cochino, najtos (Huave), pericón, pericusha, periquillo, pus uús, soch (ba), tá-jonal, tomasuchi, totolishtli “hierba de guajolote”, tza’a tzoy (Zoque), wal oyo’ (Huasteco), x-sahum, xikin, yita cuáa (Mixteco), zikin	tooth decay, oral infections, pimples, scabies, ritual uses, incense	flowers, leaves, whole plant	[99], MEXU
63	Millerieae	*Melampodium perfoliatum* (Cav.) Kunth *	*Melampodium perfoliatum*	acahual, acahual pinto, agua traer hierba, aguadora, be’el tzukum (Tzotzil), cha’ba balan k’in (Tzeltal), k’analnich bramal, lampote cabezón, lusaco, malva, quelitillo, quesillo, sak’al wamal (Tzeltal), sakil marawiya (Tzeltal), tinaja, tinajilla, yucu yaa stiqui “hierba lengua de toro” (Mixteco)	toothache, urinating pain, earache	flowers, leaves, whole plant	[48,100], MEXU
64	Millerieae	*Tridax procumbens* L. *	*Tridax procumbens*	capitaneja, coronilla, coronita blanca, flor amarilla, guichi yaana, guixi bichupa, hierba del conejo, hierba de iguana, hierba de venado, hierba de San Juan, k’oxox wamal, madremaíz, mi najtes nagtish iet “que nace en terreno salitroso”, mi sox coual, San Juan del monte, ta’uluum, yerba San Juan del Monte	antihemorrhagic, diuretic, accelerate childbirth, headache	roots	[21,101], MEXU
65	Nassauvieae	*Acourtia cordata* (Cerv.) B.L. Turner ***	*Acourtia cordata*	árnica, hierba del zopilote		ND	[19,102], MEXU
66	Nassauvieae	*Acourtia humboldtii* (Less.) B.L. Turner ***	*Perezia adnata*	ND		ND	[103]
67	Nassauvieae	*Acourtia mexicana* (Lag. ex D. Don) H. Rob. *	*Acourtia thurberi*	Thurber’s desertpeony	medicine uses, purgative and tonic	ND	[22,104]
68	Neurolaeneae	*Calea ternifolia* Kunth *	*Calea ternifolia*	ahuapatli, amargo bola palo, amula, ch’aal wamal (Tzeltal), chichicasle, cuanasana, cuanasana con flor, garañona, garañona amarilla, guanasana, hamula, hierba del perro, hoja amargosa, jamula, jaral, jaralillo, ka tzá`jnà (Nguíiva), la oaxaqueña, la prodigiosa, monte rojo, negro, negro Juana sana, poop taam ujts, prodigiosa, rojo Juana sana, sacachichie, stetE nambú (Pame), tam huñi (Popoluca), ton ch’aj te’ (Tzeltal), tazin, tzotz te’ (Tzeltal), yerba amarga, x-tok-aban, xicin, xikin, yucocahua (Mixteco), yucu cáva “hierba amarga”, yúcu-owa (Mixteco), yucucahua, zacachichi, zacate chichi, zacate del perro, zacatechi, zacatechichi	antipyretic, diuretic, scalp conditions, bile problems, colic, diabetes, headache, stomachache, abdominal pain, liver disease, fever, flu, skin inflammation, malaria, bitter taste in the mouth, cough, vertigo, evil wind	flowers, leaves, whole plant, branches with leaves, stems	[21,105], MEXU
69	Neurolaeneae	*Calea urticifolia* (Mill.) DC. *	*Calea urticifolia*	amargo, barretero, chichiltechaca (Náhuatl), chichiquizo, chikin, chilchaca, chinche, chinchin ay (Popoluca), chinina, colmena, confiturilla, echyao dampóo “ojo negro” (Pame), hierba de paloma, hierba del barretero, hierba del perro, hierba del negro, hierba de la rabia, hoja amarga, ina nakun, jongala, K`axikin, lengua de borrego, manita de león, negrito, prodigiosa, prolijiosa, quinina, saljoás dampóo (Pame de La Palma), tacote, tacote amarillo tam ugt’s (Zapoteco), tsuleek’ ethem (Huasteco), tuwi ay/ takak tane (Zoque), x-kah xikin, x-k’ah xikin, x-tu’xikilin, x-tu’xikin, x-tuxikin, xiim bagaatxy, xicin, xikin, xikinkaax	edible, antimalarial, bile problems, weaning, stomachache, eliminate screwworms, pimples, skin wash, urinary infections, psoriasis, scabies, inducing vomiting, purgative	flowers, leaves whole plant, roots	[21,106,107], HEFA, MEXU
70	Neurolaeneae	*Neurolaena macrocephala* Sch. Bip. ex Hemsl. ***	*Neurolaena macrocephala*	cola de faisán, hoja amarga, ma ot se (Chinanteco), tzaltuwi (Chinanteco)	medicinal uses, in baths, diabetes, veterinary uses, eliminate mange in dogs	leaves	[108], MEXU
71	Neurolaeneae	*Neurolaena oaxacana* B.L. Turner ***	*Neurolaena oaxacana*	ND		ND	[109]
72	Senecioneae	*Barkleyanthus salicifolius* (Kunth) H. Rob. & Brettell *	*Senecio salignus*	asúchil (Náhuatl), asúchitl, azomiate, azumiate, azomiatl, chamizo, chamizo de hoja larga (ifaiquchac), chamizo fresco, chamizo hediondo, chijil kat (Tzeltal), chilca, copal, cuerepillo, estafiate, huerepillo, itzkaratarakua (Purépecha), jara, jara amarilla, jara brava, jara mexicana, jarilla, jarilla blanca, nuyuji “flor amarilla” (Mixteco), pescadillo, tojtin, tuxi (ita), tuyutzi, zomiate, yàg-yàaz-nguòts (Zapoteco), yedaninii (Zapoteco), yoyotl (Náhuatl)	for flavor bread, dermatological uses, pimples, cure rashes, for baths, canker sores, bile problems, colic, stomachache, flank pain, chills, erysipelas, bruises, swelling, eye inflammation, rheumatism, hangover, treat constipation, heart palpitations, sprains	flowers, leaves, inflorescence, wood, whole plant, branches with leaves, tender branches	[19,21,38,110], HEFA, MEXU,
73	Senecioneae	*Packera toluccana* (DC.) W.A. Weber & Á. Löve ***	*Senecio toluccanus*	lengua de vaca, rabanillo	medicinal and ritual uses	flowers, leaves	[19,20,111], MEXU
74	Senecioneae	*Pittocaulon bombycophole* (Bullock) H. Rob. & Brettell ***	*Pittocaulon bombycophole*	candelerillo, tlatixpatli	cough relief	bark	[112], MEXU
75	Senecioneae	*Pittocaulon filare* (McVaugh) H. Rob. & Brettell ***	*Pittocaulon filare*	maguey de arroyo		ND	[112,113], MEXU
76	Senecioneae	*Pittocaulon hintonii* H. Rob. & Brettell ***	*Pittocaulon hintonii*	ND		ND	[112]
77	Senecioneae	*Pittocaulon praecox* (Cav.) H. Rob. & Brettell ***	*Pittocaulon praecox*	candelero, candelilla, consuelda; cuerepillo, palo bobo, palo bofo, palo loco, tesapacle, tezacpatli,, tezcapatli (Náhuatl), tiramba	wound healing, rheumatic pain	leaves steams	MEXU [19,112,114]
78	Senecioneae	*Pittocaulon velatum* (Greenm.) H. Rob. & Brettell *	*Pittocaulon velatum*	candelerillo, chimpancel, consuelda, soldadura, tutáano (Mixteco), yàg-guiÊe-yél-mách (Zapoteco)	medicinal uses, heal fractures	leaves, branches, stems	[112], MEXU
79	Senecioneae	*Psacalium beamanii* H. Rob. ***	*Psacalium beamanii*	ND		ND	[115],
80	Senecioneae	*Psacalium decompositum* (A. Gray) H. Rob. & Brettell *	*Psacalium decompositum*	matariki (Guarijío), matarique, matariqui	diabetes, gastritis, lotion for sores	roots	[116], MEXU
81	Senecioneae	*Psacalium peltatum* (Kunth) Cass. ***	*Psacalium peltatum*	matarique, sombrerete, sombrilla	diabetes, kidney disease, rheumatism	roots	MEXU
82	Senecioneae	*Psacalium radulifolium* (Kunth) H. Rob. & Brettell ***	*Psacalium radulifolium*	matarique		ND	[117], MEXU
83	Senecioneae	*Robinsonecio gerberifolius* (Sch. Bip. ex Hemsl.) T.M. Barkley & Janovec *	*Robinsonecio gerberifolius*	ND		ND	[118]
84	Senecioneae	*Roldana angulifolia* (DC.) H. Rob. & Brettell *	*Roldana angulifolia*	mano de león, pata de león, sombra	medicinal uses, heal wounds, to cure varicose veins	leaves	[20,119], MEXU
85	Senecioneae	*Roldana barba-johannis* (DC.) H. Rob. & Brettell *	*Roldana barba-johannis*	cuaresmilla, barba de San Juan de Dios, gordolobo	the sap is used as substitute for drinking water	sap	[20,114,120], MEXU
86	Senecioneae	*Roldana candicans* (Née) Villaseñor, S. Valencia & Coombes ***	*Roldana lineolata*	arbusto, hediondilla		ND	[20,121,122], MEXU
87	Senecioneae	*Senecio mairetianus* DC. *	*Senecio mairetianus*	ND		ND	[123]
88	Senecioneae	*Senecio polypodioides* (Greene) T. Durand & B.D. Jacks. ***	*Senecio polypodioides*	guìz-crÛz (Zapoteco), zhìn-blág-l-guìt (Zapoteco)		ND	[124], MEXU
89	Tageteae	*Chrysactinia mexicana* A. Gray *	*Chrysactinia mexicana*	damiana, falsa damiana, hierba de San Nicolás, kalanca, mariola, romerillo, San Nicolás	medicinal uses, postpartum baths, diarrhea and indigestion, gastrointestinal problems	ND	[19,125], MEXU
90	Tageteae	*Dyssodia tagetiflora* Lag. ***	*Dyssodia tagetiflora*	amocillo amarillo, árnica, cabezona, cilantrillo, cinco llagas, flor de muerto, tsju ka`turìndú (Nguíiva)	edible, medicinal uses	whole plant	[126], MEXU
91	Tageteae	*Tagetes erecta* L. *	*Tagetes erecta*	aceitilla, alpushum (Totonaco), capusham, cempasúchil (Náhuatl), cempasúchil cimarrón, cempasúchil de campo, cempasúchil doble, cempasúchil hombre, cempasúchil macho, cempasúchil mujer, cempasúchil relleno, cempasúchitl, cempazuchitl silvestre, cempoal, cempoalsúchil, cempoalxóchitl (Náhuatl), ch’ulelal nichim (Tzotzil), chili pepper zempasuchil, churiptzutzuki, cinco llagas, cocosacle, discoque, espinosilla, flor de muerto, kavinzeran, kelem nichim (Tzotzil), muertito, nakastapal (Náhuatl) nulibé,piixxch (Mixe), rosa de muerto, santorum wits (Huasteco), tsuts mooya (Popoluca), xpuch, yita cuáa (Mixteco)	abortifacient, postpartum baths, bronchitis, colic, bile problems, diarrhea, headache, stomachache, toothache, earache, chest pain, dental pain, muscle ache, indigestion, stomach stimulant, fever, skin infections, inflammation, wash wounds, malaria, urinary infections, dizziness, nervousness, venomous animal bites, colds, skin rashes, cough, vomiting, warts, nematicide, pesticide, evil eye, fright, evil wind	branches, flowers, heads, leaves, roots, whole plant	[19,127], MEXU
92	Tageteae	*Tagetes filifolia* Lag. *	*Tagetes filifolia*	anís, anís cimarrón, anís macho, anís montés, anís de estrella, anís de perro, anís del campo, anisato, anisillo, anix wamal (Tzeltal), camino de aguacatillo, cardo lechero, ehtiil anís (Huasteco), estrella pok (Zoque), guiÊe-nìzh (Zapoteco), guìzh-nìzh (Zapoteco), hierba anís, hierba del chapulín, inajo antivo (Tzotzil), kulanto jomol (Tzotzil), kulantu wamal (Tzeltal), kulentu jos (Tzeltal), kulentu wamal (Tzeltal), la ye (Zapoteco), majbenal vomol, manzanilla, mantzaniya wamal (Tzeltal), menta, nantzin wamal (Tzeltal), pericón, putsut, putzuti, santóä (Pame), tacuaú mishi (Mixteco), tlalahuacate, tsäaptsip (Mixe), tuyhxiids (Mixe), tzail mantzania (Tzeltal), tzitz ak (Tzeltal), tzitz jomol (Tzotzil), ya ta yade (Cuicateco), yi she anís (Zapoteco)	flavoring in beverage, analgesic, antiflu baths, colic, congestion in children, bile problems (anger), diarrhea, stomachache, heart ache, chills, expel gas, fever, gastrointestinal diseases, suffocation, whooping cough, anthelmintic	branches, leaves, roots, whole plant	[19,21,50], MEXU
93	Tageteae	*Tagetes lucida* Cav. *	*Tagetes lucida*	anís, basigó (Tarahuamara), campanario, cedrón, flor de perico, flor de Santa María, gi huats (Mixe), guiahuajsa’ac (Zapoteco), gui’uass (Zapoteco), guiÊe-dzÛu (Zapoteco), guiêe-dzûu (Zapoteco), guìz-guiÊe-dzÛu (Zapoteco), [guìz-]guiêe-dzûu (Zapoteco), hierba anís, hierbanís, hierba de San Juan, hierba santa, ih’yaquesho (Zapoteco), ita shini “flor de cabezuela”, iya ke shu (Zapoteco), kotzok ay (Zoque), kukurumin (Purépecha), kuruhkuntzutzuki (Purépecha), jericón, malvarisco, mantzania wamal (Tzeltal), mantzaniya wamal (Tzeltal), perico, pericón, pericón amarillo, pimente wamal, pirican, re’e mba mika, re’e xe, Santa María, sansiwre wamal (Tzeltal), sallaucle, sibarín, tsuktsukurnkumo (Purépecha), tz’i’ momol (Tzoltzil), tzitz (Tzoltzil), tzitz ak (Tzeltal), tzitz ak wamal (Tzeltal), tzitz jomol (Tzotzil), tzitz wamal (Tzeltal), tzitz zak (Tzeltal), tzitz zak wamal (Tzeltal), tzitzimuk (Zoque), yahutli, yerba anís, yerba nis (Tarahuamara), yerbanís, yitapericoo (Mixteco), yúcu-perico (Mixteco), yuvat (Tepehuano), zacayautle, zacayautli	calm crying children, gas in stomach, analgesic, postpartum baths, colic, bile problems (anger), dysentery, diarrhea, digestive, headache, heartache, ctomach ache, abdominal pain, indigestion, fever, puerperal fever, gastrointestinal disorders, gynecological uses, flu, inflammation, oxytocic, hangover, colds, cough, antihelmintic, evail air, fright, tonic use	aerial parts, flowers, leaves; whole plant	[128], HEFA, MEXU
94	Vernonieae	*Vernonia liatroides* DC.***	*Vernonia liatroides*	moradilla, vara de San Miguel, varita, xeljoas’ (Pame)	wash wounds and pimples, eviul eye	whole plant	[73,129], EBUM, MEXU

* Native, ** introduced, and *** endemic species.

### 3.2. Phytochemistry

This review presents 494 chemical constituents isolated or identified from Asteraceae species from Mexico. The secondary metabolites were classified according to their biosynthetic origins as follows: 25 alkaloids (**12**–**36**), 55 flavonoids (**38**–**93**), 67 phenolics and aromatic compounds (**94**–**160**), 326 terpenoids (**1**, **159**–**483**), and 21 miscellaneous compounds that include alkanes, enamide, glycosides, polyene, lactones, and alkamides (**484**–**504**).

#### 3.2.1. Alkaloids

Alkaloids are compounds with acid-base properties that contain nitrogen in their structure and occur as monomeric and dimeric forms, salts, zwitterions, and *N*-oxides, and can be found conjugated with residues of specialized metabolites from other biosynthetic origins [130,131,132]. Alkaloids are a complex group of secondary metabolites whose biosynthesis begins from amino acids, such L-histidine, L-lysine, L-ornithine, L-threonine, L-tryptophan, L-phenylalanine and L-tyrosine, and aromatic acids, such as anthranilic and nicotinic acids, to yield a great variety of backbones: acridine, b-carbolines, indolizidine, pyrrolizidine, piperidine, quinazoline, quinolizidine, isoquinoline and indolizidine, among others [28,133]. The main kind of alkaloids isolated from the Asteraceae family in Mexico are pyrrolizidine-type alkaloids (PA, **12**–**36**) (Figure 2), which are relevant to the agronomic and food industries because they are toxic to mammals, including humans [134,135]. These compounds play a defense role in Asteraceae plants, protecting them against herbivory, and can also be sequestered by insects for defense against predators [136,137,138,139]. From the aerial parts of the medicinal plant *Ageratum houstonianum* (Mill) (Asteraceae, tribe Eupatorieae), also known by the natives from Tlanchinol, Hidalgo, Mexico as “micashihuil” (Nahuatl), four pyrrolizidine alkaloids were isolated and their structures determined as lycopsamine (**12**), retrohoustine (**13**), heliohoustine (**14**), and isoretrohoustine (**15**) [67]. Supinidine triviridiflorate (**16**) and supinidine diviridiflorate (**17**) were identified from the roots of *Chromolaena pulchella* [72]. The PA **18–33** were identified in all the species that comprise the *Pittocaulon* genus (Figure 2). For example, in *P. bombycophole*, *P. filare* and *P. hintonii*, 7-angeloylturneforcidine (**18**), senkirkine (**27**) and acetylpetasitenine (**33**) were obtained from stems and roots [112]. Compounds **23** and **24** were identified in *Senecio mairetianus* [123], and the PA **34**–**36** were isolated from a MeOH-soluble extract of roots of *Senecio polypodioides* [124].

#### 3.2.2. Flavonoids

Flavonoids are specialized metabolites derived from the phenylpropanoid and shikimate pathways. Biosynthesis begins using cinnamoyl-CoA or *p*-coumaroyl-CoA as starter groups, and they subsequently react with three malonyl-CoA molecules *via* condensation/cyclization reactions to generate the first key intermediates in the flavonoid biosynthesis pathway [28,140]. The isolated flavonoids from Mexican species belonging to the Asteraceae family suggest that the principal intermediate on this pathway is through naringenin (**37**); after this, a series of enzyme reactions can take place to produce diverse flavonoids [140]. Flavonoids can occur in free form or glycosylated, as mono-, di-, or oligomers, or they can occur covalently bonded to specialized metabolites from other biosynthesis pathways to produce hybrid molecules or adducts [141,142,143]. Diversity of flavonoids arises in the degree of oxidation of the base core and its later acylation, methoxylation, prenylation, and additional cyclizations; likewise, they can also be sulfated [144,145]. Flavonoid glycosides can occur as *C*- or *O*-glycosides, while the specialized metabolites containing a residue of flavonoid are conjugated to alkaloids or terpenoids [141,142]. These compounds are produced in plants as inhibitors of seed germination, pigments, signaling molecules to establish interkingdom communication, in response to pathogen infections, for the establishment of plant–rhizosphere microbiota symbiosis relationships, as UV-B protectants, and to regulate auxin transport [146,147,148]. The identified flavonoids isolated from plants of the Asteraceae family with distribution in Mexico included five flavanones (**37**–**41**), eleven flavones (**42**–**52**), two flavone glycosides (**53**–**54**), eighteen flavonols (**55**–**72**), eighteen flavonol glycosides (**73**–**90**), and one isoflavone (**91**) (Figure 3). The genus with the largest number of flavonoids is *Dyssodia*. The flavanones included naringenin (**37**), pinostrobin (**38**), naringenin-4′,7-dimethylether (**39**), 7,3′-di-*O*-methyleriodictyol (**40**) and 7,4′-di-*O*-methyleriodictyol (**41**). They have been described on aerial parts of *Baccharis conferta* [149,150], *Pittocaulon velatum* [151], *Laennecia filaginoides* (as *Conyza filaginoides*) [44], and *Heterotheca inuloides* [152,153,154,155]. The flavones were described from *Artemisia ludoviciana* subsp. *mexicana* (**42**–**43**) [156], *Baccharis conferta* (**44**–**48**) [40,149,150,157], *Calea* species (**47**, **49**–**50**) [158], *Brickellia paniculata* (**51**) [69] and *Zinnia peruviana* (**52**) [98]. The flavone *C*-glycosides derived from Mexican Asteraceae plants occur in *Baccharis conferta* as C-6 and C-8 diglucosides (vicenin 2, **53**) [150], and from *Cirsium subcoriaceum*, pectolinarin (**54**) was isolated [159]. The flavonols have been reported in *Dyssodia tagetiflora* (**55**–**56**) [160], *Heterotheca inuloides* (**55**, **57**, **58**) [152,153,154], *Calea zacatechichi* (**55**, **59**–**61**) [158], *Pseudognaphalium liebmanii* (reported as *Gnaphalium liebmanii*) (**62**–**63**) [84], *Laennecia schiedeana* (**64**–**65**) [44], *Baccharis conferta* (**61**, **66**–**70**) [149,150,157], *Pittocaulon velatum* (**61**) [151], *Senecio mairetianus* (**55**) [123], and *Tagetes* species (**55**, **71**, **72**) [31,127]. All the flavonols isolated from Asteraceae were contained in methanol-soluble extracts of aerial parts of these plants. Flavonol glycosides include astragalin (**73**) [45,56,161], rutin (**74**) [124,140,141], hyperoside (**75**) [126,160], nicotiflorin (**76**) [44,68,161], kaempferol 3-O-(6″-*O*-*E*-caffeoyl)-β-D-galactopyranoside (**77**) [161], isorhamnetin 3-*O*-(6″-*O*-*E*-caffeoyl)-β-D-galactopyranoside (**78**) quercetin 3-*O*-(6″-*O*-*E*-caffeoyl)-*β*-D-glucoopyranoside (**79**), isoquercitrin (**80**) and narcissin (**81**) [161], isorhamnetin-3*β*-D-(*O*-acetyl) glucoside (**82**), quercetin-3-*O*-glucose-6″-acetate (**83**) [123], avicularin (**84**) and avicularin acetate (**85**) [126,160], and kaempferitrin (**86**) [72,162]. These flavonol glycosides (**75**–**81**) were identified in *Laennecia filaginoides (as Conyza filaginoides)* [45,162]. Some of them showed biological activity such as antiprotozoals and phytoprotectors. The flavonol glycosides described possess the sugar moieties attached at different positions, and they were identified in *Dyssodia tagetiflora* (**87**, **88**) [126] and *Ageratina grandifolia* (**89**, **90**) [62]. The isoflavone **91** was obtained from a methanol-soluble extract of the flowers of *Tagetes erecta* [127]. Some of these compounds have displayed antimicrobial, antiprotozoal, cytotoxic, and gastroprotective activities, which are discussed below.

#### 3.2.3. Phenolics and Aromatic Compounds

The phenolics and aromatic compounds are synthetized in the shikimate pathway by several non-oxidative and oxidative reactions to produce aromatic acids [163]. The aromatic compounds cover twenty-one benzoic acid derivatives (**92**–**112**), seven chlorogenic acid derivatives (**113**–**119**), one coumarate (**120**), twelve coumarin derivatives (**121**–**131**, **136**), eleven chromenes (**132**–**135**, **137**–**143**), three benzofurans (**144**–**146**), four heteroaromatic compounds (**147**–**150**), three pyrogallol derivatives (**151**–**153**), two catechol derivatives (**154**–**155**), and two phenylethanoids (**156**–**157**) in Asteraceae species (Figure 4) [53]. The benzoic acid derivatives **93**, **94**, **96**, **98**–**106** and **112** occurred in *Brickellia veronicifolia* [70,164,165]; compounds **95**, **101**, **103**, **104**, and **107**–**111** in *Ageratina deltoideia* [58]; while compounds **92** and **97** occurred in *Bidens odorata* and *Flourensia cernua*, respectively [53,88]. The genus with the largest number of aromatic compounds is *Ageratina* [59,60,61,62,63,64,65]. Chlorogenic acid derivatives have been described from *Tagetes filifolia* (**113**–**114**) [50], *Laennecia schiedeana* (**113**, **115**, **116**) [44], *Baccharis conferta* (**115**) [150], *Calea urticifolia* (**116**–**119**) [166], and *Bidens odorata* (**120**) [53]. Coumarins have been isolated from organic extracts of diverse polarities. For example, from *Tagetes lucida*, several research groups obtained coumarins from organic extracts of different polarities. Céspedes et al. obtained seven coumarins (**121**–**127**) from a methanolic extract [30]; Estrada-Soto et al. isolated the coumarins **123**, **125** and **128** from an ethanolic extract [167]; and Monterrosas-Brisson et al. isolated five of them (**123**, **126**, **128**–**130**) from a hexane extract [128]. From *Hetheroteca inuloides,* 7-isoprenyloxycoumarin (**129**) was isolated [168,169]; from *Ageratina grandifolia,* the coumarines **131**–**135** [62]; and from *Acourtia mexicana* (reported as *Acourtia thurberi*), the coumarin **136** [104]. Concerning the chromene group, two of the compounds (**138** and **139**) were isolated from *Calea ternifolia* [105], and three from *Roldana barba-johannis* (**141**–**143**) [120,170]. The compounds **137**, **139**, and **143** were from *Ageratina* species [61,63], and **140** was from *Gonzalezia decurrens* (reported as *Viguiera decurrens)* [90]. The Eupatorie species *Ageratina pichinchensis* (also registered as *Eupatorium aschenbornianum*) produced the benzofurans **144**–**146** [64,171]. The thiophenes are derivatives from oleic acid via acetylene intermediates. These compounds can be identified in specific parts of the Asteraceae plants. The thiophene **147** was obtained from the fresh root bark of *Chrysactinia mexicana* [125]; and from the aerial parts of *Dyssodia tagetiflora*, the thiophenes **148** and **149** were obtained, and from its roots **147**, **149**, and **150** [160]. Pyrogallol derivatives were isolated from polar and middle-polar extracts of *Brickellia veronicifolia* (**151**) [164] and *Pittocaulon filare* (**152**–**153**) [113], while the catechol derivatives **154–155** were obtained from *Cosmos pringlei* [54].

Other kinds of aromatic compounds are the phenylethanoids from *Psacalium beamanii* (**156**) and *Pittocaulon filare* (**157**) [113,115], and a furopyran (**158**) from *Hofmeisteria schafferi* [75,172]. Beyond the antioxidant capability associated with the phenolics, which is commonly applied in preventive/protector effects of chronic degenerative diseases or physiological processes that trigger these, such as cancer and inflammation, hepatic and renal damage, hypertension and diabetes, research is still necessary into their molecular action mechanisms, their toxicity, and their assaying in models targeting the central nervous system.

#### 3.2.4. Terpenoids

Terpenoids are the most extensive and structurally diverse class of natural products isolated from plants belonging to the Asteraceae family. These secondary metabolites are derived from the five-carbon precursors isopentenyl diphosphate (IPP) and dimethylallyl diphosphate (DMAPP), which are biosynthesized through the mevalonate (MVA) and methylerythritol phosphate (MEP) pathways. Terpenoids confer distinctive chemical and ecological properties to Asteraceae plants that allow them to survive in their habitats, such as odor-mediated attraction of pollinators, allelopathy and defense mechanisms, among others [173,174]. These ecological functions of specialized metabolites from Asteraceae led to the development of agrochemicals such as insecticides and bioactive principles with pharmaceutical relevance, which will be discussed in detail later [175]. From Mexican Asteraceae plants, the following subtypes have been described: mono- (C10), sesqui- (C15), di- (C20), and triterpenoids (C30). Of them, the sesquiterpenes are the most representative, and the sesquiterpenes often possesses a 2-methylenecyclopentan-1-one moiety in their structure, so they are called sesquiterpene lactones, which in turn also have a subclassification according to the carbon backbone, the position of lactonization, and the occurrence and geometry of their double bonds. Each terpenoid subtype is discussed in detail below [176].

##### Monoterpenoids

Monoterpenoids are characterized by carbon skeletons containing 10 carbon atoms and are produced from geranyl diphosphate (GPP) through a series of enzymatic reactions, including dephosphorylation, cyclizations, oxidations, and acylations, among others, until giving the final biosynthesis compounds for each plant species [14,76,172]. The principal monoterpenoids identified in Mexican Asteraceae species include 28 thymol derivatives (**159**–**161** and **165**–**190**), four of menthane type (**161**–**163** and **191**), and *β*-pinene (**164**) (Figure 5). The *Ageratina* and *Hofmeisteria* genera represent the main source of thymol derivatives, with eleven compounds (**165**–**175**) from *Ageratina* [55,57,60] and ten compounds (**160**, **181**–**188**) from *Hofmeisteria* [76,177] (Figure 5). *Calea urticifolia* produces 3-methyl-4-isopropylphenol (**159**) and thymol (**160**) [166]. The menthane glucosides **189** and **190** were isolated from a methanol extract of *Ageratina glabrata* [61], and the glucoside **191** was described in *Roldana angulifolia* [119]. The menthanes **161**–**163** and *β*-pinene (**164**) were identified by GC-MS in the essential oil from *Dyssodia tagetiflora* [160]. Thymol derivatives have been reported to exhibit antibacterial, antidiarrheal, antinociceptive, antiprotozoal and phytotoxic activities [56,76,172,177]. Therefore, additional studies are necessary to continue exploring their agrochemical and pharmacological potential, as well as the molecular mechanisms that trigger these effects.

##### Sesquiterpenoids

The sesquiterpenes and sesquiterpene lactones are among the most abundant specialized metabolites found in the Asteraceae family. These compounds are biosynthesized through the MVA pathway, in which IPP and DMAPP are condensed to produce farnesyl diphosphate (FPP), the principal precursor of sesquiterpenes [28,173]. In this review, the terpenoids constituted by 15 carbon atoms were grouped according to their frameworks. These sesquiterpenes can also be grouped as cyclic and acyclic sesquiterpenes; nevertheless, the literature search revealed that only cyclic sesquiterpenes have been reported as chemically relevant. The principal genera reported as sources of sesquiterpenes are *Pittocaulon*, *Senecio*, *Melampodium*, *Psacalium*, and *Heterotheca*.

Germacrenes **192**–**193** had been identified in essential oil by HPLC and CG-MS on *Brickellia veronicifolia* [178] and *Melampodium divaricatum* [179]. The germacranolides (**194**–**225**) and the furanoeremophilanes (**250**–**281**) are groups with a greater number of compounds, and most of them were isolated from nonpolar extracts (hexane and dichloromethane) (Figure 6).

The germacranolides constitute a particular type of sesquiterpene lactones due to the number of congeners that have been described. They can be subclassified according to the configuration (geometry) of the pair of double bonds at the ten membered ring as germacranolides (*Z*, *Z*), germacrolides (*E*, *E*), heliangolides (*E*, *Z*) and melampolides (*Z*, *E*) (Figure 7) [179]. In this review, we have grouped them following this rule. The germacranolides have been obtained from *Calea* species (**194**–**198**) [105,106,107,158], *Cosmos pringlei* (**199**–**201**) [54] and *Neurolaena* species (**202–210**) [108,109]. The germacrolides have been described in *Ageratina deltoidea* (**211**–**212**) [58], *A. vernalis* (**211**, **213** and **214**) [180], *Ambrosia confertiflora* (**215**) [85] and *Vernonia liatroides* (**216**–**217**) [129]. The heliangolides occurred in *Decachaeta incompta* (**218**–**221**) [73,181,182], *Mikania micrantha* (**222**–**223**) [78], and *Tithonia diversifolia* (**224**) [95,183], and the furanoheliangolides **225**–**227** were described in *Neurolaena oaxacana* and *N. macrocephala* [108,109,184]. The melampolides were derived from plants of *Melampodium perfoliatum* (**228**–**235**) [100], and **236**–**242** from *Mikania* species collected in Michoacan, Mexico, while the lactones **236**–**240** were isolated from populations collected in Guerrero and Oaxaca states, in Mexico [78].

Eudesmanes were isolated from different genera and species such as *Ageratina pichinchensis* (**243**) [64], *Brickellia veronicifolia* (**243**) [178], *Machaeranthera tanacetifolia* (**244**–**245**) [46], *Melampodium divaricatum* (**246**) [99], *Verbesina persicifolia* (**247**) [96], *Senecio polypodiodes* (**248**) [124], and *Pittocaulon filare* (**249**–**251**) (Figure 8) [113].

Four species had been identified as producers of the eudesmanolides. The regioisomers santamarine (**252**) and reynosin (**253**) occur in *Ambrosia confertiflora* [85], while arglanin (**254**) was obtained from an *Artemisia ludoviciana* subsp. *mexicana* infusion [156]; farinosin (**255**) and hydroxy-*bis*-dihydrofarinosin (**256**) were obtained from an acetone-soluble extract of *Encelia farinosa* [87,185], 9-deacetylmeleucanthin (**257**) from *Melampodium perfoliatum* [100], and ivalin (**258**) from *Decachaeta peroronata* (Figure 9) [186].

The eremophilane-type sesquiterpenes are representative of the genera *Robinsonecio* and *Roldana*. Four of them were described in *Robinsonecio gerberifolius* (**259**–**262**) [118], and one glucoside (**263**) from *Roldana angulifolia* (Figure 10) [119].

Most of the furanoeremophilanes were obtained from roots of the plants of genera: *Pittocaulon* (**264–268**) [113,151], and the cacalol derivatives from the genera *Psacalium* (**269**–**284**) [115,117,187,188,189,190], *Robinsonecio* (**285**) [118], *Roldana* (**286**–**292**) [120] and *Senecio* (**292**–**295**) [111,122]. Rearranged eremophilanes radulifolins A-B (**296**–**297**), and the 11-epimer (**298**) of **296** were obtained from roots of *Psacalium radulifolium* (Figure 11) [117].

The species with eremophilanolides have been reported for the genera *Roldana* (**299**–**301**) [121,170] and *Senecio* (**299**–**300**, **302**–**306**) [111,122,123]. Two *seco*-eremophiladiolides (**307**–**308**) and four eremophilane glucosides (**309**–**312**) were isolated from a methanol extract of *Pittocaulon velatum* (Figure 12) [151].

Most of the cadalenes (**313–324**) have been isolated from acetone extracts of aerial parts or flowers of *Heterotheca inuloides* [43,152,153,168,169,191,192,193], and only two of them from roots of *Stevia suaveolens* (reported as *S. nepetifolia)* (**325**, **326**) [194]. The 2,9-*seco*-cadinanes (**327**, **328**) were isolated by Egas et al. from *H. inuloides* (Figure 13) [152].

The guaianes identified from Asteraceae plants include chamazulene (**329**) from *Brickellia veronicifolia* [164], flourensadiol (**330**) from *Flourensia cernua* [88], and aromandendrene (**331**) and *γ*-patchoulene (**332**) from *Melampodium divaricatum* [99]. The guaianolides leucodin (**333**) and its epimer achillin (**334**) were obtained from *Achillea millefolium* extract [35,36], and estafiatin (**335**) from *Artemisia ludoviciana* subsp. *mexicana* [38]. From *Cosmos pringlei*, the lactones **335**–**339** [54], and from *Mikania micrantha*, the **340**–**342** ones [78]. Xerantholide (**342**) has been reported from *Decachaeta perornata* [186]. The ambrosanolides are a kind of pseudoguaianolide. These were isolated from *Parthenium hysterophorus* (**343**) [73], and cumanin (**344**) from *Ambrosia psilostachya* [86]. Helenalin (**1**) was the only helenanolide (10-*a*-methyl pseudoguanianolide) identified in Mexican Asteraceae species. It was isolated from the methanol extract of aerial parts of *Helenium mexicanum* (Figure 14) [86].

The elemenes **345**–**347** were identified in the essential oil from the aerial parts of *Melampodium divaricatum* through GC-MS analysis [99]. The elemenolide (**348**) was isolated from the hexane extract of *Ageratina deltoidea* [57], and the compounds **349**–**357** from *Zinnia* species [97,98,195]. Lactone **358** occurs in *Mikania micrantha* (Figure 15) [78].

Longipinenes **359**–**370** are specialized metabolites distinctive of *Stevia* species. Many of them were obtained from their roots (Figure 16) [80,81,82,83,194].

Oplopanes **371**–**377** were derived from *Pittocaulon filare* [113] and the rest from *Robinsonecio gerberifolius* (**368**–**379**) (Figure 17) [118]. The bisabolenes were isolated from the roots of *Iostephane heterophylla* (**380**–**386**) [92,196], from the aerial parts of *Melampodium divaricatum* (**387**–**388**) [99], and from the roots of *Stevia tomentosa* (**389**–**390**) [83] and *Stevia suaveolens* (reported as *S. nepetifolia)* (**390**) (Figure 18) [194].

Perezone (**391**), a *p*-quinone sesquiterpenoid, was obtained from the roots of *Acourtia* species [102,104]. The cedrene (**392**) was identified in the hexane extract of *Heterotheca inuloides* by GC-MS [154]. Cedranolides are tricyclic sesquiterpenes isolated from roots of *Acourtia thurberi*, *alpha* (**393**) and *beta*-pipitzol (**394**), which are cyclization products of perezone (**391**) (Figure 19) [104].

β-caryophyllene (**395**) has been described in *Brickellia veronicifolia* [164], *Melampodium divaricatum* [99], *Barkleyanthus salicifolius* (reported as *Senecio salignus)* [110], and *Stevia monardifolia* [80], while caryophyllene oxide (**396**) was identified in *Laennecia filaginoides* (as *Conyza filaginoides*) [45], *Barkleyanthus salicifolius* (as *Senecio salignus*) [110], *Stevia tomentosa* [83], *Xanthocephalum gymnospermoides* [47] and *Heterotheca inuloides* [191]. Pérez-Vásquez et al., 2021 identified α-humulene (**397**) and α-cubebene (**398**) as major constituents of the essential oil of *Melampodium divaricatum* (Figure 20) [99].

Only a caleprunane (**399**) sesquiterpene type was isolated from a dichloromethane extract of *Baccharis conferta* [40]. Caryolan-1,9-β-diol (**400**) was obtained from the aerial parts of *Heterotheca inuloides* [154], and senecrassidiol (**401**) from the roots of *Roldana angulifolia* [119]. The oppositanes **402**–**403** were obtained from the roots and stems of *Pittocaulon filare* (Figure 20) [113].

##### Diterpenoids

Diterpenoids are biosynthesized from IPP and DMAPP, which are derived from the MVA and MEP pathways, respectively. Their structural diversity arises from sequential biosynthetic steps: first, the carbon skeleton is constructed by diterpene synthases. Next, the skeleton is oxidized by cytochrome P450 monooxygenases or dioxygenases. Finally, structural modifications are introduced in different reactions such as transferases and isomerases [197]. The diterpenes would be acyclic and cyclic structures [80]. Therefore, a common acyclic diterpene is phytol (**404**), isolated from *Stevia monardifolia* [80]. Monocyclic diterpenes from Asteraceae species include centipedic acid (**405**) and conyzaleucolide A (**406**), isolated from *Laennecia schiedeana* (Figure 21) [44]. The representative bicyclic diterpenoids from Asteraceae in this review contain carbon backbones of labdanes (**407**–**426**), *neo*-clerodanes (**427**–**439**), and *ent*-kaurenes (**440**–**451**) (Figure 22, Figure 23 and Figure 24).

The labdanes were principally obtained from the *Ageratina* (**407**–**410**) [63], *Brickellia* (**411**) [198], and *Chromoleana* (**412**–**413**) genera [72]. From *Gymnosperma glutinosum* leaves, the *ent*-labdanes have been obtained **414–415** [199]. From *Xanthocephalum gymnospermoides* and *Stevia pubescens*, a series of labdanes bearing a pyrane or tetrahydrofuran ring moieties in their structures (**416**–**426**) was isolated (Figure 22) [47,82].

The *neo*-clerodane diterpenoids occurred in three species: *Baccharis conferta* (**427**–**429**) [40,157], *Chromolaena pulchella* (**430**–**434**) [72], and *Laennecia schiedeana* (**435**–**437**) [44]; while in *Gymnosperma glutinosum,* diterpenoids belonging to the *ent-neo*-clerodanes series (**438**–**439)** were described (Figure 23) [42,199].

The *ent*-kaurane diterpenoids were reported from *Iostephane heterophylla* (**440**–**443**) [92], followed by *Ageratina deltoidea* (**440**–**444**) [58], *Gonzalezia decurrens* (reported as *Viguiera decurrens*) (**444**–**445**) [90], *Machaeranthera tanacetifolia* (**446**) [46], and *Melampodium perfoliatum* (**447**) [100]. The *ent*-kaurane glucosides **449**–**450** occur in *Ageratina cylindrica* [55], and *Melampodium* species (**448**, **451)** (Figure 24) [99,100].

##### Triterpenoids and Phytosterols

Triterpenoids are a large group of metabolites isolated from different living organisms. Those described from Mexican Asteraceae species include oleananes (**452**–**465**), dammaranes (**466**–**467**), cycloartanes (**468**–**470**), taraxerols (**471**–**472**), lupanes (**473**–**476**), and one hopane (**477**), as well as phytosterols (**478**–**483**), which are considered modified triterpenes (Figure 25 and Figure 26).

The oleananes have been described in *Baccharis conferta* (**452**–**454**) [40,149], *Laennecia filaginoides* (as *Conyza filaginoides)* (**454**–**455**) [45,161], *Heterotheca inuloides* (**456**–**458**) [152], *Laennecia schiedeana* (**459**–**460**) [44], *Stevia* species (**459**–**462**) [79,194], and from *Viguiera* species, the glycoside derivatives (**452**, **463**–**465**) were reported (Figure 25) [90].

From *Stevia tomentosa,* two dammaranes were reported: (20*S*)-dammar-13(17),24-diene-3*β*-yl acetate (**466**) and (20*S*)-dammara-13(17),24-dienol (**467**) [83]. From *Parthenium argentatum*, there were three cycloartanes: argentatins A (**468**), B (**469**), and D (**470**) [94,200]. Taraxerol (**471**) was isolated from *Ageratina pichinchensis* [64], and taraxasterol acetate (**472**) from *Brickellia veronicifolia* [70]. Lupanes, such as lupeol acetate (**473**), were isolated from *Stevia tomentosa* [83], lupeol (**474**) from *Mikania micrantha* [78]; 3β,16β-dihydroxylupeol 3-myristate (**475**) and 3β,16β-dihydroxylupeol 3-isopalmitate (**476**) were isolated from *Roldana barba-johannis* [170]. The hopane-type triterpene named zeorine (**477**) was derived from *Ageratina vernalis* [180].

*β*-Sitosterol (**478**), stigmasterol (**479**), and α-spinasterol (**480**) are ubiquitous and have been found in *Ageratina pichinchensis* (also reported as *Eupatorium aschenbornianum*) and *Laennecia schiedeana* [44,64,171], while their glucoside derivatives *α*-spinasteryl β-D-glucopyranoside (**481**), (3β)-stigmast-5-en-3-yl β-D-glucopyranoside (**482**), and *β*-sitosteryl-3-*O*-β-D-glucopyranoside (**483**) have been described in *Heterotheca inuloides*, *Brickellia veronicifolia*, and *Gonzalezia decurrens* (reported as *Viguiera decurrens*) (Figure 26) [68,90,152,154,168].

##### Miscellaneous

The specialized metabolites described in Asteraceae plants with a reduced number of structures or that possess a different biosynthesis to those discussed above were included in this section and considered to be the glycosides **484**–**485**, the alkane **486**, the polyene **487**, and the enamide **488**. The glycoside metabolites were isolated from *Ageratina cylindrica* (**484**) [55,56]. *Gymnosperma glutinosum* produces D-glycero-D-galactoheptitol (**485**) and hentriacontane (**486**) [199]. *Tridax procumbens* produces the oxylipin (3*S*)-16,17-didehydrofalcarinol (**487**) [101,201], and *Melampodium divaricatum* produces the enamide (**488**) [99]. Some reports have described the alkamides **489**–**497** in *Heliopsis longipes* [202,203,204,205,206,207,208,209,210], and the lactones **498**–**504** occurred in *Flourensia cernua* (Figure 27) [88].

### 3.3. Pharmacological Properties

In a second instance, this work describes the biological properties of different compounds identified in Mexican Asteraceae species over the last 30 years. These include their analgesic, antidiabetic, anti-inflammatory, antimicrobial, antiprotozoal, cytotoxic, gastroprotective, neuroprotective, and vasoactive effects, among others. This review summarizes available evidence on the biological activities of the plants with edible and ethnomedicinal uses, their specialized metabolites, and their potential pharmacological and agrochemical applications (Figure 28).

#### 3.3.1. Analgesic and/or Antinociceptive

Pain is defined as an aversive sensory and emotional experience, usually caused by actual or potential tissue damage [211]. Nociception, the physiological process responsible for detecting and transmitting stimuli to the central nervous system, giving rise to pain perception, can be classified as physiological, inflammatory, and neuropathic pain, and can present as acute or chronic [212]. The prolonged use of conventional analgesics induces several adverse effects in humans, so there is growing interest in the search for natural products as a source of agents with analgesic and antinociceptive activity. Phytochemical studies have also identified specific metabolites responsible for this pharmacological activity. Several Mexican Asteraceae species have shown significant activity in two experimental pain models: writhing or formalin. Among them, an ethanolic extract of *Bidens odorata* showed an analgesic dose-dependent response in rats in acetic acid-induced writhing assays, reaching its greatest effect at 200 mg/kg of ethanolic extract of *Bidens odorata*, comparable to that obtained with 100 mg/kg naproxen [52]. Also, similar antinociceptive effects were observed in the writhing assay with the extract of *Brickellia veronicifolia*. As a result of that study, four metabolites were isolated by bio-guided fractionation. The benzyl-2,6-dimethoxybenzoate was the most active metabolite, which at 100 mg/kg reduced abdominal constrictions by 45% [70]. *Heliopsis longipes*, traditionally used to relieve toothache, yielded four fractions with analgesic activity, identifying affinin (**489**) as the main bioactive compound, and demonstrating that its analgesic effect is related to the GABA system [91,205]. Alternatively, *Artemisia ludoviciana* subsp. *mexicana* essential oil significantly reduced nociceptive responses in both phases, indicating activity against neurogenic and inflammatory pain; interestingly, the reversal of this effect by naloxone suggests the involvement of the opioid system [213]. Likewise, *Tagetes lucida* essential oil and β-caryophyllene, its main constituent, exhibited a dose-dependent analgesic effect in two experimental pain models. The results suggest the involvement of opioid, benzodiazepine, and serotonergic receptors, as well as the nitric oxide pathway [214]. Acetophenone 3,5-Diprenyl-4-hydroxyacetophenone, isolated from *Ageratina pichinchensis*, showed potent dose-dependent antinociceptive activity, with an efficacy of 72.6% at 100 mg/kg, higher than that observed with ketorolac [65].

In *Heterotheca inuloides*, 7-hydroxy-3,4-dihydrocadalin (**324**) was identified as one of the main compounds responsible for antinociceptive activity. This metabolite produced a superior effect compared with diclofenac and reduced carrageenan-induced mechanical hyperalgesia and inflammation. The fact that its effect was inhibited by methiothepin suggests the involvement of serotonergic pathways in its mechanism of action [191]. Hofmeisterin III (**183**), along with the thymol (**160**) derivatives: thymol hexanoate and thymol acetate, derived from *Hofmeisteria schaffneri*, showed marked antinociceptive activity at low doses (1 mg/kg). The reversal of this effect by naloxone suggests the involvement of the opioid system [177]. The species discussed in this section constitute an important resource in traditional Mexican medicine and have been used to treat toothaches, rheumatism, visceral inflammatory processes, wounds, gastrointestinal disorders, ulcers, and other inflammatory conditions. The available pharmacological evidence supports several of these traditional applications and highlights Mexican Asteraceae as an important source of bioactive metabolites with analgesic and antinociceptive potential [191,214].

#### 3.3.2. Antidiabetic

Type 2 diabetes mellitus (T2DM) is a chronic metabolic disease characterized by a progressive, non-autoimmune decline in adequate pancreatic β-cell insulin secretion, often accompanied by insulin resistance and resulting in persistent hyperglycemia [215]. In 2021, approximately 508 million people worldwide were living with T2DM, corresponding to 5.9% of the global population. By 2050, this number is projected to rise to 1,27 billion people, corresponding to a global prevalence of approximately 9.5% [216]. Preclinical and clinical studies have investigated the potential of Asteraceae species and their bioactive constituents for the management of T2DM, as well as for their associated risk factors.

Recent studies have shown the hypoglycemic activity of several Mexican plants, such as the aqueous extract of *Ageratina petiolaris* (160 mg/kg), which significantly reduced non-fasting blood glucose by approximately 33% relative to baseline and prevented the increase in HbA1c, while also reducing triglyceride exposure. In contrast, *Calea urticifolia* (41 mg/kg) did not markedly lower blood glucose but prevented the progressive worsening of hyperglycemia, confirming the long-term efficacy of both plants in the control of chronic hyperglycemia [217]. Phytochemical analysis of *Stevia serrata* root decoction led to the isolation of dammaradiene acetate, stevisalioside A, and three novel glycosides, stevisaliosides B–D. Administration of stevisalioside A (5 and 50 mg/kg) significantly reduced blood glucose levels and postprandial glycemic excursions in STZ-hyperglycemic mice. In contrast, stevisalioside D demonstrated potent inhibition of PTP1B [218].

*Dahlia pinnata* also demonstrated antihyperglycemic and antihyperinsulinemic effects following oral administration of petal extract at 3.3 and 10 mg/kg in mice fed a high-fat diet. In this study, chemical and pharmacological analyses identified the chalcones: butein and isoliquiritigenin, as well as the aurone: sulfuretin, as relevant bioactive constituents. The combinations of butein with isoliquiritigenin or sulfuretin significantly improved glucose tolerance, whereas the combination of isoliquiritigenin and sulfuretin was ineffective and showed that this glucoregulatory effect depends on PI3K, as previously mentioned. This group was also reported in a clinical study involving 13 participants with prediabetes or T2DM that acute administration of *D. pinnata* extract induced a dose-dependent improvement in glucose tolerance during an oral glucose tolerance test, being most effective in five patients diagnosed with T2DM by HbA1c ≥ 48 mmol/mol [219].

*Brickellia eupatorioides* ethanolic extract (100 mg/kg) significantly reduced blood glucose in alloxan-induced diabetic rats. Unlike previous studies in which isolated compounds exerted hypoglycemic effects by modulating insulin signaling or PI3K, *B. eupatorioides* extract activity was mainly associated with α-glucosidase inhibition, suggesting a complementary mechanism involving reduced carbohydrate digestion and postprandial glucose availability [220]. A complementary mechanism has also been identified for *Psacalium decompositum*. A fructooligosaccharide fraction (150 mg/kg) increased beneficial and butyrate-producing intestinal bacteria in Wistar rats. These results suggest that the hypoglycemic effects of *P. decompositum* may also involve modulating the gut microbiota and increasing short-chain fatty acid production [221].

The discrepancies among these studies may be related to differences in extract composition and standardization, dose and treatment duration, concomitant pharmacological and dietary treatment, as well as the initial metabolic characteristics of the experimental models or patients. Asteraceae species and their bioactive constituents show potential for managing T2DM through several complementary mechanisms. However, further research is needed to identify the active compounds and confirm whether preclinical results translate into consistent clinical benefits.

#### 3.3.3. Antidiarrheal

Diarrhea is widespread passage of loose stools, and it can be acute or chronic and caused by infectious organisms, food intolerance, drugs, or intestinal disease. Acute diarrhea persists for less than two weeks, and it is divided into noninflammatory and inflammatory. Noninflammatory diarrhea is associated with toxin-producing bacteria [222]. Toxin-producing bacteria or agents are commonly *Staphylococcus aureus*, *Escherichia coli* and *Vibrio cholerae*, and protozoa that disrupt the normal absorption of nutrients. Inflammatory diarrhea is associated with invasion of intestinal cells caused by *Shigella*, *Salmonella*, *Yersinia*, and *Campylobacter*. Chronic diarrhea is characterized by persisting for four weeks or more, and it is caused by hyperosmotic luminal contents, increased intestinal secretory processes, inflammatory conditions, and infectious processes. Secondary metabolites isolated from plants of the Asteraceae family have shown to be an effective treatment of diarrhea in experimental models. A moderate to good decrement in the hyperpropulsive movement of the small intestine in rats was observed by thymol derivatives, obtained from *Ageratina cylindrica* and *A. glabrata*: cylindrinol A (**165**) with ID_50_ = 0.85 ± 0.005 μmol/kg, and cylindrinol F (**170**) with ID_50_ = 0.008 ± 0.001 μmol/kg; while 10-benzoyloxy-8,9-epoxy-6-hydroxythymol isobutyrate (**173**) displays ID_50_ = 2.00 ± 0.003 μmol/kg, and 10-benzoyloxy-8,9-dehydro-6-hydroxythymol isobutyrate (**176**) has an ID_50_ = 1.43 ± 0.006 μmol/kg [55,60]. A plausible relationship between the structure–antidiarrheal effect was associated with presence of the phenolic group in thymol derivatives [57].

#### 3.3.4. Anti-Inflammatory

Inflammation is an immediate and nonspecific innate protective response of vascularized tissues to infectious or noninfectious injury, characterized by heat, redness, swelling, pain, and loss of function. It aims to remove the cause of injury, clear damaged tissue, and restore homeostasis [223]. Recently, six studies have examined bioactive compounds from Mexican Asteraceae species for their anti-inflammatory activity. Sesquiterpenoids and sesquiterpene lactones are especially important because they modulate key inflammatory mediators and signaling pathways involved in the inflammatory response.

In a murine model of carrageenan-induced inflammation, the local peripheral administration of 1–30 μg of zinagrandinolide E (**353**) to the mouse paw induced dose-dependent acute anti-inflammatory effects, comparable to diclofenac, and involved the opioidergic, serotoninergic, and GABAergic systems, as well as the NO-cGMP-ATP-sensitive K^+^ channel signaling pathway [195]. A study with cacalol acetate (**270**), obtained from *Psacalium decompositum*, induced an anti-inflammatory effect through NF-KB signaling pathway and a decrease in proinflammatory mediators (TNF-α, IL-1β e IL-6) in Raw 264.7 macrophages [188]. *Tagetes parryi* essential oil demonstrated significant anti-inflammatory activity, reducing ear edema by 93.8% in a chronic model of inflammation. Its major constituent, verbenone, achieved 81.1% inhibition. In lipopolysaccharide-stimulated macrophages, the essential oil also reduced the production of NO, TNF-α, and IL-6, indicating both local and cellular anti-inflammatory effects [224]. Also, in a monoarthritis model, administration of *Baccharis conferta*-derived kingidiol (**427**) (10 mg/kg) significantly reduced joint inflammation and lowered TNF-α, IL-1β, IL-6, and IL-17 levels. These effects were associated with decreased inflammatory cell infiltration and diminished cartilage damage [157]. Recent studies have demonstrated that *Montanoa tomentosa* exhibits significant anti-inflammatory activity in ear edema. Among its bioactive constituents, 15-cinnamoyl kaurenoic acid was identified as the most active compound, producing approximately 79% inhibition of edema. These findings support the role of kaurane-type diterpenoids as important anti-inflammatory constituents of Mexican Asteraceae plants [225].

In summary, these results show that species of the Asteraceae family are a significant source of anti-inflammatory bioactive compounds, especially terpenoids, including sesquiterpenes, sesquiterpene lactones, and kaurane-type diterpenes, which inhibit inflammatory responses by various mechanisms. Additionally, the effects of zinagrandinolide E (**353**), cacalol acetate (**270**), verbenone, kingidiol (**427**) and 15-cinnamoyl kaurenoic acid show that Mexican Asteraceae also serve as valuable sources of pharmacologically active terpenoids. Yet, since the current evidence is mostly preclinical, further research into pharmacokinetics, toxicity, molecular selectivity, dose standardization, and clinical efficacy is needed before these compounds can be regarded as viable therapeutic options for inflammatory diseases.

#### 3.3.5. Antibacterial Activity

Microorganisms like bacteria cause many infectious diseases and death. They have the capability of autonomous replication, and the treatment of their infections is controlled by the administration of chemotherapeutic agents such as antibiotics [226]. Nevertheless, bacteria can develop the ability to defeat the drugs designed to kill them or limit their growth by natural mechanisms or through developing multi-resistance (MDR) due a misuse of antibiotics [227,228]. Bacterial MDR is a very public health concern, and the WHO has made a call to attention to discover and develop new antibiotics, which pivot the exploration of the antibacterial properties of natural products. The main class of specialized metabolites derived from Mexican Asteraceae species that are active against bacteria comprised terpenoids from *Ageratina deltoidea* [58], *Baccharis conferta* [149], *Gymnosperma glutinosum* [229], *Heliopsis longipes* [203,230], *Iostephane heterophylla* [92,231], and *Psacalium radulifolium* [117].

The compounds that were active against Gram-positive bacteria were an alkaloid (**489**), a phenolic derivative (**116**), and the sesquiterpenes **269, 297**, **348**, **349**–**351**, and **380**; the diterpenes **407**–**408** and **438**; and the triterpene **453**; it should be noted that most of them are active against *Staphylococcus aureus*. The most active were juniperin (**355**) with a MIC at 8 μg/mL, *ent*-trachyloban-19-oic acid (MIC 8.9 μg/mL), and 4-(1′,5′-dimethylhex-4′-enyl)-2-methylphenol (**380**, MIC 16 μg/mL) [92,98,231], followed by cacalol (**269**, MIC 12 μg/mL) [117], xanthorrhizol (MIC 16 μg/mL) [92,232], a mixture of grandiflorenic acid (**440**) + *ent*-kaurenoic acid (**444**) with a MIC of 31 μg/mL [58], zinaflorin VI (**349**, MIC 64 μg/mL) [98], and 6-*O*-tigloyl-zinaflorin II (**351**, MIC 64 μg/mL) [98]. Other individual constituents with antibacterial effect were: radulifolin B (**297**) [117], 5-(3-buten-1-ynyl) -2,2′-bithienyl (**147**) [125], 8β-angeloyloxy elemacronquistianthus acid (**348**) [58,156], *ent*-dihydrotumanoic acid (DTA, **438**) [229], and cativic acid (**407**) [63].

In the case of Gram-negative bacteria, the active compounds were an alkaloid (**489**), the phenolic derivatives **116** and **118**, four sesquiterpenes (**211**, **269**, **348**, **380**), one diterpene (**438**), and one triterpene (**453**). The sesquiterpene lactone named incomptine B (**218**) was the most active against *Escherichia coli* and *Shigella flexneri*, with MIC values of 0.4 and 0.5 μg/mL, respectively [181]. Other antimicrobial compounds were cacalol (**269**) [117], deltoidin A (**211**), affinin (**491**), and 8*β*-angeloyloxy elemacronquistianthus acid (**348**), which were obtained in the highest amounts from *Iostephane heterophylla* [92,231].

Tuberculosis is another infectious disease with concerning antibiotic resistance. The specialized metabolites with activity against *Mycobacterium tuberculosis* include: three sesquiterpenes (**166**, **300**–**301**) and an aromatic compound (**97**). In addition, the sesquiterpene lactones santamarine (**252**) and reynosin (**253**) were active against a strain of *M. tuberculosis* (Mtb H37Rv) [85]. The aromatic compounds, methyl-4-hydroxybenzoate and 3,5-dihydroxybenzoic acid (**92**) had a MIC of 3.12 and 50 μg/mL [53], and the diterpene, *ent*-dihydrotumanoic acid (**438**, DTA), showed moderate antibacterial effect (MIC = 125 μg/mL) against *Mycobacterium smegmatis* and *Mycobacterium phlei* [229].

#### 3.3.6. Antifungal Activity

Human fungal infections are caused by fungi like *Candida albicans* and *Trichophyton spp*, among others. *Candida albicans* is the most common fungus that causes vaginal yeast infections [226]. The *n*-hexane extract of *Psacalium radulifolium* displayed activity against *Candida albicans*. From this extract, cacalol (**269**) was isolated, which showed high activity (MIC 0.012 mg/mL). This suggests that the latter is responsible for the anticandidal activity associated with the plant [117]. Other compounds with anticandidal properties were espeletone with MIC = 100 μg/mL and (+)-β-eudesmol (**243**) at MIC = 25 μg/mL [64].

In the case of the *Trichophyton* species, they were susceptible to the growth inhibition by aromatic compounds (**132**, **134**), sesquiterpenes (**292**, **299**), and one triterpene (**471**). The most active were encecalin (**134**) with a MIC of 6.2 μg/mL, encecalinol (**132**, MIC = 12.5 μg/mL), (+)-β-eudesmol (**243**, MIC = 25 μg/mL) [117], 3β-angeloyloxy-6β-hydroxyeremophil-1(10)-en-8β,12-olide (**299**, MIC = 125 μg/mL), 3β-angeloyloxy-6β-hydroxyeremophil-1(10)-ene (**292**, MIC = 250 μg/mL) [96,121], and taraxerol (**471**, MIC =12.5 μg/mL) [64,121].

Fungi of relevance for crops are *Fusarium moniliforme*, *Aspergillus niger*, *Phytophthora infestans*, *Rhizoctonia solani*, *Sclerotium cepivorum*, and *Sclerotium rolfsii*. The first species is susceptible to growth inhibition by action of 3β-angeloyloxy-6β-hydroxyeremophil-1(10)-en-8β,12-olide (**299**) [121], and 3β-angeloyloxy-6β-hydroxy-eremophil-1(10)-ene (**292**) [121]. The *Aspergillus niger* was inhibited by 5-acetyl-3β-angeloyloxy-2β-(1-hydroxyisopropyl)-6-methoxy-2,3-dihydrobenzofurane (**145**, MIC = 50 μg/mL), 4-hydroxy-3,5-diprenylacetophenone (MIC = 100 μg/mL), and espeletone (MIC = 200 μg/mL) [171], while *Phytophthora infestans*, *Rhizoctonia solani* AG3, *Sclerotium cepivorum*, *Sclerotium rolfsii* were 100% inhibited by affinin (**491**) at 150 μg/mL [204].

#### 3.3.7. Antiprotozoal

Several microbial agents can infect the gastrointestinal tract, causing diarrhea, ulcers, and inflammation in the small or large intestine. Gastrointestinal protozoa that produce damage or infections are *Entamoeba histolytica*, *Giardia lamblia*, and *Trypanosoma cruzi* [226,233,234].

Amebiasis refers to infection by *Entamoeba histolytica*. It damages the small or large intestine, increasing simple diffusion and cell death by trophozoites. Metabolites that are active against this protozoan were isolated from *Ageratina glabrata* (**165**–**175**) [55,56,60], *Ageratina cylindrica* (**165**–**166**, **169**–**170**, **462**) [55,57], *Laennecia filaginoides* (as *Conyza filaginoides)* (**454**), *Decachaeta incompta* (**218**–**221**), *Neurolaena oaxacana* (**203**), *Parthenium hysterophorus* (**343**), *Tagetes filifolia* (**114**) and *Vernonia liatroides* (**216**), and belong to the class of phenolics and terpenoids. The most active metabolites against *E. histolytica* were 10-benzoyloxy-8,9-epoxy-6-hydroxythymol isobutyrate (**173**, IC_50_ = 1.6 μg/mL), 10-benzoyloxy-8,9-epoxythymol isobutyrate (**175**, IC_50_ = 25.9 μg/mL), incomptine A (**219**, IC_50_ = 2.6 μg/mL), neurolenin B (**203**, MIC = 1.6 μg/mL), erythrodiol (**454**, IC_50_ = 12.7 μg/mL), (quercetin 3-O-(6″-*O*-*E*-caffeoyl)-β-D-galactopyranoside (**79**, IC_50_ = 14.9 μg/mL), isoquercitrin (**80**, IC_50_ = 14.7 μg/mL), and narcissin (**81**, IC_50_ = 17.2 μg/mL). The in vitro study of the cellular and molecular mechanisms that trigger an amoebicidal effect by incomptine A (**219**) evidenced that this sesquiterpene lactone generates an increase in the number of glycogen granules and inhibits key enzymes of the glycolytic metabolism on trophozoites of *Entamoeba histolytica* [233].

A second protozoan that produces dysentery, which is accompanied by mechanical obstruction of nutrients and inflammation, is *Giardia lamblia*. Mexican Asteraceae species and their specialized metabolites have displayed significant giardicidal effects [143], specifically the following plants: *Ageratina cylindrica* (**165**) with IC_50_ = 167.4 μM [55], *Ageratina glabrata* (**173**) with IC_50_ = 36.9 μM [60], cylindrinol B (**166**) with IC_50_ = 251.9 μM, cylindrinol E (**169**) with IC_50_ = 164.5 μM, cylindrinol F (**170**) with IC_50_ =151.1 μM [57], as well as, *ent*-15β-(β-L-fucosyloxy)kaur-16-en-19-oic acid β-D-glucopyranosyl ester (**449**), *ent*-15β-(4-acetoxy-β-L-fucosyloxy)kaur-16-en-19-oic acid β-D-glucopyranosyl ester, *ent*-15β-(3-acetoxy-β-L-fucosyloxy)kaur-16-en-19-oic acid β-D-glucopyranosyl ester, and *ent*-15β-(β-L-fucosyloxy)kaur-16-en-19-oic acid with IC_50_ values of 41.9, 69.5, 48.9, and 98.5 μM, respectively [56]. From *Laennecia filaginoides* (as *Conyza filaginoides*), the flavonols known as: isorhamnetin 3-O-(6″-O-E-caffeoyl)-β-D-galactopyranoside (**78**) and nicotiflorin (**76**), and the triterpene erythrodiol (**454**), have IC_50_ values of 15.3 μg/mL, 22.5 μg/mL and 29.9 μg/mL, respectively [161]. The sesquiterpenes **323** and **324**, from *Heterotheca inuloides*, exhibited IC_50_ values of 15.3 and 22.96 μg/mL [153]. The heliangolides **218**–**221** derived from *Decachaeta incompta* displayed antiprotozoal effects in the range of 18.1 to 256.7 μg/mL [181,182,232]. In addition, neurolenin B (**203**), derived from *Neurolaena oaxacana*, displayed a MIC of 3.2 μg/mL [109].

American trypanosomiasis is recognized as a neglected tropical disease. Recently, the WHO has aimed to increase global networking to eliminate Chagas disease transmission [235]. Phytochemistry of the Asteraceae family has given compounds of interest for research into their trypanocidal activity against *T. cruzi*. Ambrosin (**343**), incomptine B (**218**) and glaucolide E (**216**) are metabolites with this activity, ranging 67.1 to 215.13 μM, and all of them can be considered as candidates for development of trypanocidal agents [73].

#### 3.3.8. Cytotoxic and Antitumor Activities

Cancer is one of the principal causes of death around the globe [234]. It is caused by a diverse and complex etiology, such as genetic and molecular mechanisms involved in transforming normal cells to cancer cells, and external factors that contribute to it [226]. The systematic search for anticancer drugs of plant origin dates back to the second half of the last century [232,236]. Thus, the continuous search for natural products for developing chemotherapeutic agents for cancer treatment has been extended to native and endemic plants from Mexico belonging to the Asteraceae family. The Asteraceae plants that have displayed cytotoxic or antitumor effects against mammalian or human carcinomas are *Acourtia cordata* [102], *Decachaeta incompta* [182], *D. perornata* [186], *Gymnosperma glutinosum* [199], *Mikania cordifolia* [78], *M. micrantha* [78], *Parthenium argentatum* [200], and *Verbesina persicifolia* [96]. From these plants, several kinds of specialized metabolites have been identified and fully characterized. Among these, sesquiterpenes and sesquiterpene lactones are the most representative groups with cytotoxic activity. Perezone (**391**) has been identified as a cytotoxic compound against K562 cells with IC_50_ of 26.94 μM. It induced apoptosis in a concentration-dependent manner and inhibits the enzyme PARP-1, which is overexpressed in various carcinomas [102]. Incomptine A (**219**) showed cytotoxic activity against U-937 (histiocytic lymphoma) with an IC_50_ value of 0.12 μM. The study of its molecular action mechanisms suggests that **219** modifies protein expression related to apoptosis, oxidative stress response, DNA repair, cell cycle checkpoints, glycolysis, cytoskeleton organization, NF-κB signaling, and autophagy [74]. At this point, it is worth noting that the comparison of cytotoxicity between non-tumorigenic and tumorigenic cells is still a necessity and represents a limitation that should be solved in future studies. The in vivo anti-lymphoma effect of **219** was confirmed in a murine model. The antitumor activity of incomptine A (**219**, EC_50_ = 1.3 ± 0.26 mg/kg) was comparable with the one observed for metotrexate (EC_50_ = 2.4 ± 0.10 mg/kg), used as a reference drug [237]. Subsequent studies expanded the exploration of the cytotoxic properties of **218** and **219** in breast (4T1, MDA-MB-231, SK-BR-3, T-47D, and MCF-7) and leukemia carcinoma cells (HL-60, K-562 and REH). Both lactones displayed cytotoxicity against the set of leukemia cells in the range of 0.3 to 2.1 μM [238]; and **219** showed cytotoxicity against the panel of breast cancer cells in the range of 18.53 to 43.11 μM. The lesser cytotoxic effect of **219** against the nontumorigenic cell line MCF-10A in comparison with the MCF-7 cell line displayed a selectivity index of 1.9, highlighting its potential to be used in cancer treatment [239]. The antitumor activity of **219** in a murine model of breast cancer followed histopathological analyses of its effects and by flow cytometry, western blot and mitochondrial activity assays, which evidenced that this compound possesses antitumor activity in a dose-dependent way, which induces an increment in the expression of caspase-3, reduces the expression of hexokinase II and bcl-2 proteins, and induces apoptosis and reduces the mitochondrial activity [240]. The in vivo anti-lymphoma effect of **219** and the exploration of the molecular action mechanisms associated with its cytotoxicity in non-Hodkin lymphoma cells U-937 using tandem mass tag, Gene Ontology, reactome, KEGG pathway analysis, and molecular docking showed that the treatment with heliangolide **219** induces a differential expression of proteins [241]. This differential expression of proteins was confirmed through a quantitative proteomics approach and was associated with crucial cellular processes such as chromatin remodeling, metabolic and energetic processes such as glycolysis/gluconeogenesis, oxidative phosphorylation, cytoskeletal organization, cell proliferation, and with cell death, among others [242,243]. Based on the pharmacological properties of the isolates from *D. incompta*, the targeted cytotoxicity assaying of extracts and specialized metabolites from other *Decachaeta* species was conducted to identify ivalin (**258**) as the main cytotoxic constituent in *D. perornata*, with IC_50_ values of 1.61, 3.99 and 5.64 μM against the cancer cell lines HeLa, HCT-116 and MCF-7, respectively [186].

From *Heterotheca inuloides*, the sesquiterpenes: 7-hydroxy-3,4-dihydrocadalene (**324**) and 7-hydroxycadalene (**323**) showed high toxic effects against *Artemia salina* larvae (4.0 and 3.6% viability, respectively) and RAW 264.7 cells, so they were subsequently assayed to improve the cytotoxic effects of doxorubicin and mitoxantrone in multidrug-resistant MES-SA/MX2 cell line [169,192]. The cytotoxicity assaying of sesquiterpene lactones from *Mikania* species led to identification of miscandenine (**358**), dihydromikanolide (**222**), deoxymikanolide (**223**), 8-*epi*-mikanokryptin (**341**), and achalensolide (**340**) as antiproliferative compounds against cell lines: HCT-15, K562, PC-3, U251, MCF-7, and SKLU-1 in the range of 1.9 to 9.5 μM [78].

From *Verbesina persicifolia*, the sesquiterpene named 4β-cinnamoyloxy-1β,3α-dihydroxyeudesm-7,8-ene (**247**) showed cytotoxic activity against the carcinoma cells: A431 (human epidermoid), MSTO-211H (human biphasic mesothelioma), HL-60 (human promyelocytic leukemia), HepG2, and HeLa with GI_50_ values in a concentration range of 14.7 to 32.3 μM [96]. From *Parthenium argentatum*, the triterpenes argentatin A (**468**) and B (**469**) have been assayed against a set of several cancer cell lines. The first exhibits cytotoxicity against proliferating lymphocytes at 25 μM, and the second is cytostatic in the range of concentrations from 5 to 25 μM [200]. The study of the underlying molecular mechanism that produces cytotoxic effects or induces arrest of the cell cycle, induced by specialized metabolites from Mexican Asteraceae species, and their pharmacological optimization through semi-synthesis, as well as the in vivo assaying of these, constitutes current trends in pharmacognosy research.

#### 3.3.9. Diuretic Effect

Diuretics increase urine output and facilitate the renal excretion of water and electrolytes, especially sodium. While this pharmacological effect is commonly utilized in the management of hypertension and fluid retention, diverse natural products have also demonstrated diuretic activity via several renal mechanisms. In this regard, a diterpene isolated from *Gymnosperma glutinosum* showed diuretic effects. *Ent*-dihydrotumanoic acid (**438**, DTA) exerts urinary excretion (ED_50_ = 86.9 mg/kg), decreases the urinary pH, and stimulates the excretion of Na^+^ (ED_50_ = 66.7 mg/kg) and K^+^ (ED_50_ = 8.6 mg/kg). The effect of its high concentration was like furosemide [244].

#### 3.3.10. Gastroprotective Effect

Gastric diseases are related to injuring the epithelial and deeper layers of the stomach wall as ulcers. The principal causes of gastric irritation are NSAIDs and infection with *Helicobacter pylori* [226]. The specialized metabolites from Asteraceae, active against *H. pylori* include the sesquiterpenes estafiatin (**335**) with a MBC of 15.6 μg/mL, 7-hydroxy-3,4-dihydrocadalene (**324**), and 7-hydroxycadalene (**323**) with a MIC of 1.95 and 3.91 μg/mL, respectively, and the flavonoids: eupatilin (**42**) with a MBC of 31.2 μg/mL and 7,3′-di-*O*-methyleriodictyol (**40**) with a MIC of 3.91 μg/mL, which were isolated from *Artemisia ludoviciana* subsp. *mexicana* [38] and *Heterotheca inuloides* [152]. This flagellated bacterium produces an enzyme with activity of urease to generate an optimal environment [245]. Thus, the flavonol **68** showed high in vitro inhibitory activity on this enzyme with MIC 15.63 μg/mL [152]. Gastroprotective activity on damage caused by ethanol administration was described for the phenolic compound 3,5-diprenyl-4-hydroxyacetophenone. Nevertheless, the best activity values were obtained with the sesquiterpenes calein D (**197**) [106] and juanislamin (**198**) at 10 mg/kg [65]. Other isolates that were evaluated as gastroprotective agents with less activity are 3,5-diprenyl-4-hydroxyacetophenone and encecanescin [65,246].

#### 3.3.11. Immunostimulant Effect

The human body is still constantly alert to defend against xenobiotics or microorganisms. It is known that the immune system recognizes pathogens and/or foreign substances and is constituted by cells and biochemical defenses. However, they become responsible for pathophysiological processes when the system is suppressed [226]. The sesquiterpene maturin acetate (**290**), isolated from *Psacalium peltatum* exerts many processes on the immune system. It has been identified to induce releasing immunomarkers (IL-2, IL-15, and IFN-γ) in immunosuppressed mice, stimulate the NK cells, pinocytosis, and lysosomal enzyme activity at 25 μM, and it also improved NO production [187].

#### 3.3.12. Effects on the Central Nervous System

Recent studies have identified several compounds derived from the Asteraceae family that exhibit neuroprotective and cognitive effects in experimental models of CNS disorders. These effects are mediated through the modulation of neuroinflammation, oxidative stress, and neurotransmission.

Among the Mexican Asteraceae species investigated for their effects on the CNS, *Tagetes lucida* Cav. has shown relevance due to the neuroactive properties of its coumarins. The 7-isoprenyloxycoumarin (7-*O*-prenyl-umbelliferone) (**129**) and herniarin (7-methoxycoumarin) (**126**), isolated from a hexane-soluble extract of *T. lucida*, were evaluated in a murine model of lipopolysaccharide-induced neuroinflammation and memory loss. Subsequently, they received 0.25–2 mg/kg of 7-isoprenyloxycoumarin (**129**) or herniarin (**126**). These coumarins induced a neuroprotective effect by improving spatial learning in the Morris water maze and reducing LPS-induced hyperactivity in the open-field test. Both compounds were associated with changes in proinflammatory cytokine concentrations (TNF-a, IL-1, and IL-6). Their levels were significantly reduced compared to the vehicle treatment group. Therefore, the coumarins 7-isoprenyloxycoumarin (**129**) and herniarin (**126**) protect the CNS by counteracting inflammation and cognitive impairment secondary to lipopolysaccharide administration, and these molecules can act as immunomodulators [247]. An additional coumarin identified in this species includes dimethylfraxetin (**128**). Extracts of several polarities from the plant, such as hexane, ethyl acetate, methanol, and aqueous, were evaluated for their effects on catalepsy and psychotic behaviors in mice. Herniarin (**126**), dimethylfraxetin (**130**), as well as *T. lucida* ethyl acetate and aqueous extracts, reduced stereotypical behaviors, hyperlocomotion, memory impairments, and deficits in social interaction. These coumarins also altered the response to haloperidol, potentially through modulation of the glutamatergic system and by influencing interactions between dopaminergic and glutamatergic pathways [248]. In a cuprizone-induced neuroinflammation and motor impairment model, standardized extracts and coumarins from *T. lucida* reduced motor dysfunction and neuroinflammation by decreasing IL-1β and TNF-α and increasing IL-4 and IL-10, while also preserving blood–brain barrier integrity [249]. Recent studies have shown that the aqueous extract of *Calea ternifolia* (reported as *Calea zacatechichi*) interferes with cholinergic and dopaminergic pathways by inhibiting acetylcholinesterase and tyrosinase. Neurocytotoxic effects have also been observed in neuronal and microglial cells, emphasizing the importance of evaluating both the neuroactive and toxicological properties of Mexican Asteraceae plants [250].

In summary, the findings indicate that Mexican Asteraceae contain neuroactive compounds capable of modulating neuroinflammation, cognitive function, and neurotransmission. However, the neurotoxicity observed in specific extracts highlights the need for further investigation into safety, dose–response relationships, and molecular mechanisms before their therapeutic potential for central nervous system disorders can be established.

#### 3.3.13. Vasoactive Effect

The regulation of vascular tone depends largely on the endothelium, a single layer of cells that synthesizes and releases various vasoactive mediators, maintaining the balance between vasodilation and vasoconstriction. Among these mediators is nitric oxide (NO), a major vasodilatory agent [251]. Endothelial dysfunction reduces NO bioavailability, promoting the development of cardiovascular diseases, including hypertension, considered one of the leading causes of premature death worldwide [251,252]. However, not all vasoactive mechanisms depend on the endothelium, as some compounds can exert a direct effect on vascular smooth muscle. Several extracts from Asteraceae species have been evaluated for their vasoactive activity modulated through different signaling pathways. Among them, the methanolic extract of *Brickelia cavanillesii* induced concentration-dependent vasorelaxation in both aortic rings with and without endothelium (IC_50_ = 3.82 µg/mL with endothelium and 3.63 µg/mL without endothelium). *B. cavanillesii* showed a greater vasorelaxant potential and the endothelium-independent activity suggests a direct action on vascular smooth muscle [253]. Dichloromethane and ethanol-soluble extracts of *Heliopsis longipes* roots, as well as affinin (**489**) isolated from this species, induced a concentration-dependent vasodilation in aortic rings of rats. Unlike the previous extracts, the activity of affinin (**489**) was partially endothelium-dependent and involved gasotransmitters such as NO, H_2_S, and CO, as well as the prostacyclin signaling pathway [91]. A hexane extract from *Achillea millefolium* flowers and the epimeric sesquiterpene lactones, leucodin (**333**) and achilin (**334**), showed vasorelaxant activity in rat aortic rings with endothelium. Their effects were modified by L-NAME and ODQ, and by reducing KCl-induced contraction, suggesting the involvement of the NO/cGMP pathway and mechanisms related to Ca^2+^ influx [35]. Similarly, the ethanolic extract of *Tagetes lucida*, as well as its coumarin derivatives 6,7,8-trimethoxycoumarin (**128**), 6,7-dimethoxycoumarin (**123**) and 7-methoxycoumarin (**126**), showed concentration-dependent relaxation of the endothelium in rat aortic rings contracted by noradrenaline [167]. Their results indicate that this activity is mediated, as in *A. millefolium*, by activation of the NO/Cgmp pathway but with the blocking of calcium channels [35]. Two glycosylated sesquiterpene lactones, glaucolides D and E (**216**), were isolated from *Vernonia liatroides*. Their activity was evaluated in rat aortic rings precontracted with KCl and noradrenaline. Both compounds produced concentration-dependent vasorelaxation; however, glaucolide E (**216**) presented greater potential. Its effects were attributed to a possible inhibition of calcium influx through calcium channels [129]. Another metabolite with vasoactive activity is xanthorrhizol, a sesquiterpene isolated from *Iostephane heterophylla*, induced a concentration-dependent relaxation in the rat aorta independently of the endothelium. Pharmacological studies indicated that this effect does not depend on the release of NO or prostacyclins but is probably associated with the inhibition of Ca^2+^ influx through voltage-gated and receptor-activated calcium channels [254].

### 3.4. Agrochemical Applications

Globally, crop pests can cause yield losses of 20% to 40%, with insects accounting for a significant portion of this damage. Furthermore, researchers are currently seeking alternatives to reduce the use of synthetic insecticides and develop more sustainable pest control methods [255]. Plant-produced specialized metabolites involved in insect defense could serve as potential agrochemicals to combat these pests. In Mexico, the Asteraceae family is among the leading families containing species that exhibit insecticidal and nematicidal activities, as well as other agrochemically relevant properties such as phytotoxicity and, for weed control, germination-stimulating compounds like strigolactones [256,257]. For example, root extracts of *Heliopsis longipes* have demonstrated insecticidal activity, mainly attributed to alkamides, especially affinin (**489**), as well as larvicidal activity against *Anopheles albimanus* and *Aedes aegypti* [258]. *Heliopsis longipes* is a plant that has been widely recognized for its economic and cultural relevance, which represents a gap for future studies.

Similarly, acetone extracts from the aerial parts of *Heterotheca inuloides* and its main metabolite, the sesquiterpene 7-hydroxy-3,4-dihydrocadalene (**324**), showed moderate insecticidal activity against *Spodoptera frugiperda* [259]. Likewise, essential oils and acetone extracts of *Tagetes lucida* and *T. erecta* (also reported as *T. patula*) have also shown insecticidal and larvicidal activity. These species contain bioactive metabolites, such as allylanisole, anethole, limonene, methyl eugenol, and β-caryophyllene (**395**), associated with activity against insects, mites, and various phytopathogens, highlighting their potential for the development of plant-based biopesticides [260,261].

In addition, insects that strike crops, such as *Spodoptera litura* are of relevance in agriculture. The sesquiterpenes that have shown moderate activity against this worm are neurolenins B-D (**203**–**205**) and lobatin A (**209**) [184]. *Leptinotarsa decemlineata* and *Myzus persicae* are two insects that affect crops such as potato. An antifeedant effect on their adults was induced by perezone (**391**) [103]. Another important plague is *Epilachna varivestis*, and β-D-glucopyranosyl oleanolate (**463**) showed activity against its larvae form [90].

Researchers have identified various specialized metabolites associated with allelopathy in the Asteraceae family, inhibiting the germination and growth of other species, highlighting their potential as phytotoxic agents for controlling competing plants [175]. The methanolic extract of *Dyssodia tagetiflora* showed a cytoprotective effect on *Vicia faba* (Fabaceae). The study of its major metabolite hyperoside (**75**) suggests that it is responsible for the activity associated with the plant [160]. In contrast, many plants are considered weed crops, like *Amaranthus hypochondriacus* (Amaranthaceae), which was inhibited in vitro by the phytotoxic compounds derived from *Fluorensia cernua* dehydroflourensic acid, flourensadiol (**330**) and methyl orsellinate (**97**) [88].

Another weed crop of global importance is *Echinochloa crus-galli*, which is controlled by *Decachaeta incompta* [262], *Hofmeisteria schaffneri* [76,172], *Podachaenium eminens* [258], and *Barkleyanthus salicifolius* (reported as *Senecio salignus*) [110]. From the first plant, incoptin B (**218**) has been assayed; it shows phytotoxic activity against various weed crops, including *Echinochloa crus-galli* and *Urochloa decumbens*, primarily affecting root development and, to a lesser extent, germination [258]. Other phytochemicals as hofmeisterins I-III (**181**–**183**), 3′,4′,4a′,9a′-tetrahydro-6,7′-dimethylspiro[benzofuran-3(2H),2′-pyrano [2,3-b]benzofuran]-2,4a′-diol (**158**), and caryophyllene oxide (**396**) have been reported to partially inhibit the root elongation [110].

In addition, some phytotoxic metabolites lack selectivity and damage crops, such as *Physalis ixocarpa* (Solanaceae). Its root growth was inhibited (42–53%) and its leaves were bleached by β-caryophyllene (**395**). All previous suggestions are that this sesquiterpene is related to the inhibition of photosynthesis and its dry biomass production [110]. Heliolactone, a carlactone-type compound, was isolated from the root exudates of *Helianthus annuus* and stimulated germination in various root parasitic weeds, including *Striga hermonthica*, *O. cumana*, *O. minor*, *O. crenata*, and *O. aegyptiaca* (reported as *Phelipanche aegyptiaca*) (Orobanchaceae), supporting its use as a weed control strategy [263]. Specialized metabolites isolated from Asteraceae offer an innovative, lower-impact alternative to conventional agrochemicals. However, further research on their efficacy and safety is needed before large-scale application can be considered.

## 4. Conclusions and Future Perspectives

This review aimed illustrate the state of the art in natural product discovery and pharmacological prospecting with Asteraceae species native to Mexico over last 30 years, highlighting the chemical structural diversity of the plants belonging this family and providing a selective overview of the promising specialized metabolites with agrochemical and pharmacological properties. As shown in Figure 29, the chemical and biological prospecting of Mexican Asteraceae plants has resulted in the identification of 94 plants in 263 scientific reports. The main Mexican Asteraceae plants that have been studied are *Hetheroteca inuloides*, *Heliopsis longipes*, *Brickellia veronicifolia*, *Calea ternifolia*, *Decachaeta incompta*, *Baccharis conferta*, *Gymnosperma glutinosum*, and *Psacalium decompositum*. The quantity of compounds identified in the literature concerning Mexican Asteraceae plants is 494 compounds, and the most distinctive class of them are sesquiterpenes. In pharmacological studies, researchers evaluated most compounds in antibacterial, antiprotozoal, cytotoxic and antitumor models. The most active compound with antibacterial properties was the sesquiterpene juniperin (**355**) against Gram-positive bacteria, **218** against Gram-negative bacteria, and a coumarate derivative (**120**) against *Mycobacterium tuberculosis*. In antiprotozoal activity, a monoterpene (**173**) showed great activity against amoebiasis and the sesquiterpene **203** against giardiasis. In cytotoxic and antitumor activities, highlight the sesquiterpene lactones **219**, **223**, and **358**, which showed high activity on several types of human carcinomas, comparable with oncological agents. In agrochemical applications, the phenolics, the sesquiterpenes, their lactones and the monoterpenes were used as herbicides, and as phytoprotectors on *Vicia faba*. Phenolics **97** and **158**, the monoterpenes **181**–**183**, and the sesquiterpenes **244** and **330** displayed phytotoxic effects, while the phenolic **75** showed a phytoprotective effect, which suggests the necessity for more studies on diverse crops to improve the phenotypic properties. The above highlights that the Asteraceae family members constituted a great source of new compounds with biological activity in the period reviewed, so, the biological prospecting still represents a research gap. Most compounds required computational, in vitro, in vivo and preclinical or complementary studies, including their toxicity determination to detect the effects and the study of the molecular mechanisms associated with their bioactivities, which could address the development of new agrochemical and pharmacological agents.

## Figures and Tables

**Figure 1:**
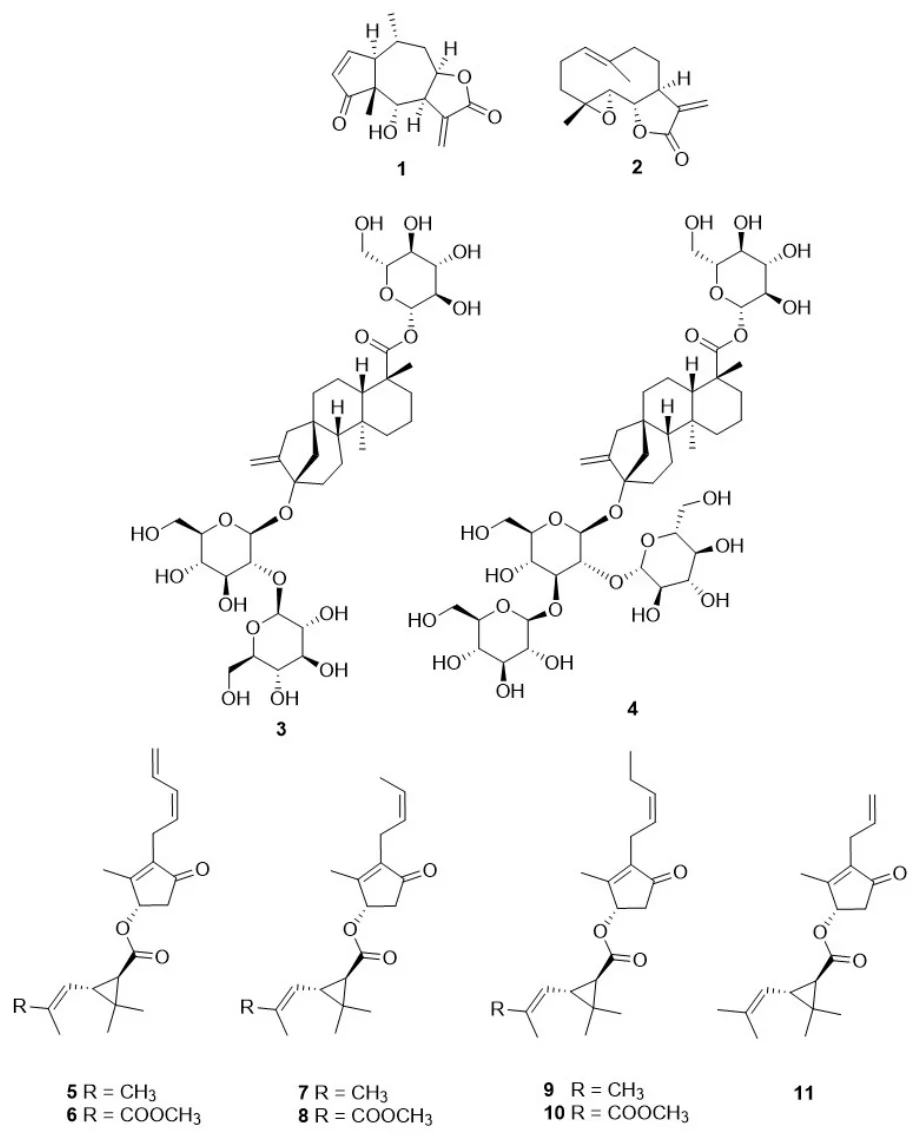
Representative bioactive compounds from Asteraceae species.

**Figure 2:**
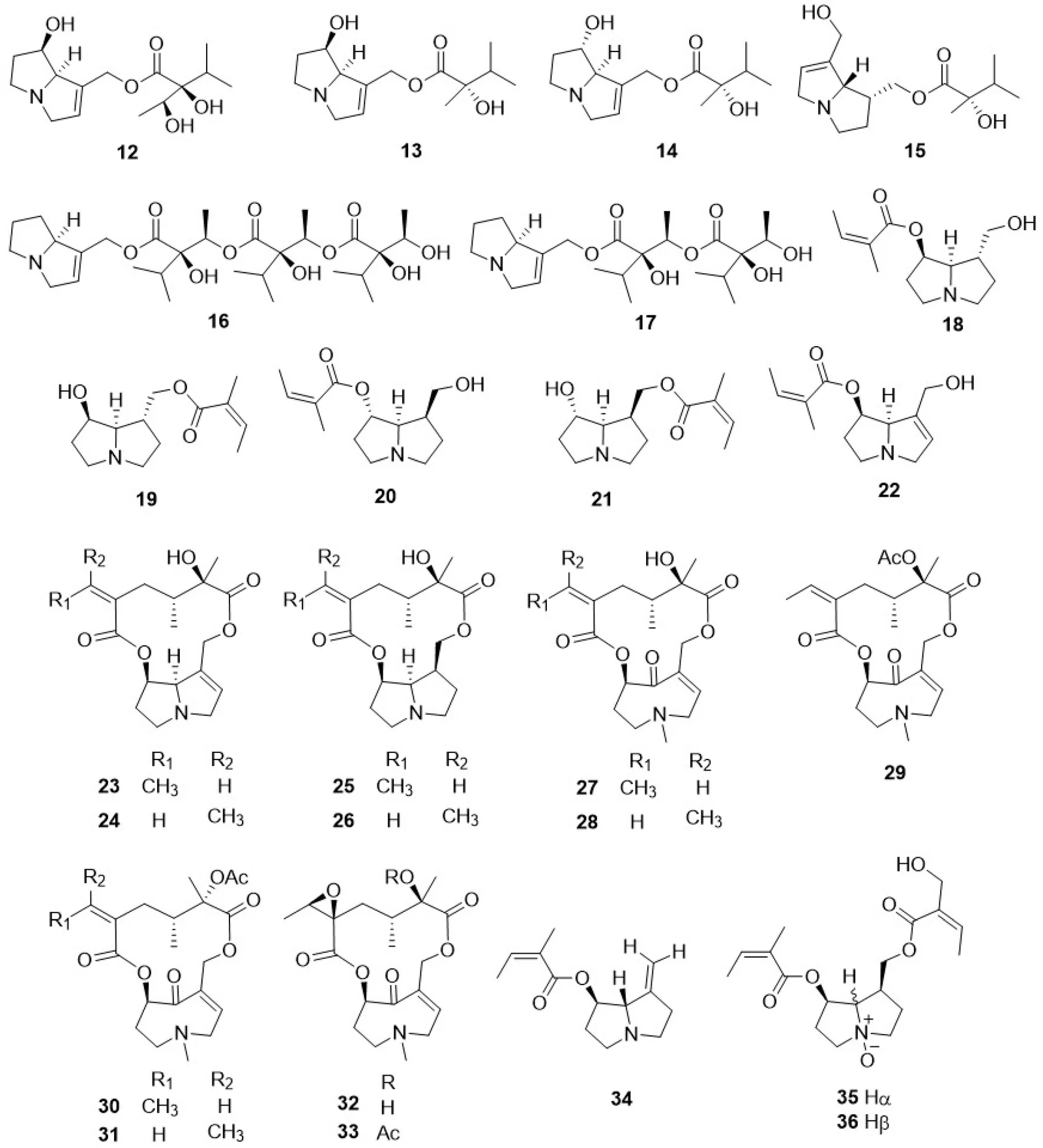
Principal pyrrolizidine-type alkaloids from the Asteraceae family.

**Figure 3:**
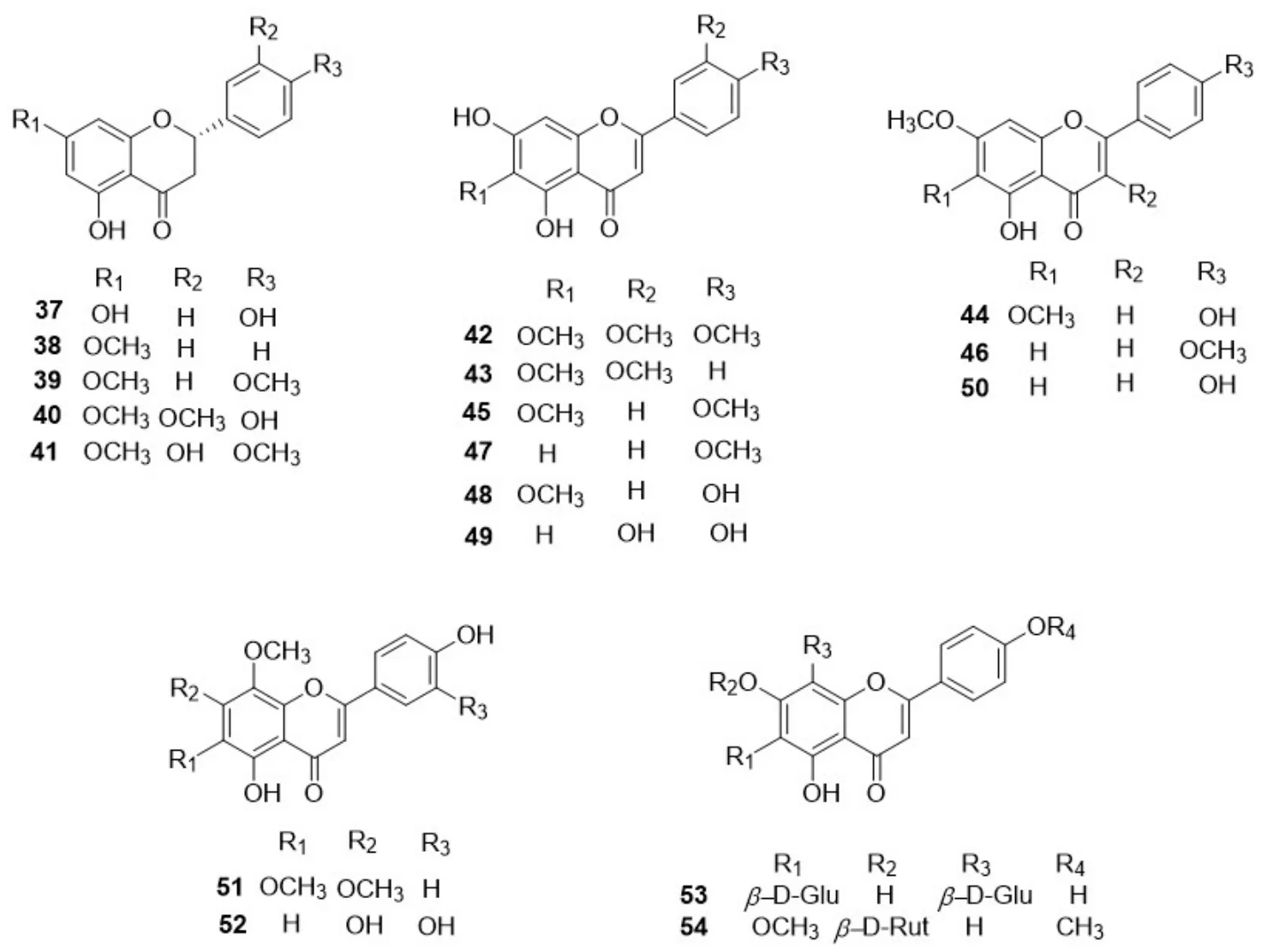

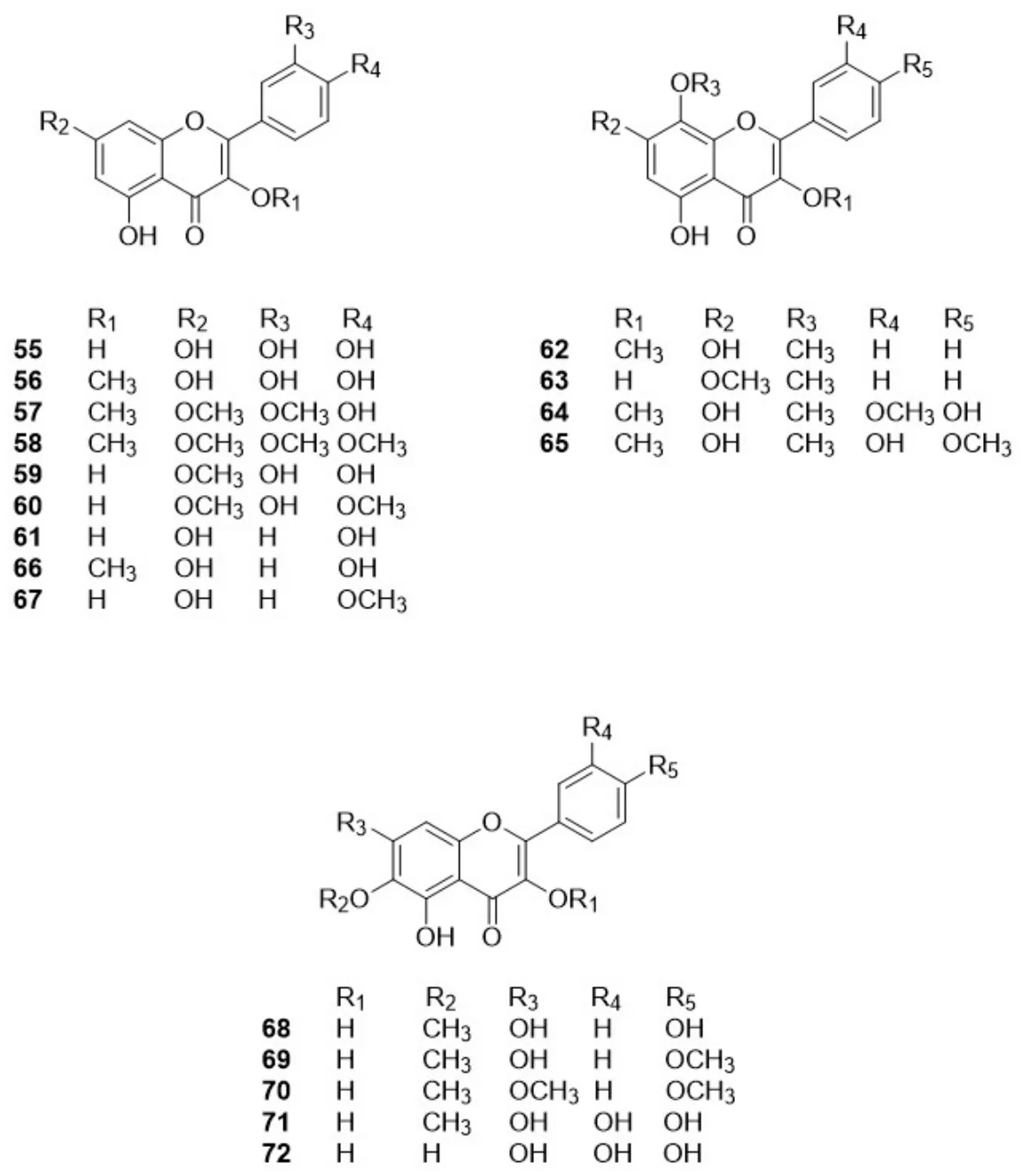

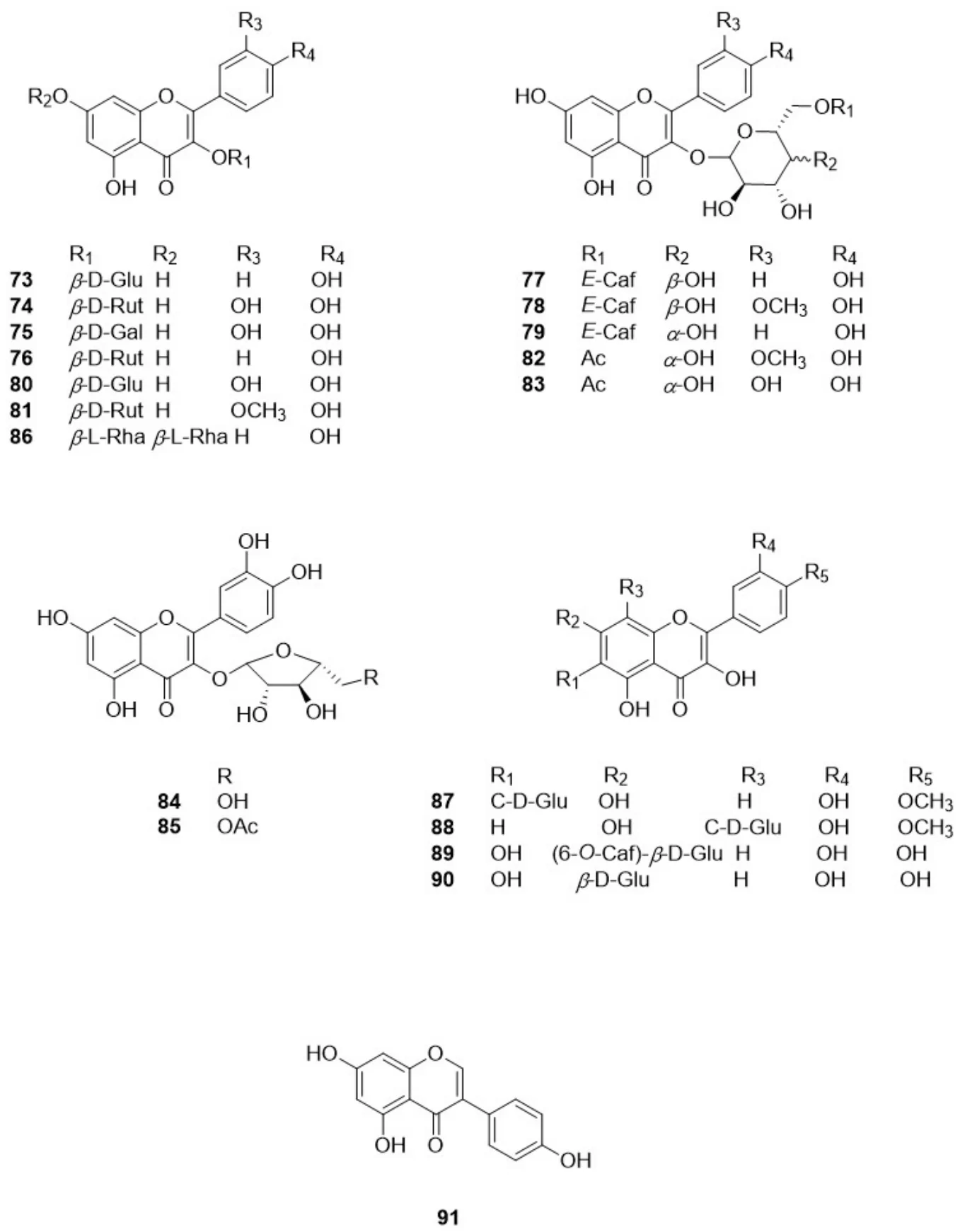
Flavonoids isolated from Mexican Asteraceae species.

**Figure 4:**
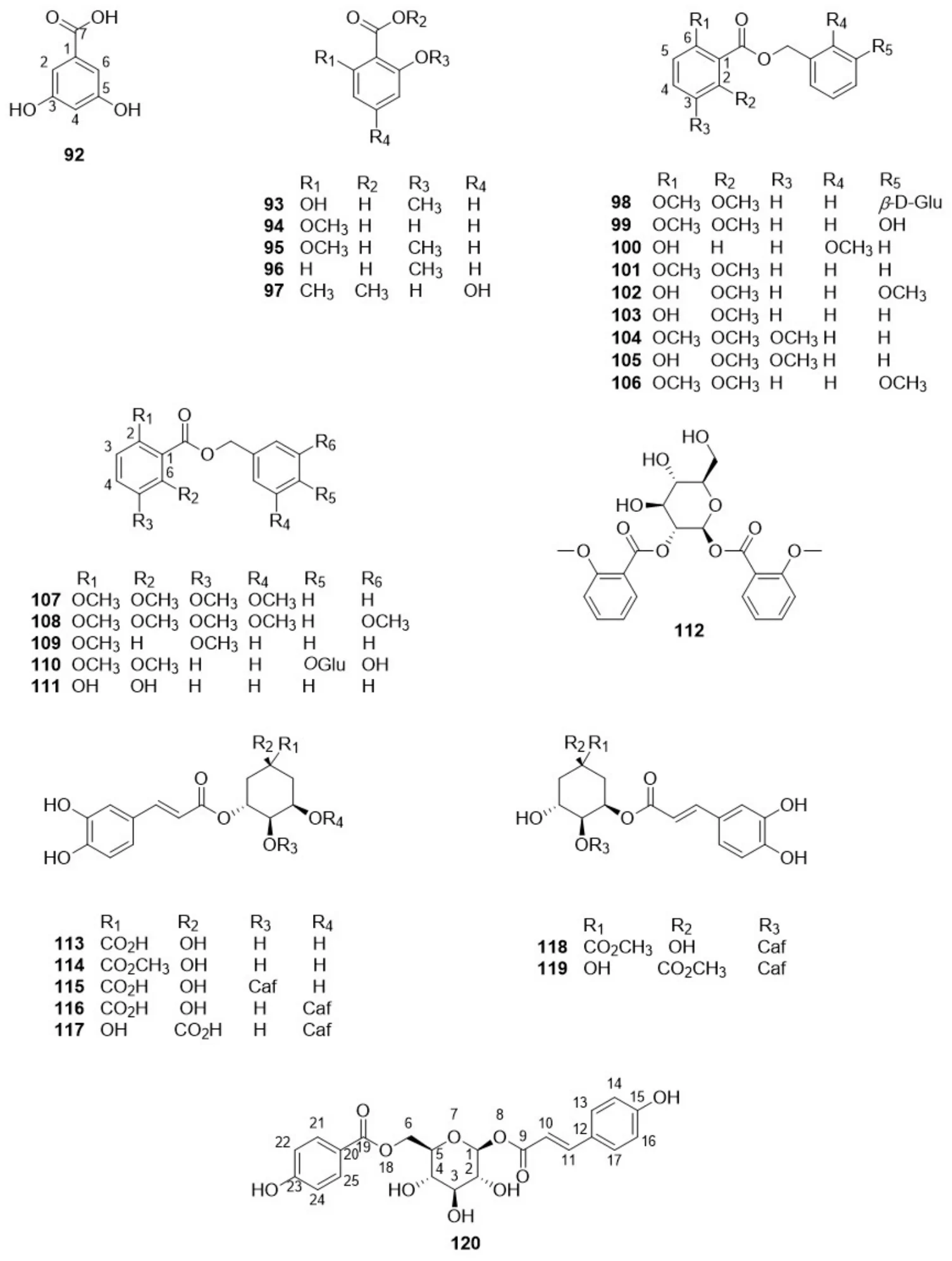

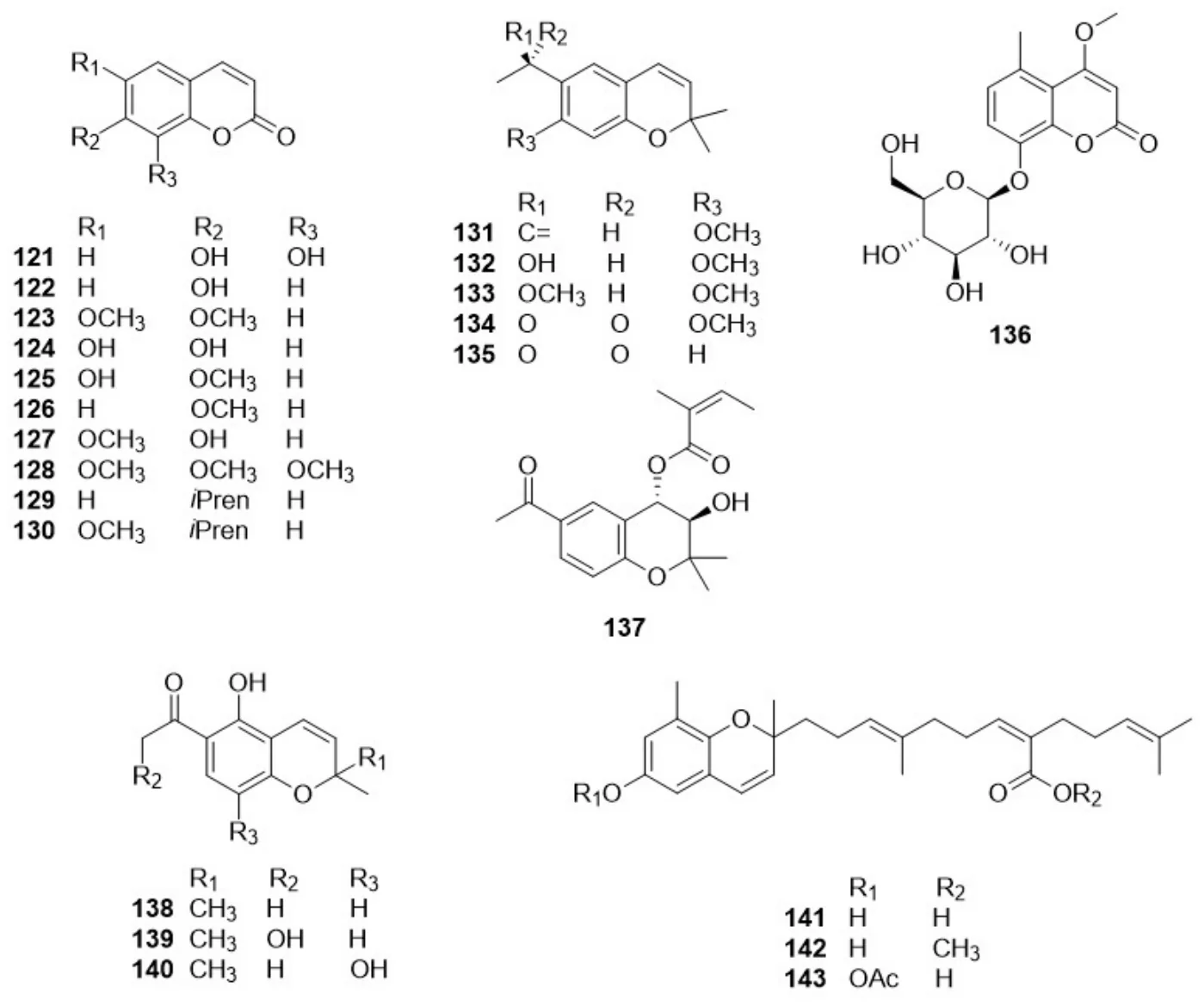

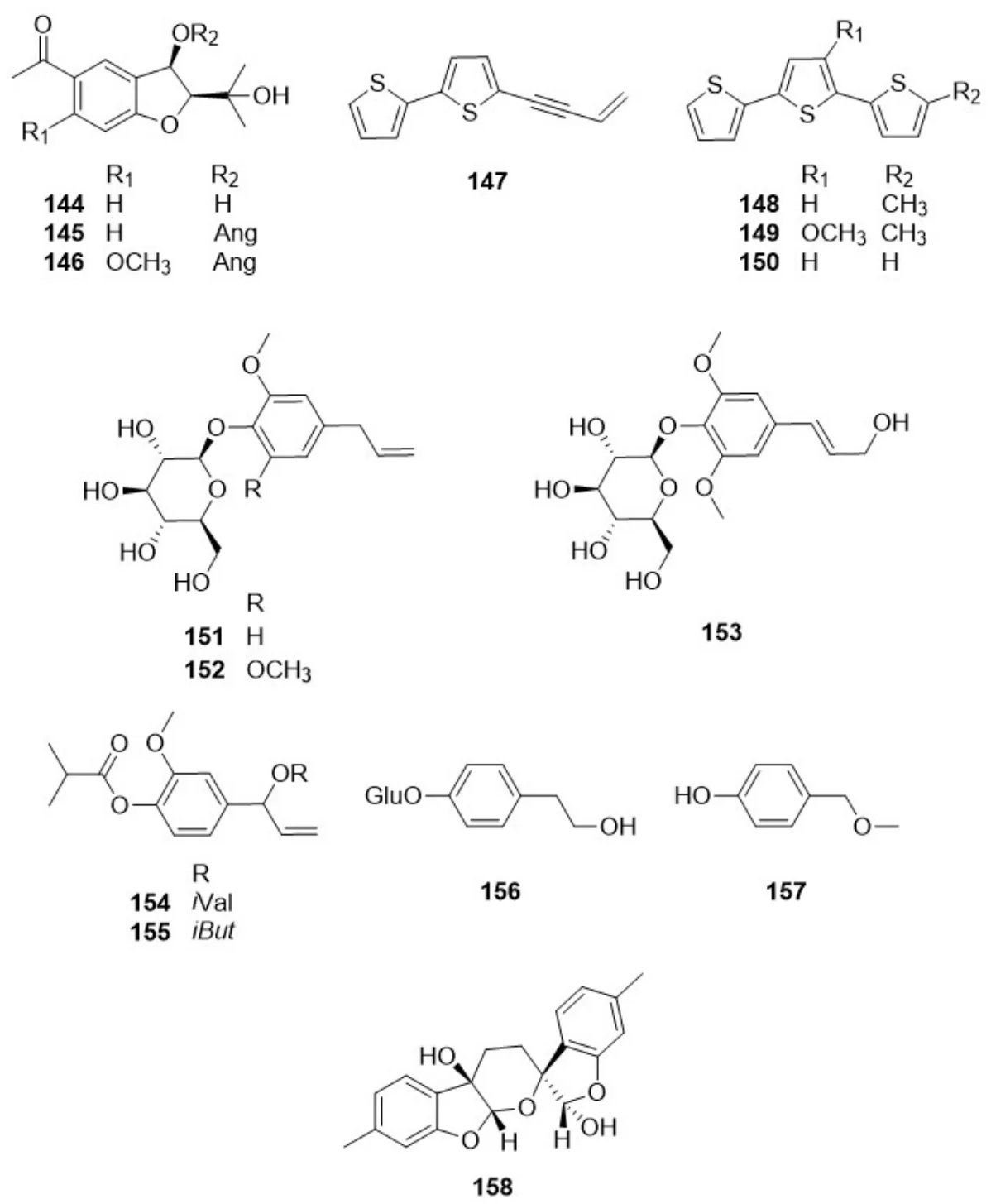
Phenolics and aromatic compounds derived from Mexican Asteraceae species.

**Figure 5:**
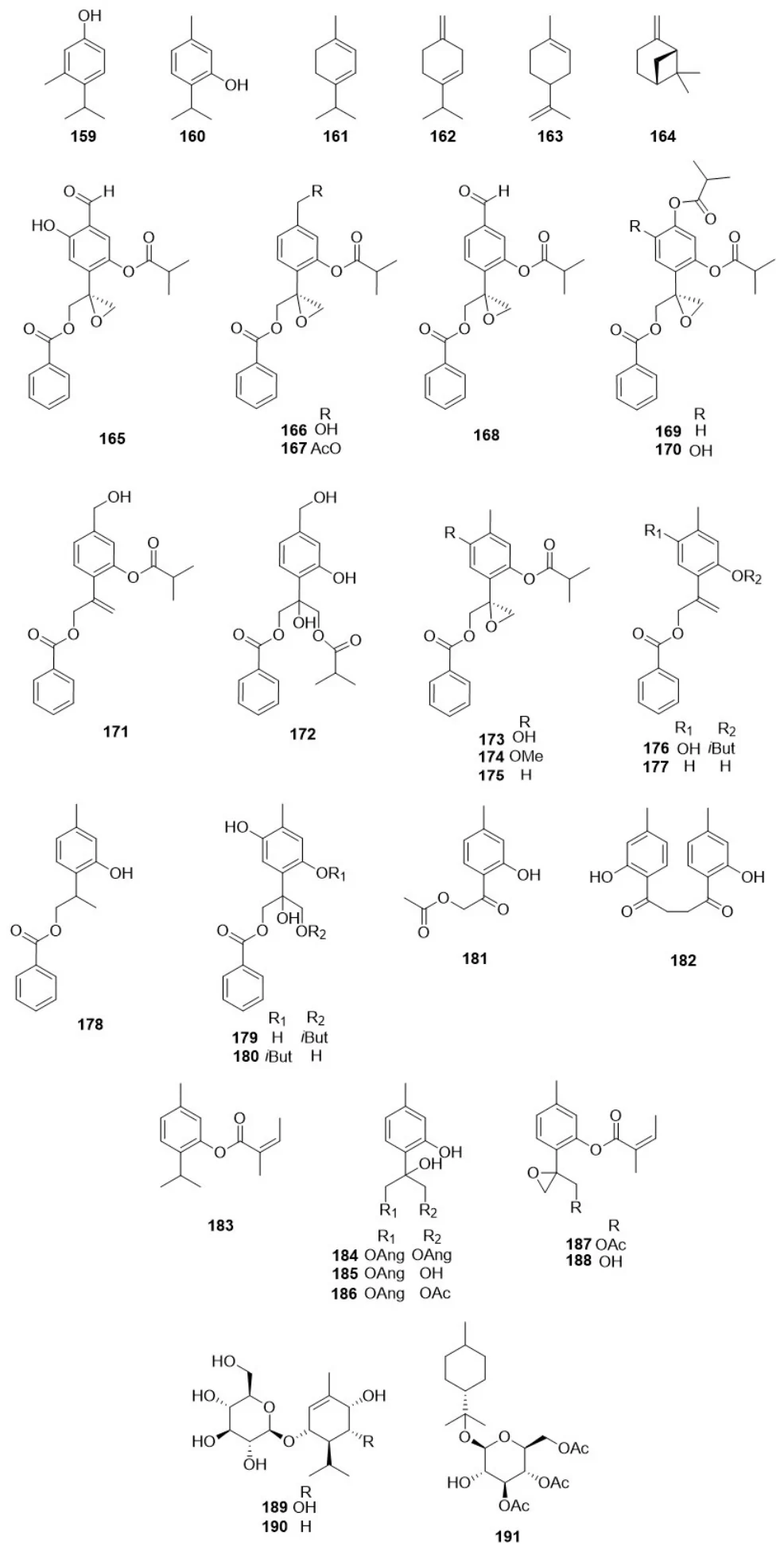
Monoterpenoids identified in Mexican Asteraceae species.

**Figure 6:**
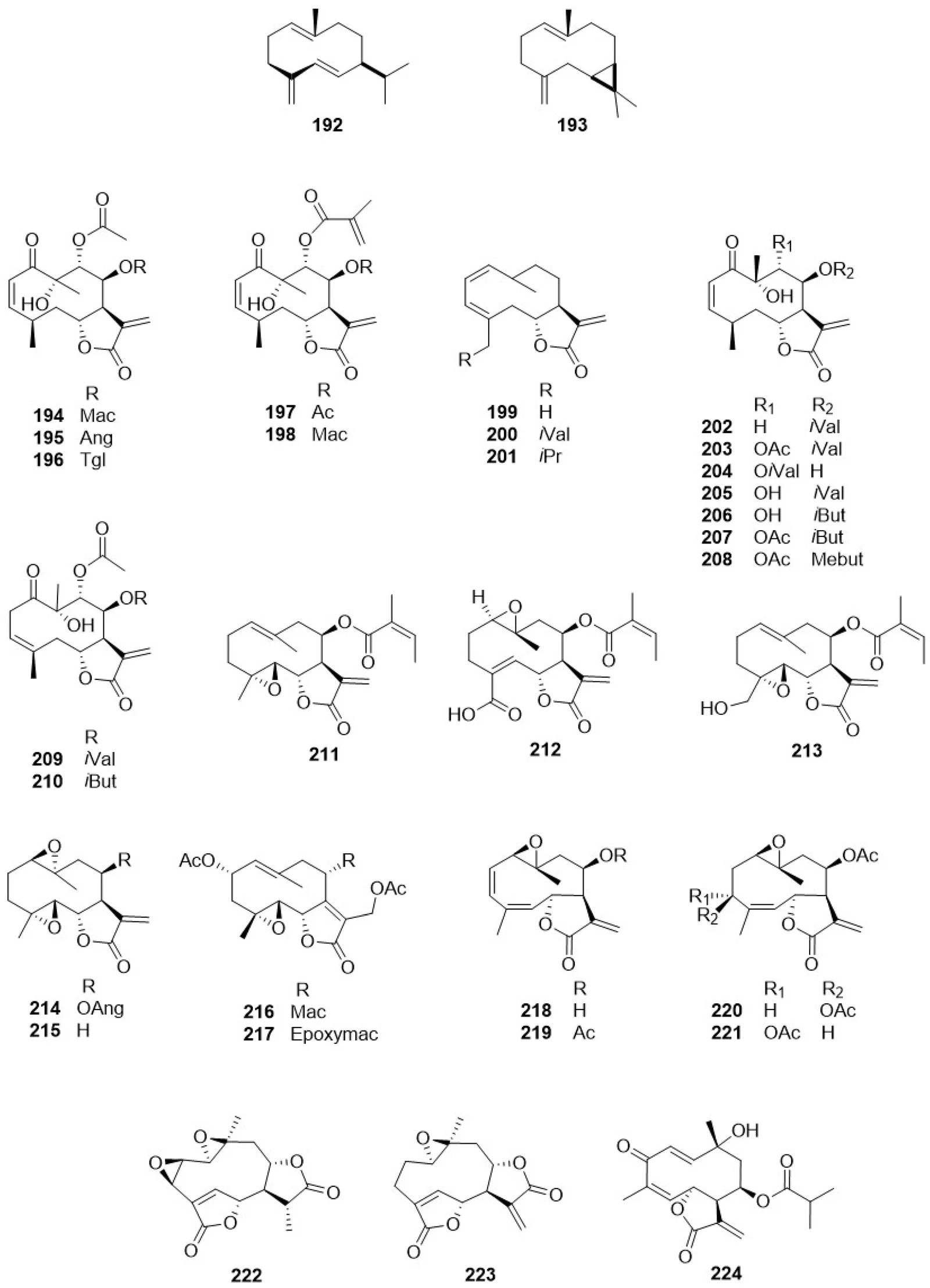

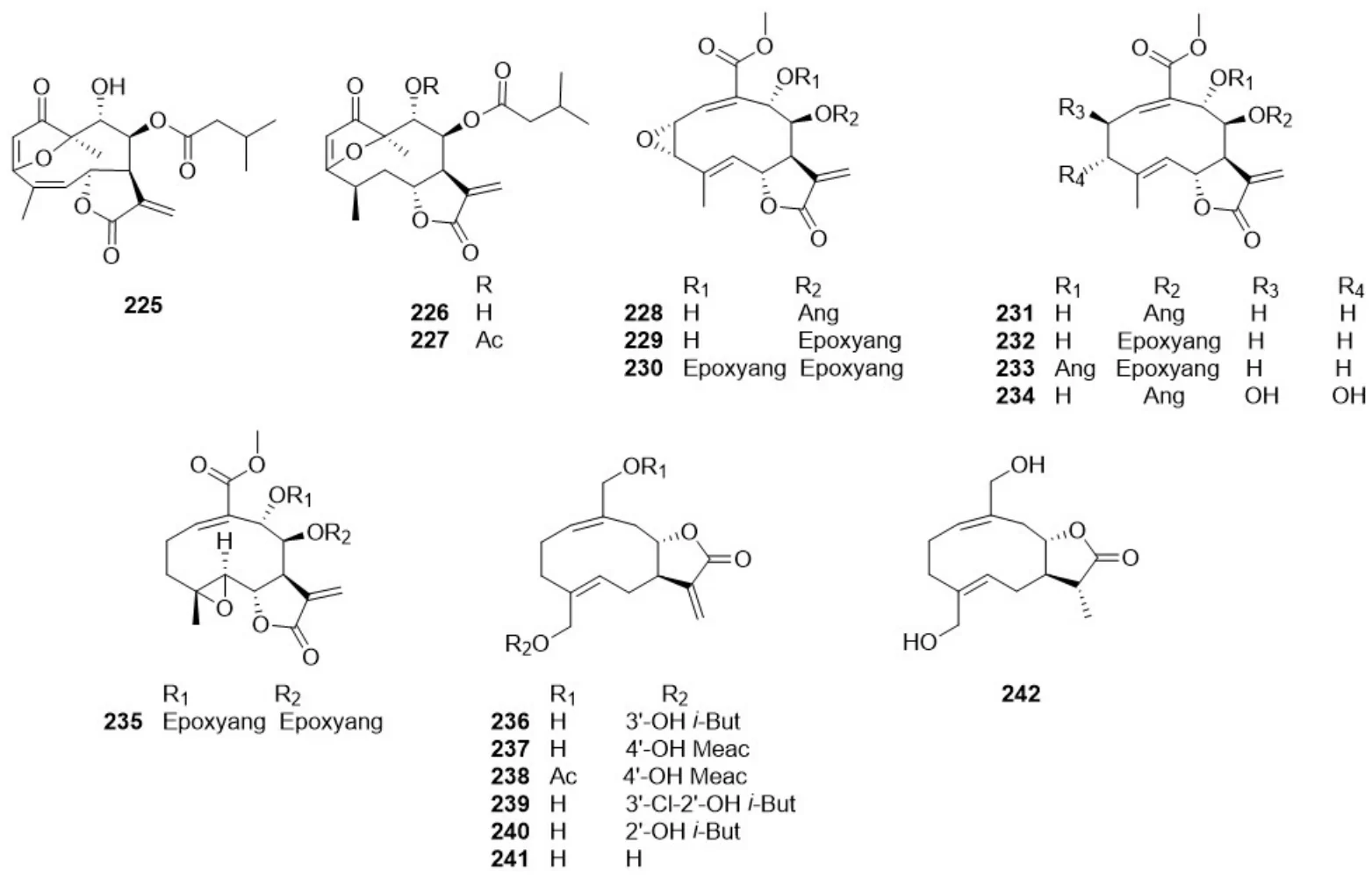
Principal sesquiterpenoids identified in Mexican Asteraceae species.

**Figure 7:**
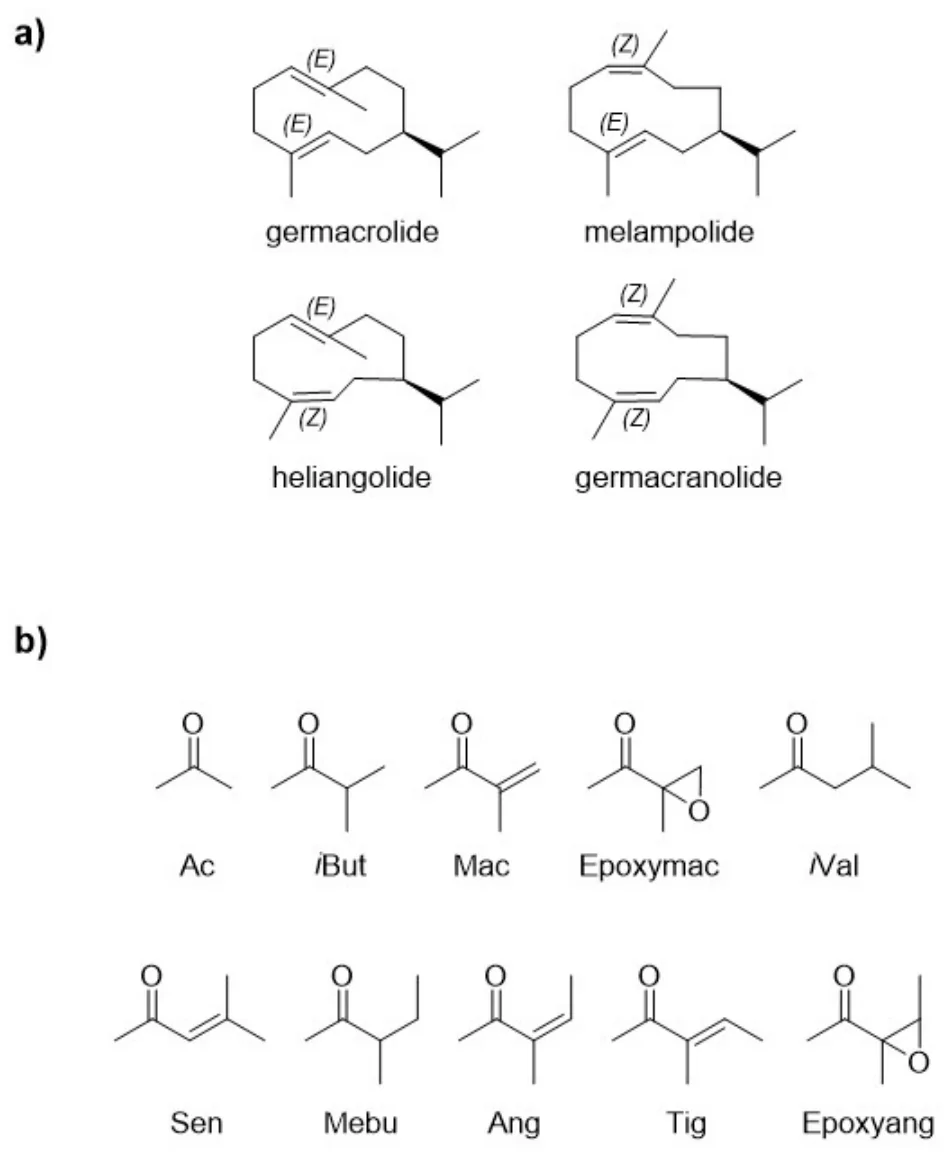
Types of germacranolides (**a**) and their natural acylation residues (**b**).

**Figure 8:**
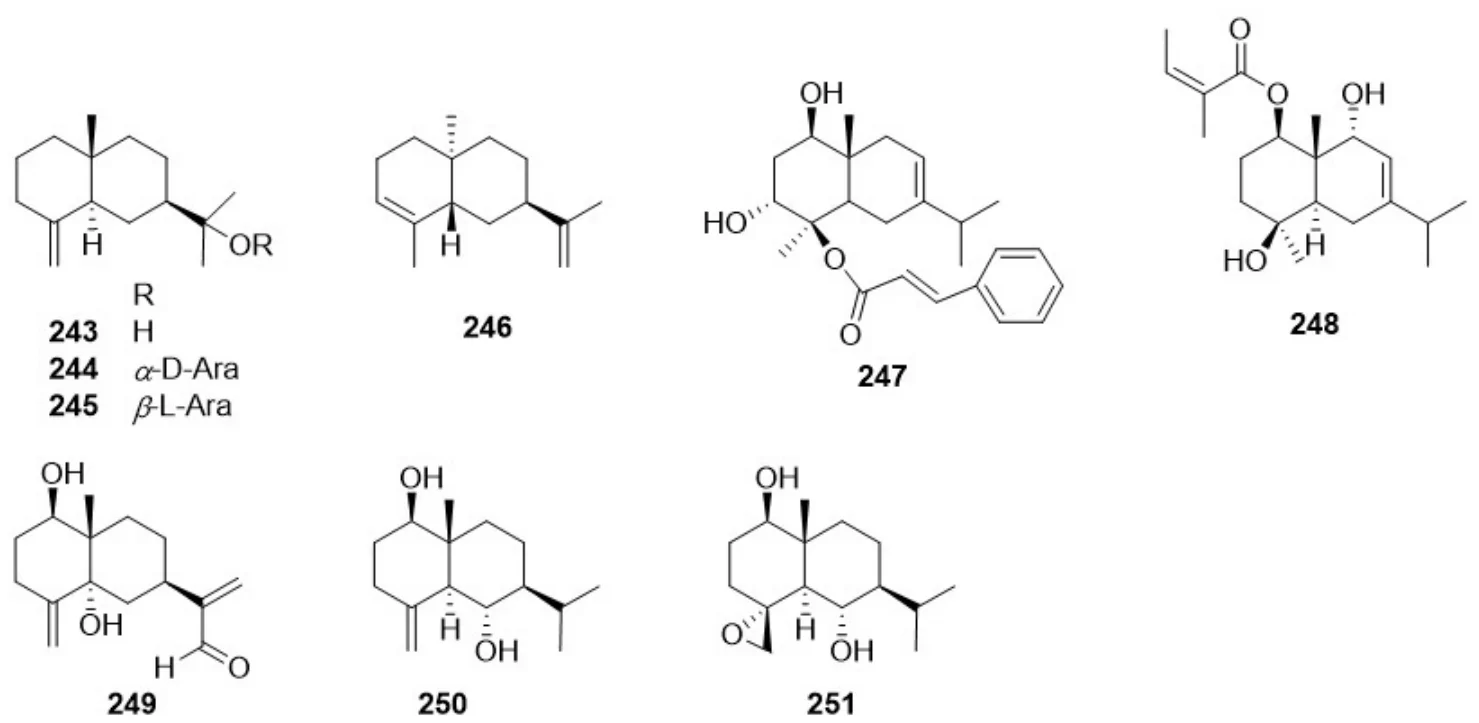
Eudesmanes from Mexican Asteraceae plants.

**Figure 9:**
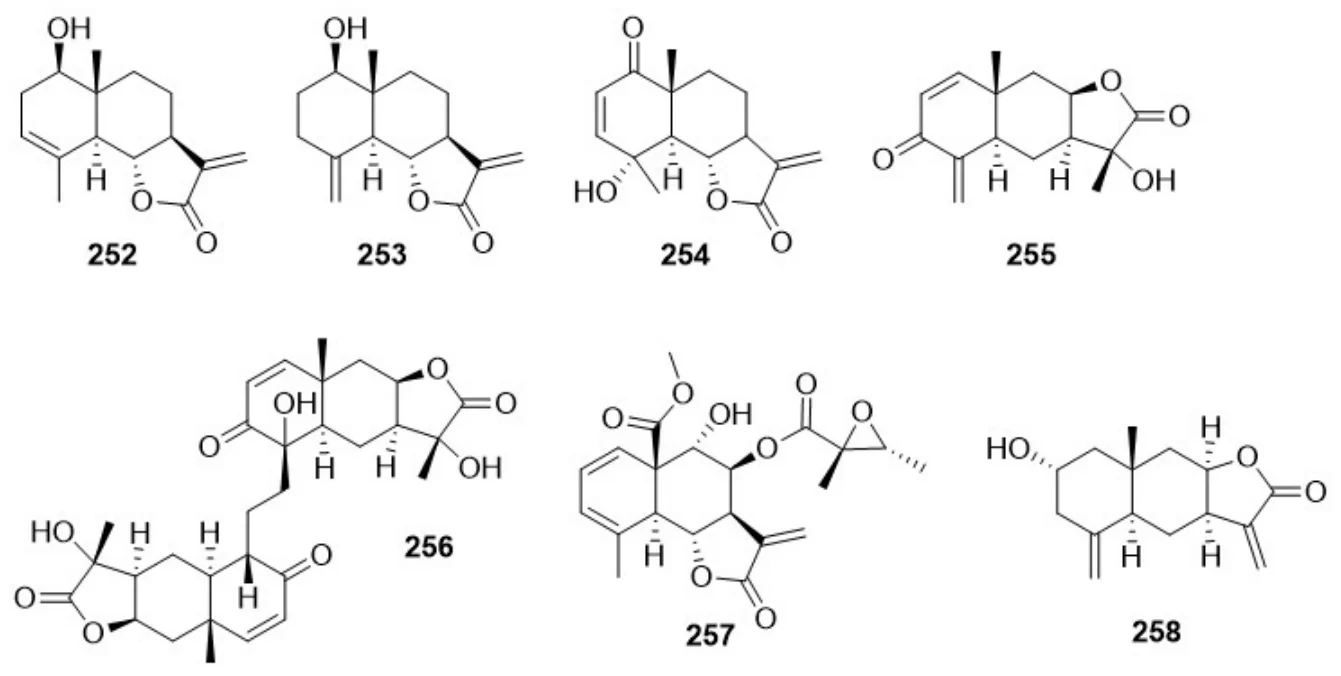
Eudesmanolides from Asteraceae plants native to Mexico.

**Figure 10:**
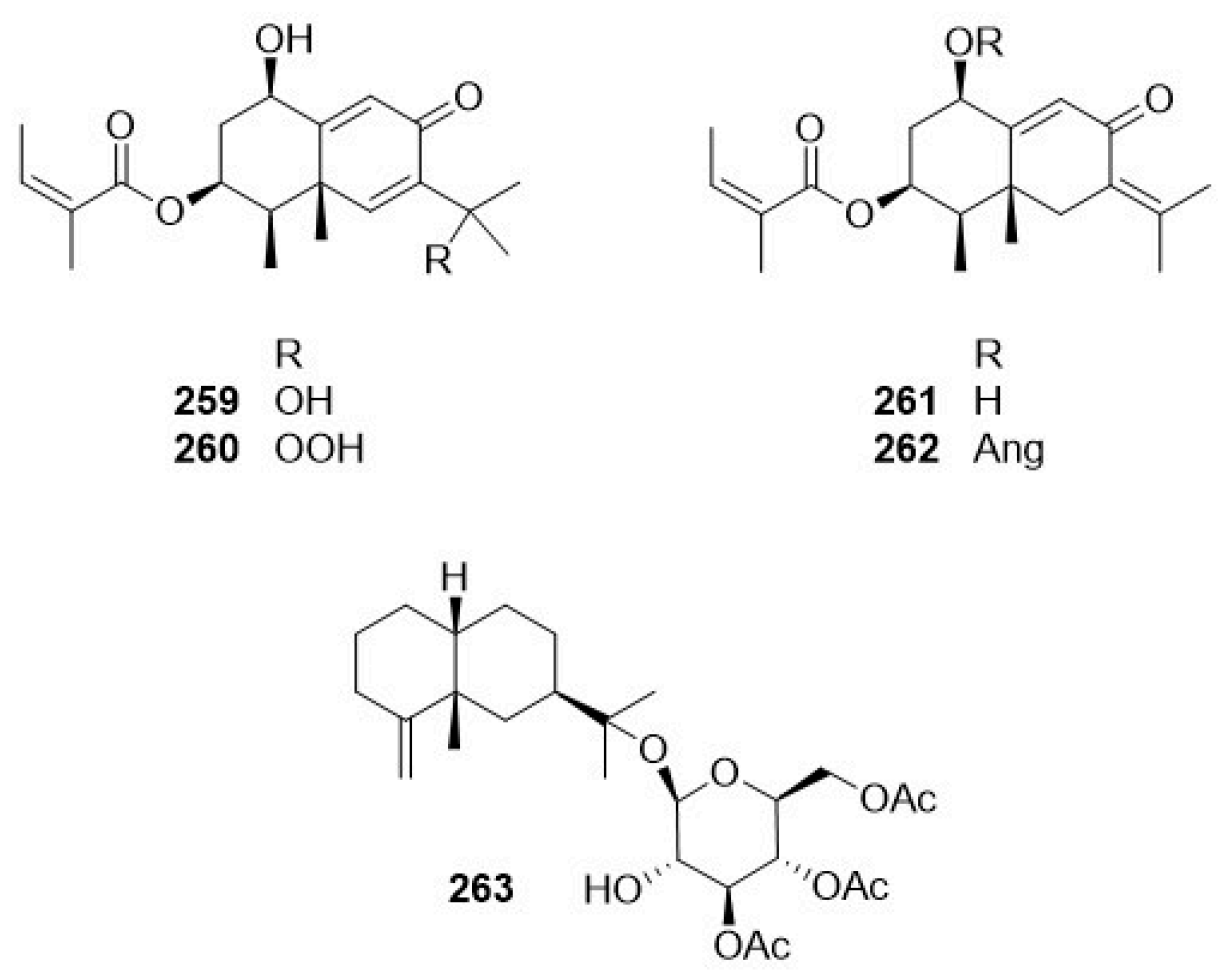
Eremophilane-type sesquiterpenes.

**Figure 11:**
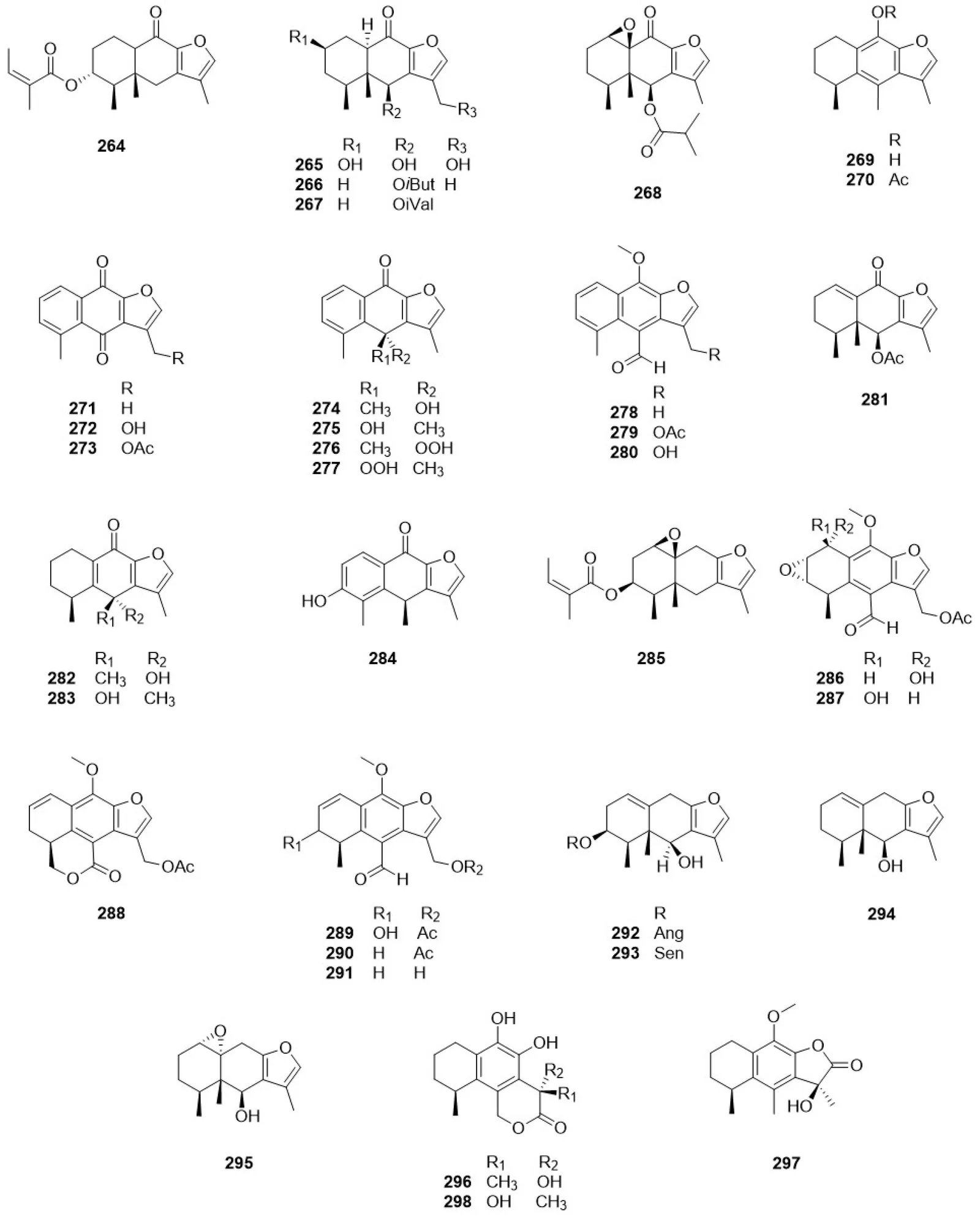
Furanoeremophilanes derived from Mexican Asteraceae species.

**Figure 12:**
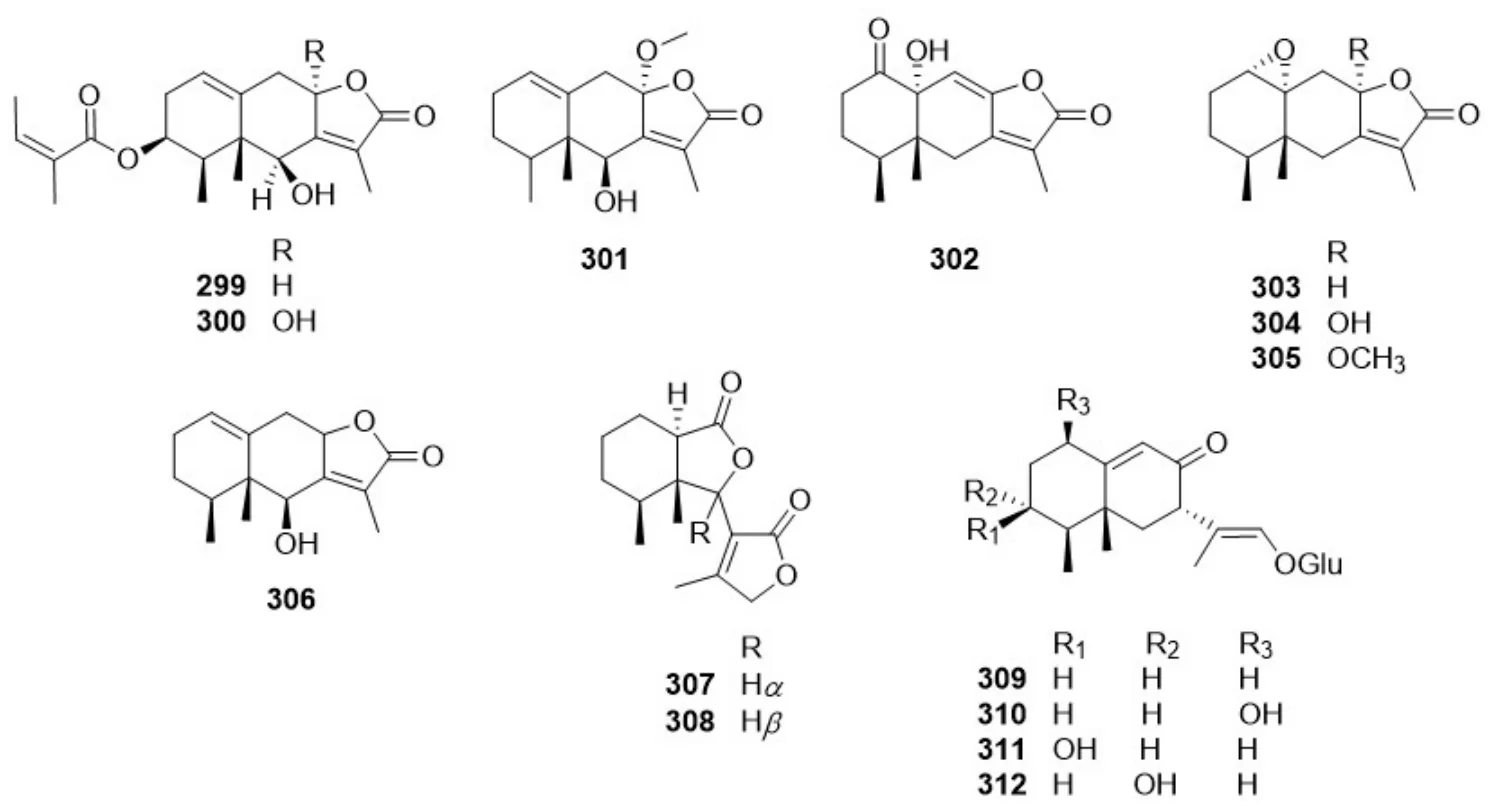
Eremophilanolides isolated from Asteraceae species.

**Figure 13:**
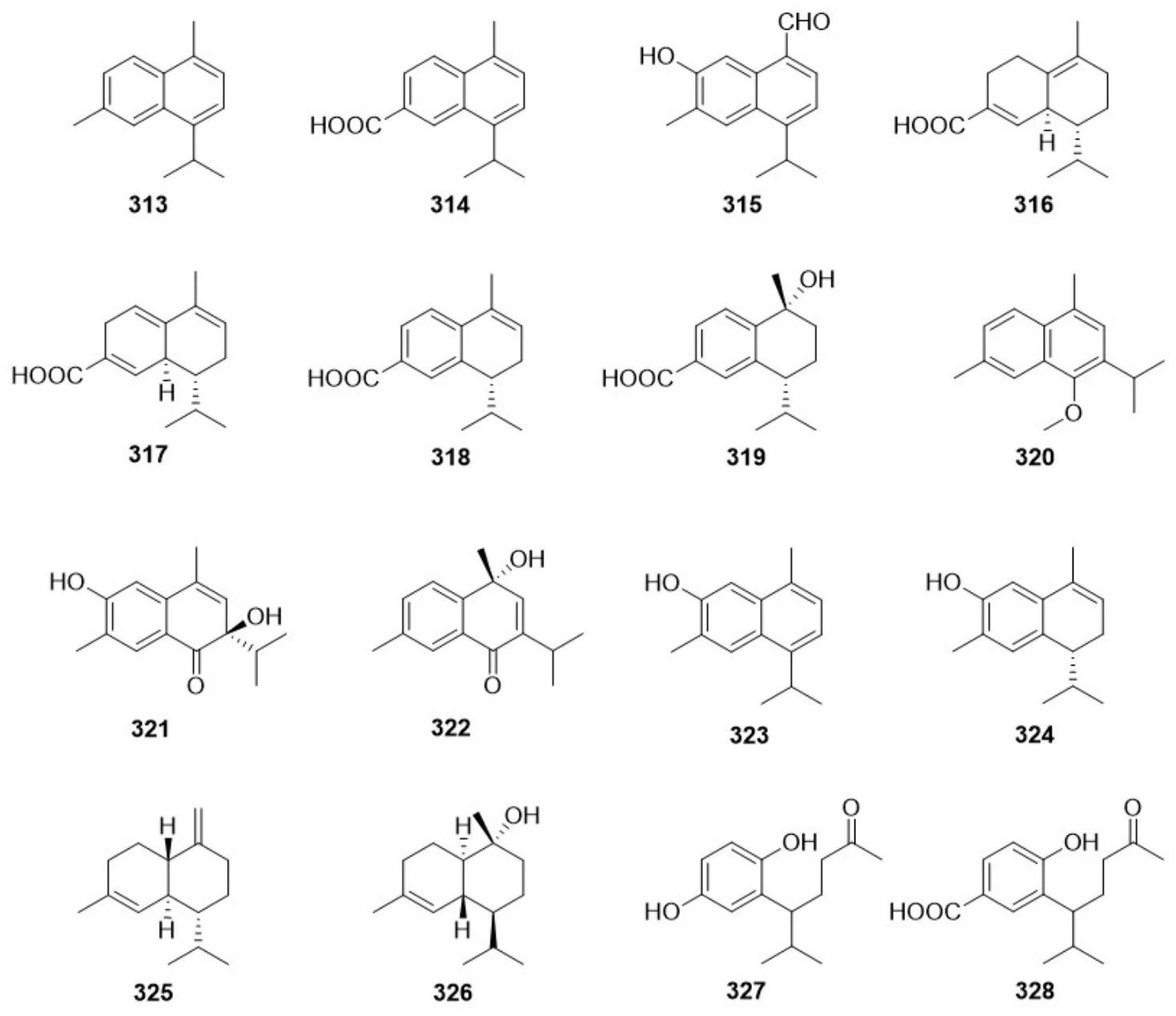
Cadalenes derived from Mexican Asteraceae species.

**Figure 14:**
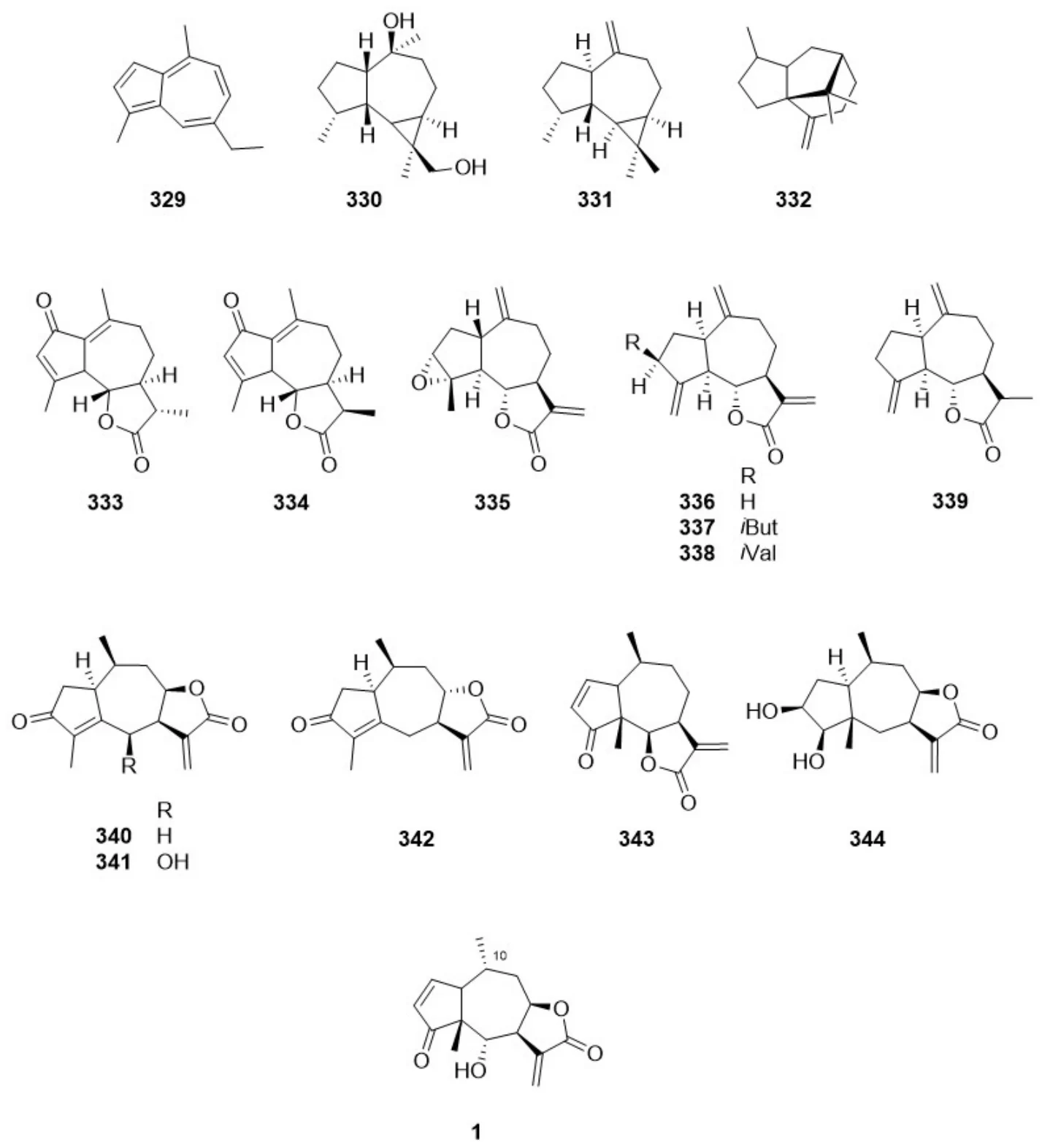
Guaianes and guaianolides from Asteraceae species.

**Figure 15:**
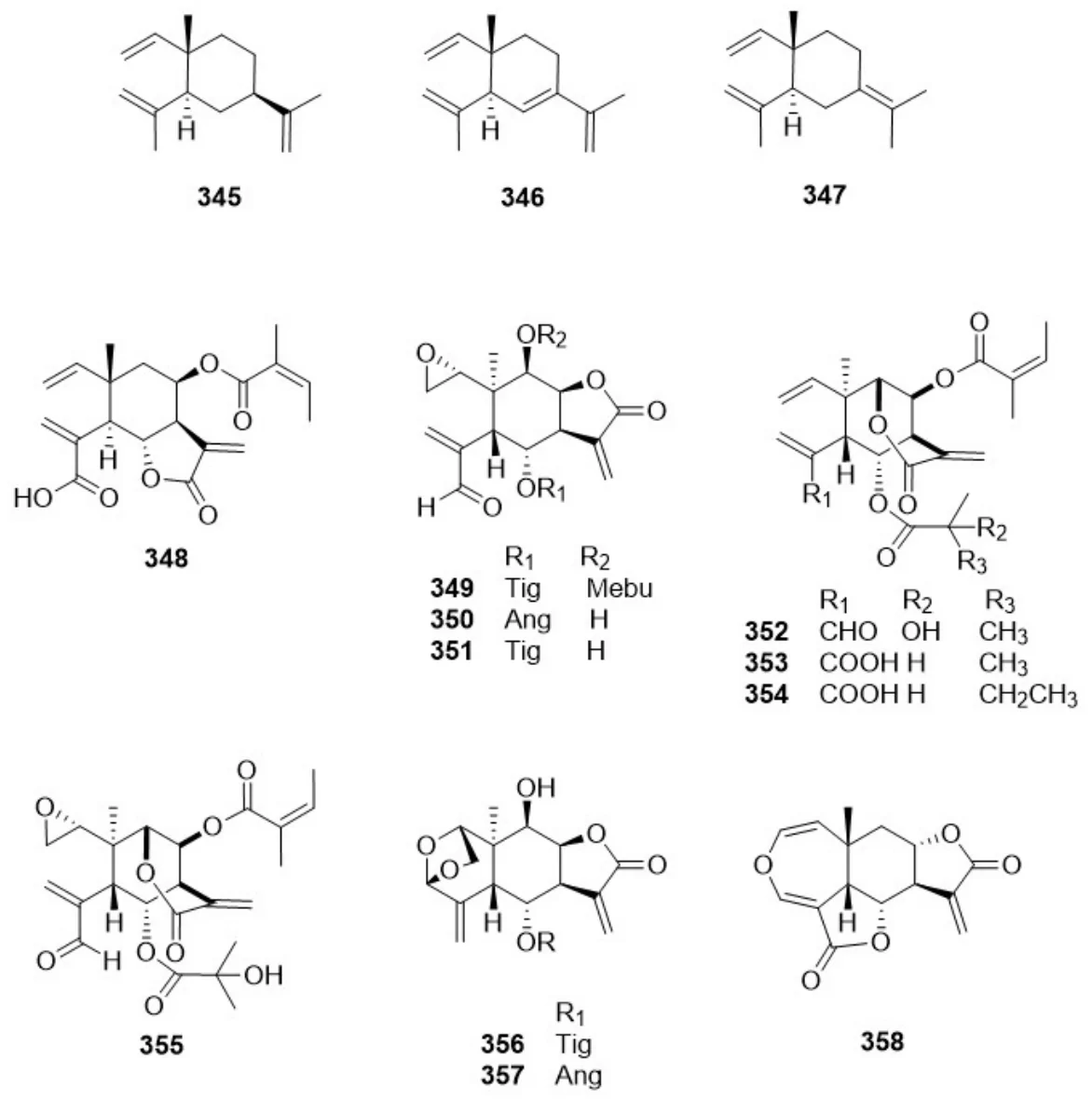
Elemenes and elemanolides derived from Mexican Asteraceae species.

**Figure 16:**
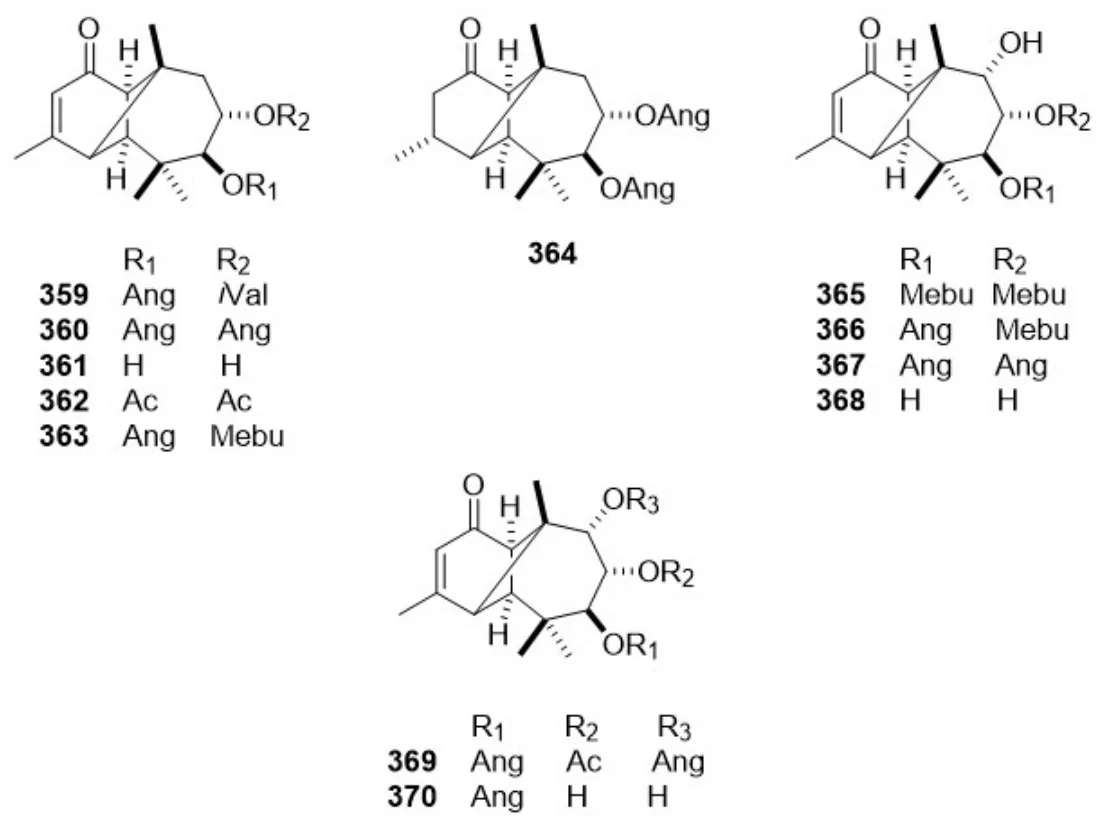
Longipinenes obtained from Asteraceae species.

**Figure 17:**
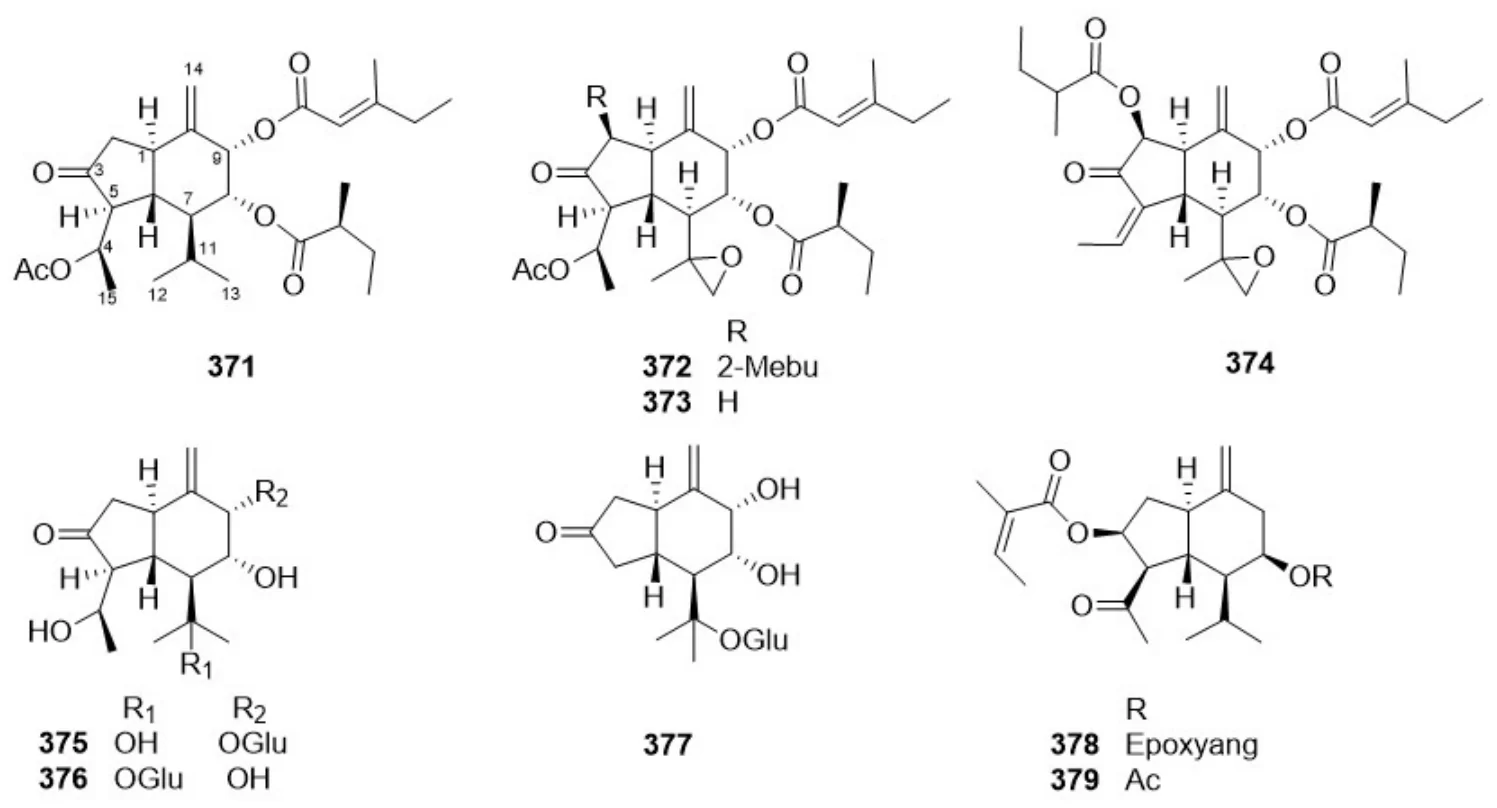
Oplopanes derived from *P. filare* and *R. gerberifoliu*.

**Figure 18:**
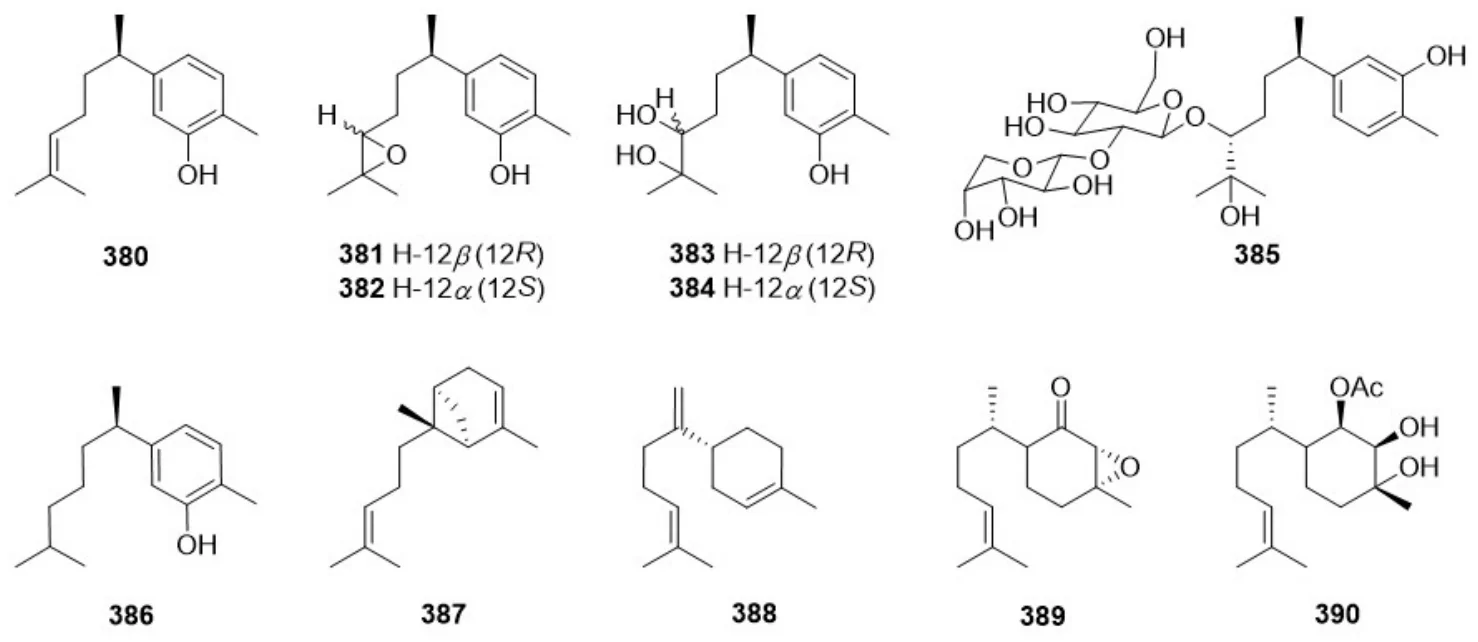
Bisabolenes isolated from Asteraceae species.

**Figure 19:**
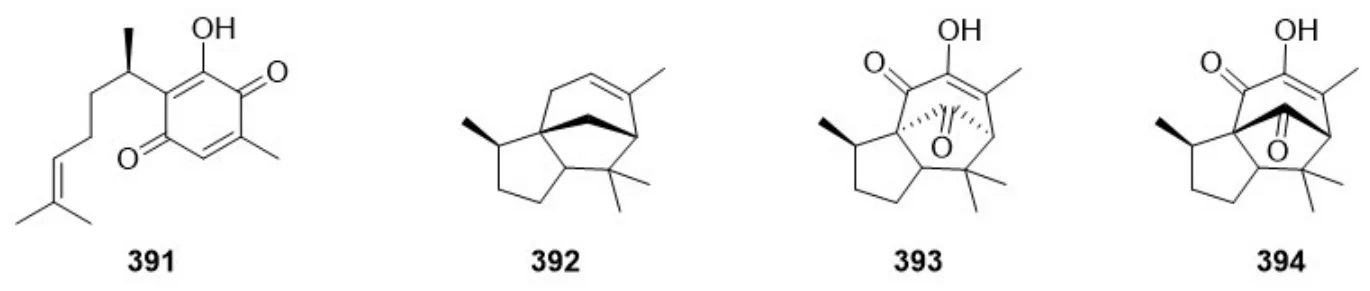
Cedrene (**394**), perezone (**393**) and its related sesquiterpenoids.

**Figure 20:**
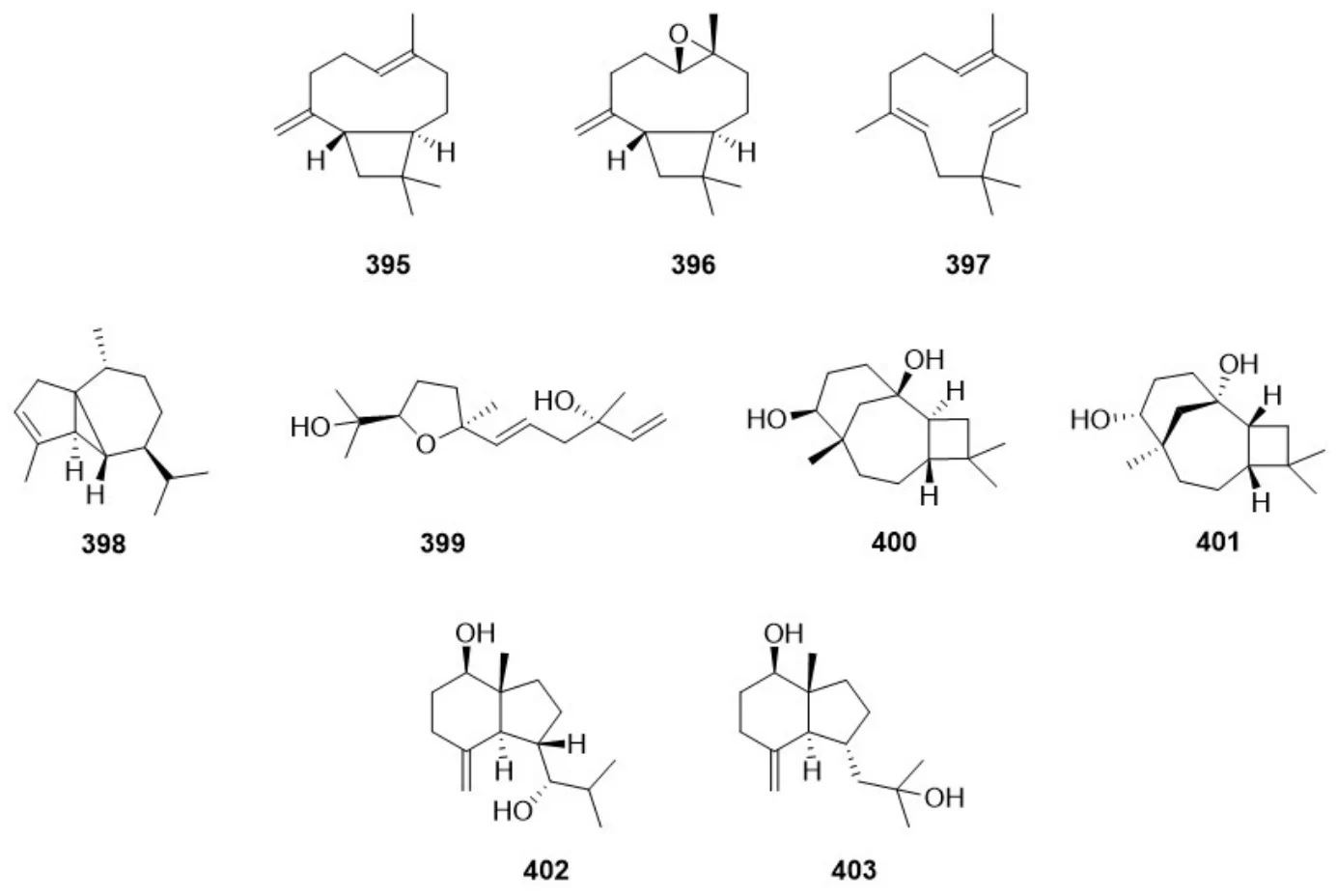
Additional sesquiterpenoid scaffolds from Mexican Asteraceae species.

**Figure 21:**
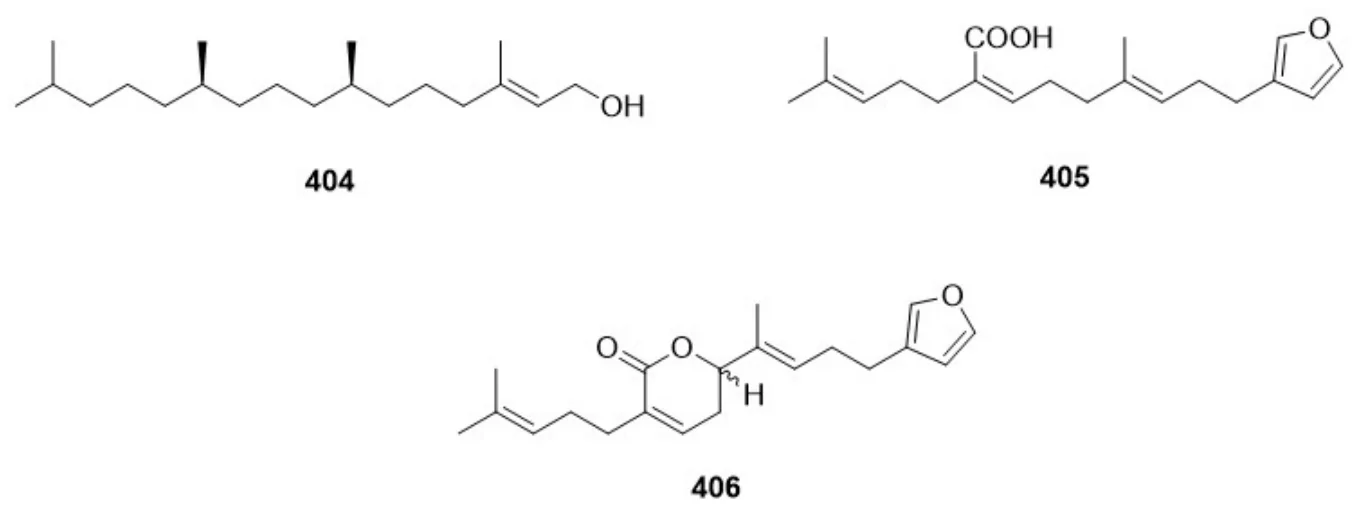
Acyclic and monocyclic diterpenes from Asteraceae species.

**Figure 22:**
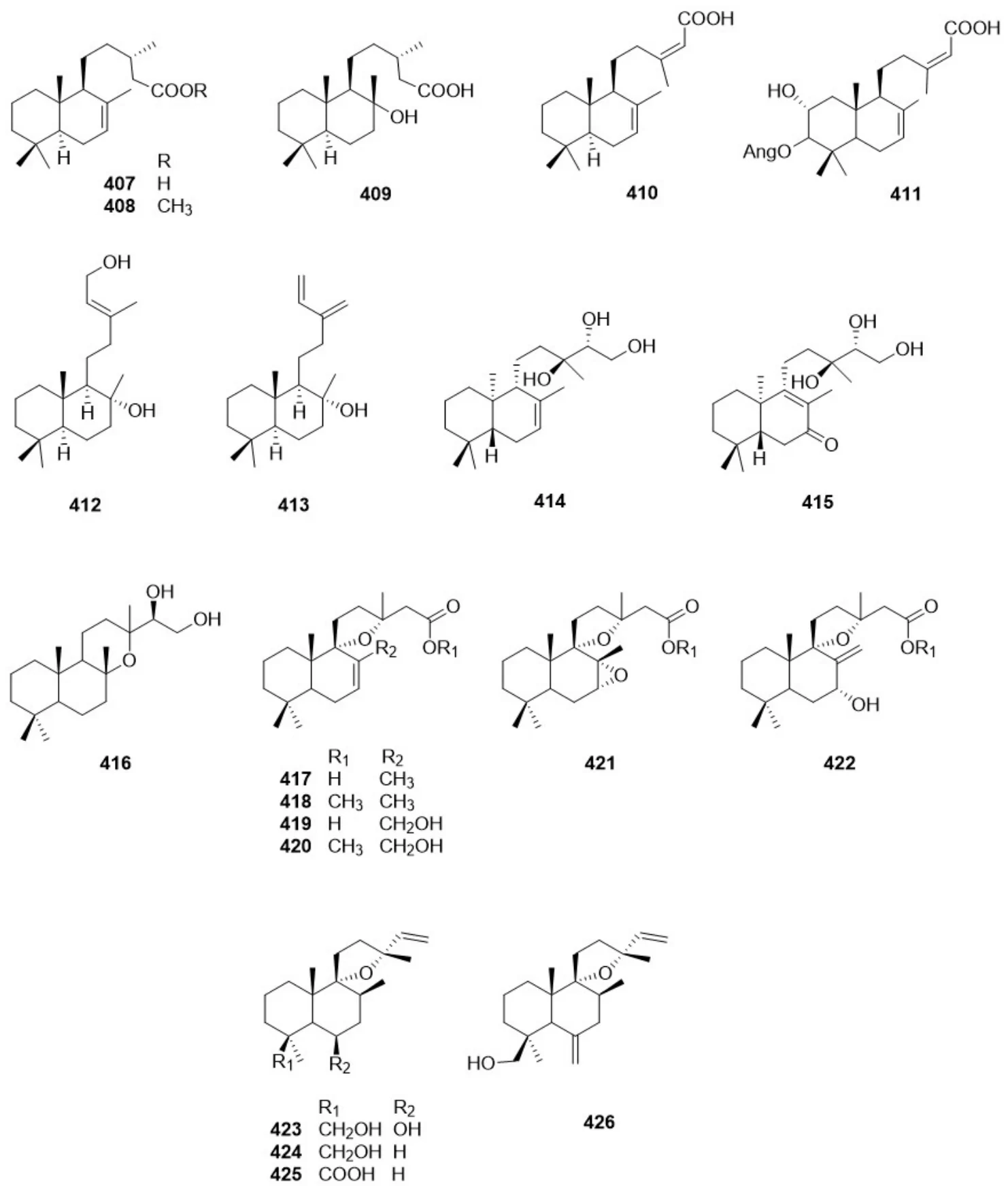
Labdanes from Mexican Asteraceae species.

**Figure 23:**
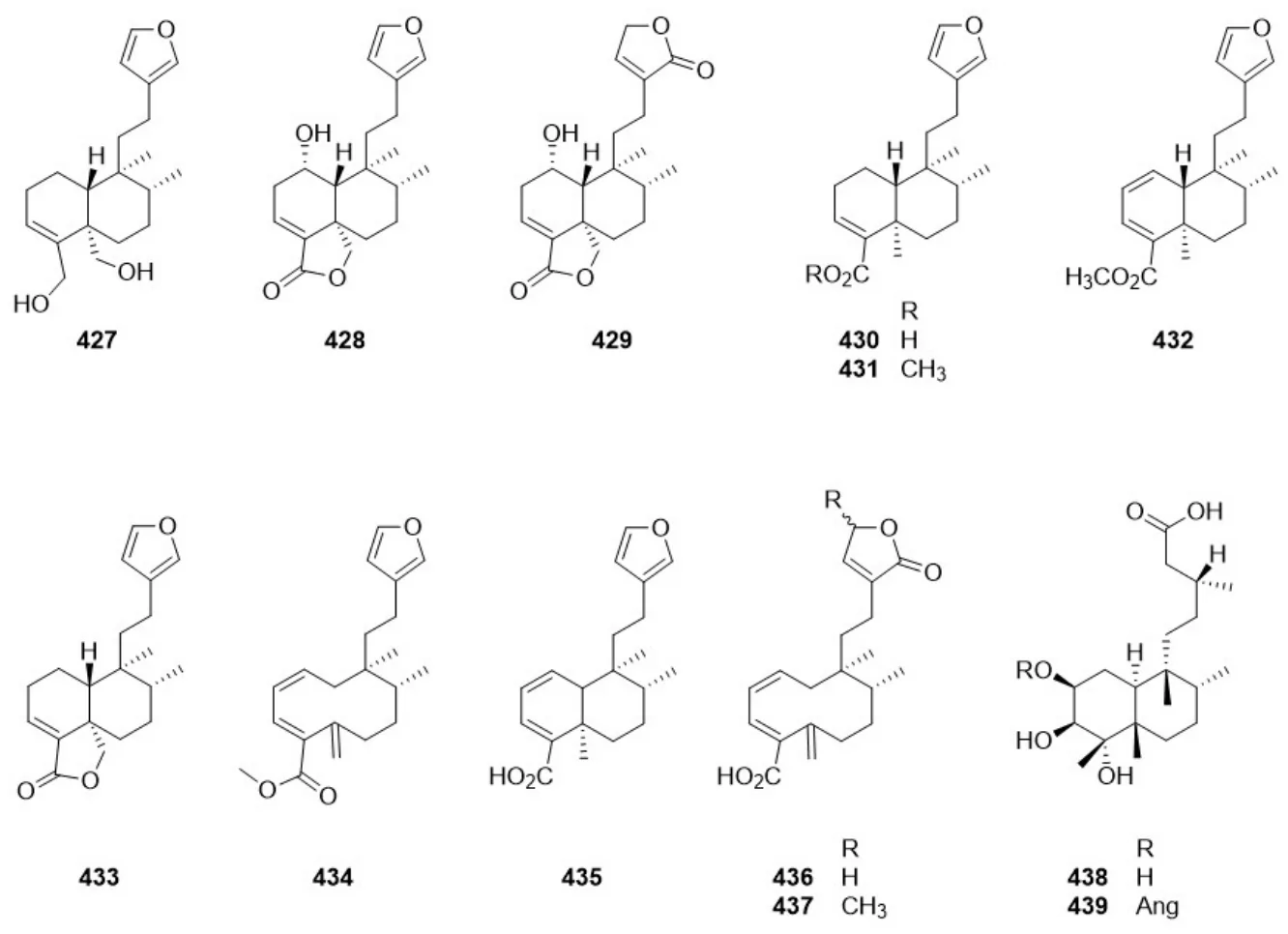
*Neo*-clerodane diterpenoids from Mexican Asteraceae species.

**Figure 24:**
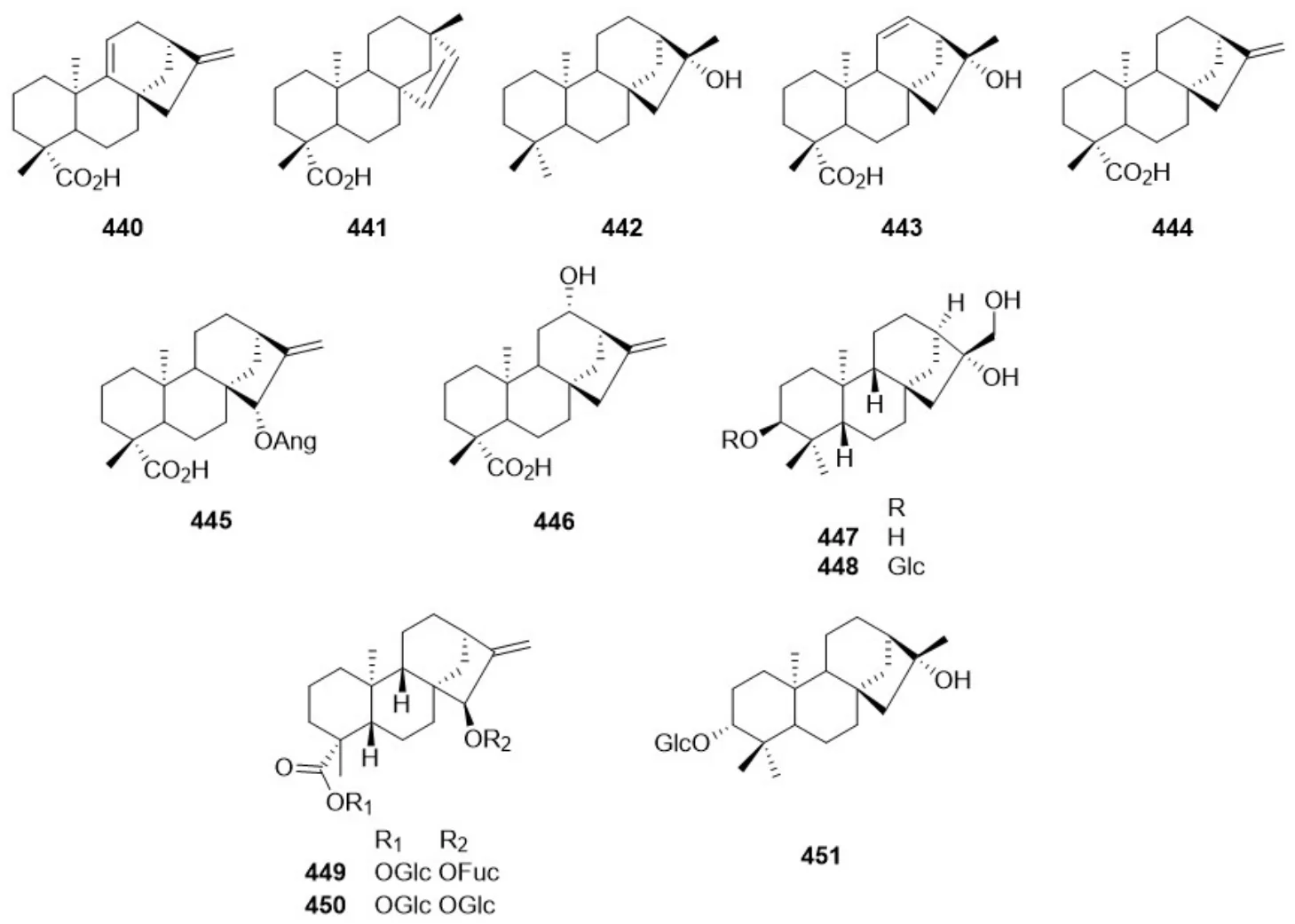
*Ent*-kaurane diterpenoids from Asteraceae species.

**Figure 25:**
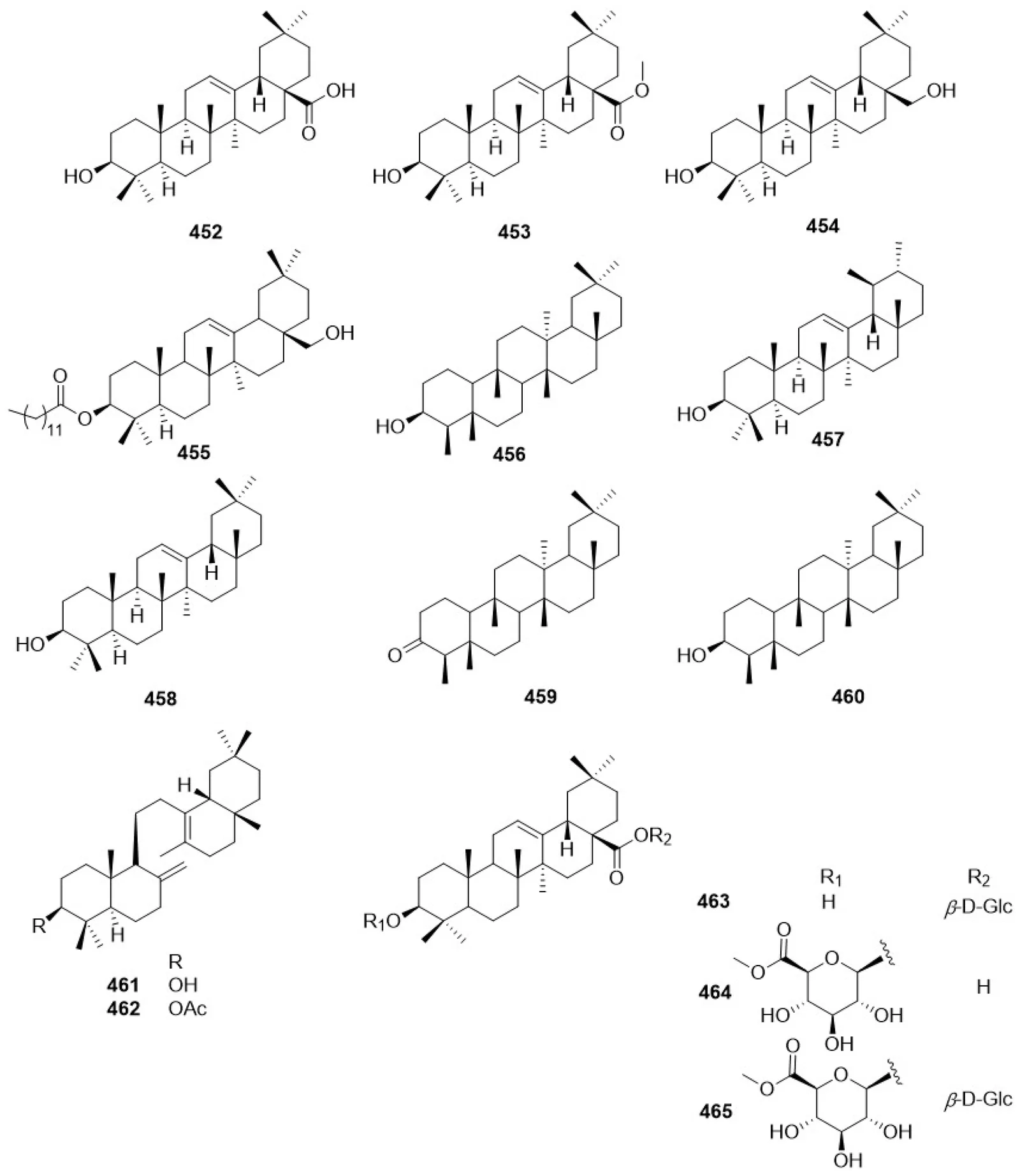
Oleanane-type triterpenoids from Asteraceae species.

**Figure 26:**
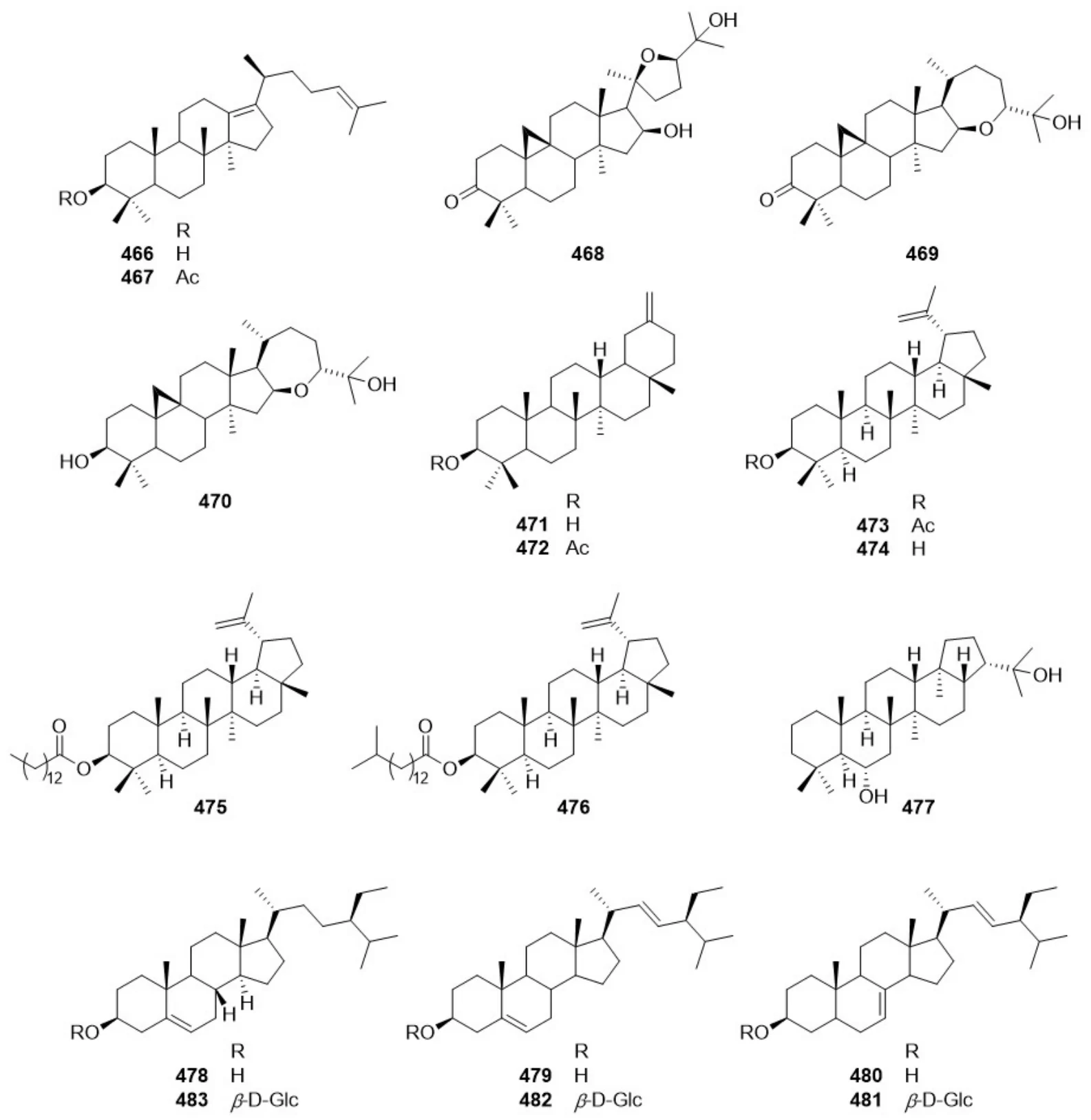
Additional triterpenoids and phytosterols from Mexican Asteraceae plants.

**Figure 27:**
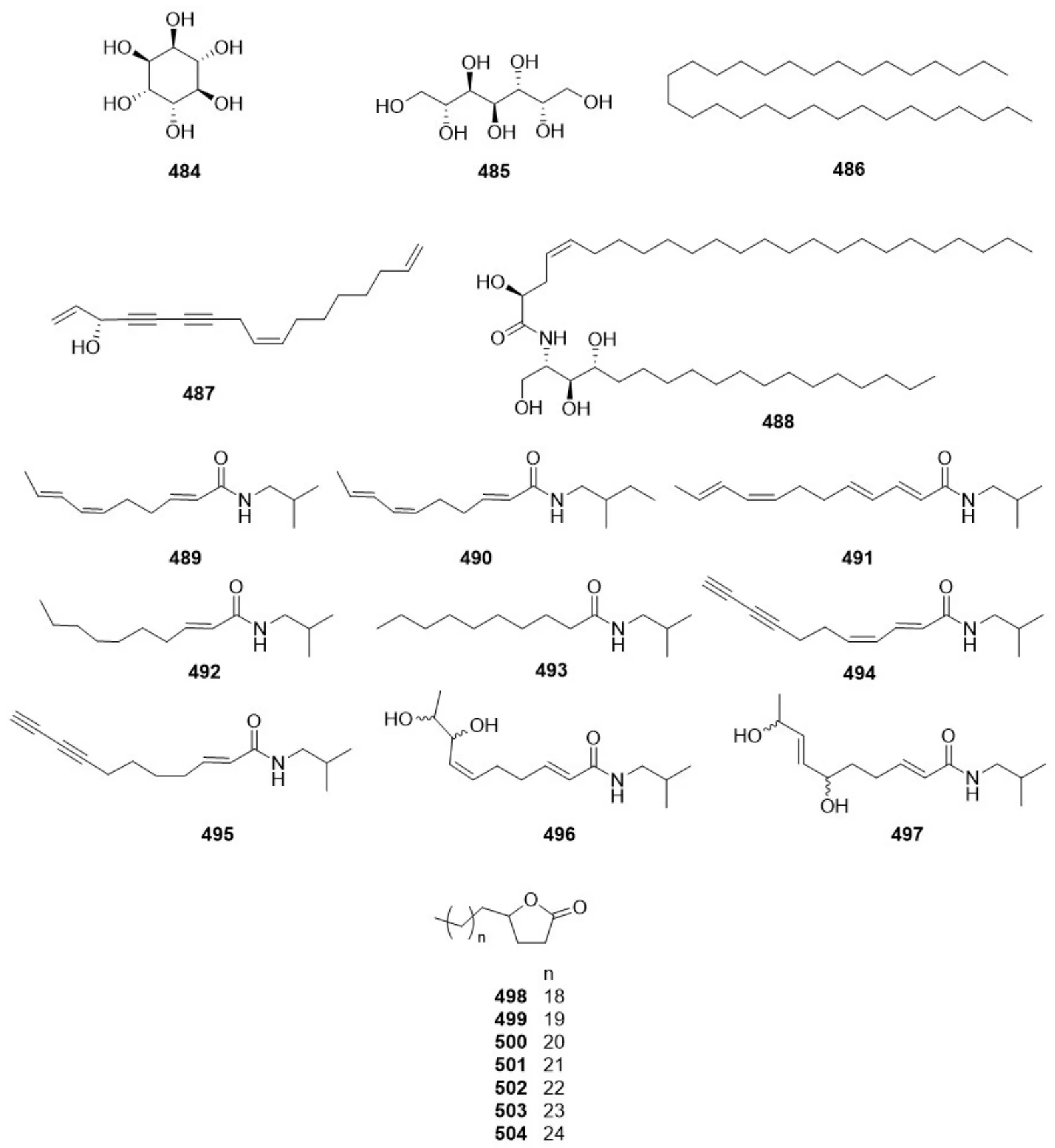
Miscellaneous specialized metabolites from Asteraceae species.

**Figure 28:**
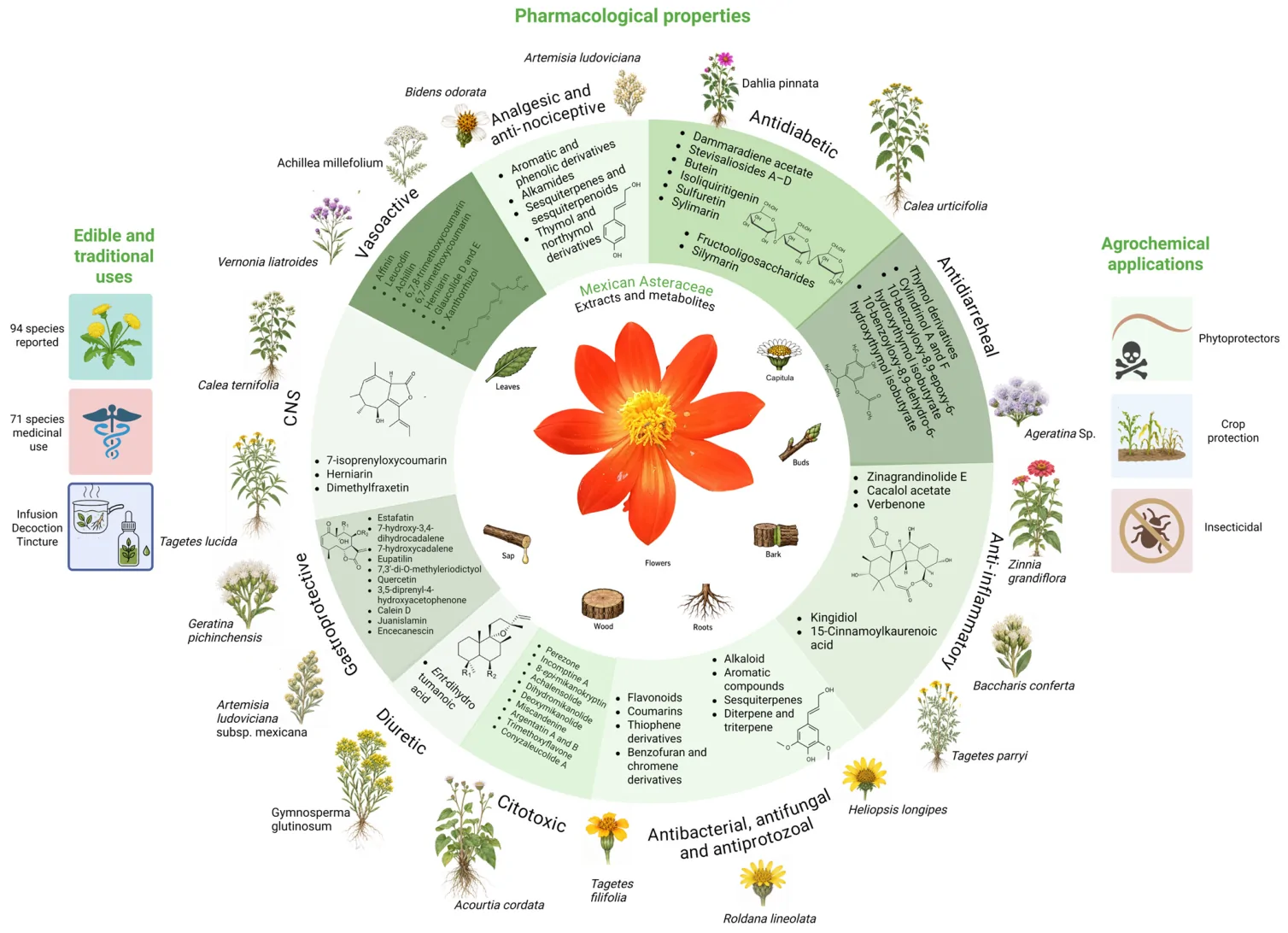
Traditional uses, phytochemical Diversity, pharmacological Properties, and agrochemical applications of species in the Asteraceae family native to Mexico. The figure shows which plant parts are used, the main bioactive compounds, key pharmacological activities, traditional medicinal uses, and agrochemical applications reported for the Asteraceae family.

**Figure 29:**
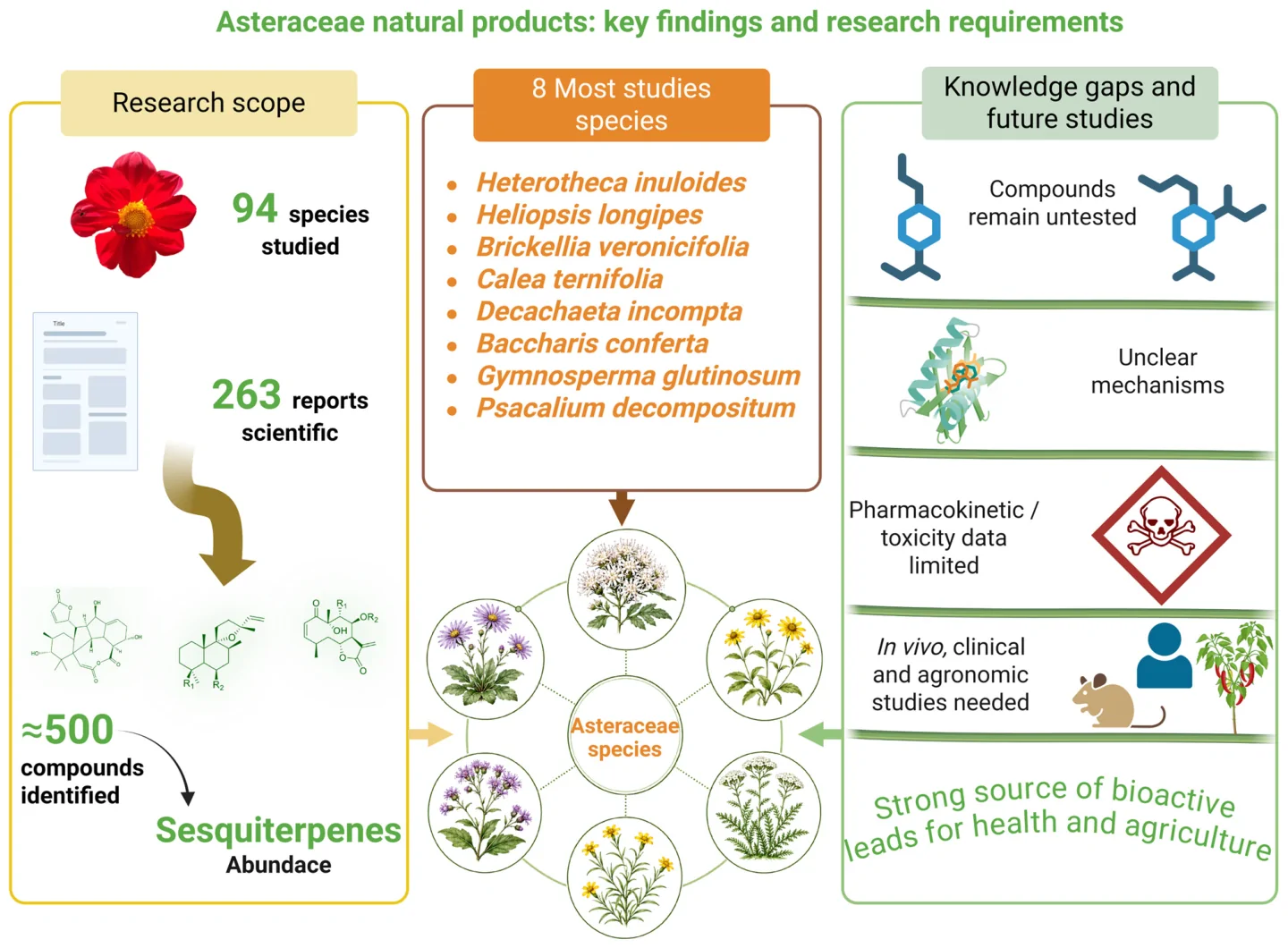
Current knowledge and future perspectives on Mexican Asteraceae plants. This figure summarizes the main findings of Mexican Asteraceae research, highlighting the most studied species, the predominance of sesquiterpenes, and the major knowledge gaps that should guide future pharmacological, toxicological, clinical, and agronomic studies.

## Data Availability

Data are available on request.
